# An Overview of Additive Manufacturing of Triply Periodic Minimal Surface (TPMS) Structures

**DOI:** 10.3390/polym17243307

**Published:** 2025-12-14

**Authors:** Md Sakhawat Hossain, Md Mosharrof Hossain, Sabrina Nilufar

**Affiliations:** School of Mechanical, Aerospace, and Materials Engineering, Southern Illinois University Carbondale, Carbondale, IL 62901, USA; mdsakhawat.hossain@siu.edu (M.S.H.); mdmosharrof.hossain@siu.edu (M.M.H.)

**Keywords:** additive manufacturing, triply periodic minimal surfaces, metamaterials, finite element analysis

## Abstract

Triply periodic minimal surfaces (TPMS) are mathematically defined minimal surfaces that exhibit zero mean curvature and repeat periodically along all three Cartesian axes. They integrate mathematically defined geometry with extensive functional adjustability. Their smooth, non-self-intersecting topology enables systematic control over relative density and improves load transfer efficiency within the lattice. Their large surface area-to-volume ratios further enhance specific energy absorption (SEA) and enable diverse functional uses. Recent developments in additive manufacturing (AM) have made it easier to create TPMS structures. As a result, they are now considered as the architected materials that combine biological, thermal, and mechanical functions within a single framework. This study presents a comprehensive overview of the major TPMS structures. It further highlights several AM techniques used for their fabrication and provides a critical evaluation of how geometric design, relative density, and post-processing influence their mechanical and thermal performances. This work also discusses recent developments in graded and hybrid TPMS structures. It further identifies the main challenges and future research directions related to multi-material additive manufacturing and data-driven topology optimization.

## 1. Introduction

Additive manufacturing (AM) is an advanced fabrication process that constructs three-dimensional components directly from computer-aided design (CAD) data through a sequential, layer-by-layer material deposition process [1]. Unlike conventional subtractive or formative methods, AM builds components layer upon layer. This approach enables designers to create intricate internal architectures and control material placement in a manner previously unattainable [2]. Although the term “3D printing” is often used to describe such processes, it is primarily associated with small-scale or prototyping applications [2]. In contrast, additive manufacturing refers to a family of digitally controlled industrial techniques that integrate design, simulation, and production within an integrated digital workflow [3]. This digital integration promotes mass customization, freedom of design, and efficient material utilization across polymeric, metallic, and ceramic materials [4]. However, AM still faces economic and productivity constraints due to slower build rates and higher per-unit costs compared with conventional high-volume manufacturing [5,6].

According to ISO/ASTM 52900 standards [7], AM technologies are classified into seven principal categories: vat photopolymerization (VPP), material extrusion (MEX), material jetting (MJ), powder bed fusion (PBF), binder jetting (BJ), direct energy deposition (DED), and sheet lamination (SL) [1,8]. Each process operates under different physical principles, using different energy sources and feedstock forms to build the material. This technological diversity allows the fabrication of complex, functionally graded, and multi-material components with high precision. Over the past decade, continual advances in laser optics, photopolymer chemistry, powder metallurgy, and in situ process monitoring have significantly expanded the capabilities of AM. As a result, the technology has progressed from a prototyping tool to a reliable pathway for producing functional parts with consistent structural performance [4,9,10].

A particularly significant outcome of this progress is the enhanced ability to manufacture materials with designed internal structures and adjustable mechanical and physical behavior. Among these, triply periodic minimal surfaces (TPMS) have become an important class of geometrically continuous structures. They are defined by mathematical equations and repeat periodically in three dimensions while maintaining zero mean curvature [11]. TPMS structures are characterized by their smooth, non-self-intersecting surfaces and fully interconnected networks. These features promote uniform stress distribution and support efficient load transfer compared with conventional strut-based lattices. Their high surface area-to-volume ratios and continuous channels also enable superior heat and mass transport, making them suitable for structural, thermal, and biomedical applications [12,13,14,15].

In nature, TPMS-like geometries appear in cellular membranes, corals, butterfly wings, and cancellous bone, providing inspiration for bio-mimetic material design. When combined with AM, these geometries can be precisely fabricated at multiple length scales, allowing the optimization of relative density, pore size, and mechanical anisotropy for specific functional requirements [16,17,18]. The combination of digital design, mathematical modeling, and AM processing has allowed TPMS to connect material science with geometry-based design approaches.

This review aims to provide a comprehensive overview of the AM of triply periodic minimal surface (TPMS) based metamaterials, focusing on the interdependence between geometry, processing parameters, and functional performance. It summarizes recent developments in fabrication strategies, material selection, and structural optimization. It also explains how different design parameters and post-processing steps shape the mechanical, thermal, and transport performance of TPMS structures. The paper also discusses critical challenges in reproducibility, surface integrity, and multi-material integration that currently limit large-scale deployment. Finally, the review outlines emerging research directions. These include data-driven design, multi-physics modeling, and sustainable manufacturing approaches that can help move TPMS architectures from laboratory studies toward practical engineering applications.

## 2. Different TPMS Structures

Mathematicians Hermann Amandus Schwarz and Edvard Rudolf Neovius were pioneers in studying TPMS in the 19th century. Since then, several types of TPMS have been discovered and extensively researched. Alan Schoen presented 12 new TPMS based on skeleton graphs spanning crystallographic cells [11]. Schoen’s surfaces were unknown primarily in mathematics until Hermann Karcher established their mathematical proof in 1989 [19].

The early studies of TPMS stemmed from mathematical curiosity about surfaces with zero mean curvature geometries that minimize area while maintaining periodicity in three dimensions [11,17]. These surfaces later attracted engineering interest due to their inherent bicontinuous nature, which divides space into two interwoven but non-intersecting regions. Such geometry enables efficient transport of fluids, ions, or heat through continuous channels while preserving structural rigidity. Their high surface area to volume ratio and curvature-driven stress distribution make TPMS advantageous for lightweight load-bearing, energy absorption, and catalysis applications [13,20]. Furthermore, the topology of TPMS provides a simple means of tuning their mechanical and transport behavior. By adjusting the iso-surface constant (C), which sets the surface offset in the TPMS equation, we can modify stiffness, permeability, and relative density through a single parameter. Consequently, TPMS have transitioned from purely mathematical constructs into versatile design foundations for multifunctional metamaterials fabricated via AM. The following section discusses the research on some of the widely explored TPMS structures.

### 2.1. Primitive

Primitive or P structure is the first TPMS designed by H.A. Schwarz in 1865 [21]. Guo et al. proposed an optimized Schwarz primitive lattice structure to achieve superior specific energy absorption in the composites [22]. A shape parameter was introduced by redefining the shell opening diameter to obtain the optimized P-lattice. They achieved good surface quality using a finer laser beam, finer SS316L particles (5–25 μm), and a smaller layer thickness. The compression test demonstrated that the modified P-lattices outperformed the original P-lattices in terms of compressive strength and specific energy absorption (SEA). For battery temperature management systems, Cheung and his group created copper-plated polymer TPMS (Schwarz primitive) lattices. They achieved a 216% increase in thermal conductivity while maintaining polymer-like elasticity by adjusting relative density and Cu thickness, which allowed them to decouple pressure drop, thermal conductivity, and mechanical compliance, providing multipurpose cold plate performance [23]. Maskery and colleagues used PA2200 polymer to study primitive TPMS, diamond, and gyroid lattices, which were SLS-fabricated. While other TPMS structures, such as gyroid and diamond, exhibited bending-dominated behavior with longer plastic plateaus, the primitive structure showed a 100% greater elastic modulus due to stretching-dominated deformation, illustrating the impact of cell shape on stiffness and failure mode [24]. Fabricated via the LPBF technique, Mulhi et al. printed primitive TPMS structures using 17–4 PH stainless steel, showing improved heat transfer performance due to its topology. Micro-CT analysis shows that larger cells have thicker walls and larger pores, but the surface area to volume ratio decreases. However, higher porosity results in thinner walls and larger pores. Due to shrinkage, manufactured lattices exhibited dimensional variations from CAD models, with minimum printable wall and pore diameters of 152 μm and 317 μm, respectively [25]. Jagadeesh and Duraiselvam investigated the compressive performance of primitive TPMS lattices with graded cell sizes, manufactured through SLM of SS316L stainless steel. Compared to its uniform counterparts, the graded structure demonstrated a greater quasi-elastic modulus (3.12 GPa) and noticeable layer-wise deformation. Despite somewhat reduced energy absorption, the graded design maintained comparable efficiency to the 4 mm uniform lattice, indicating its potential for load-bearing applications in the biomedical sector [26].

**Figure 1:**
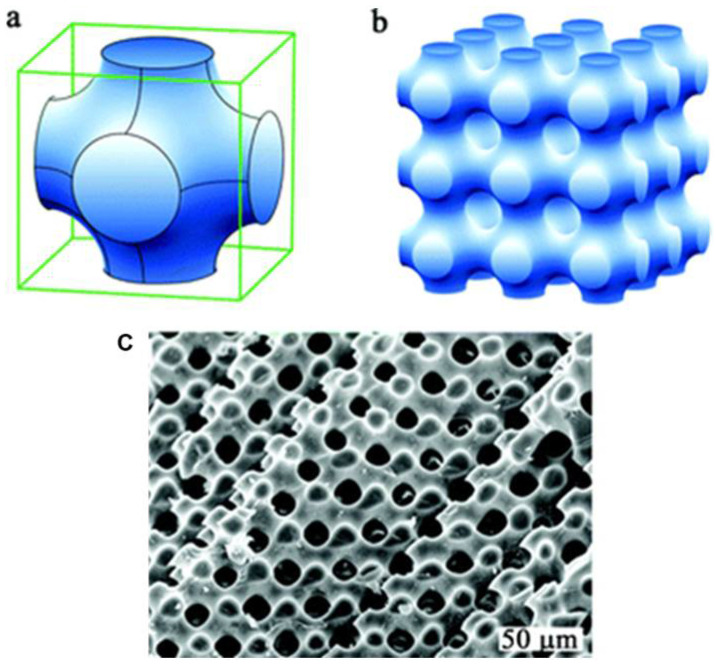
(**a**) CAD model of Schwarz primitive unit cell, (**b**) periodic structure of P surface, (**c**) cross-section through a sea urchin skeletal plate showing resemblance to the P surface. Reproduced with permission from [29].

For pediatric fixation devices based on TPMS-based Ti-6Al-4V, Dehaghani and others presented a design approach that enhances stiffness, strength, osteointegration, and manufacturability. The results indicated that primitive lattices had the largest pore size, the lowest surface area, the highest Gaussian curvature, and the best strength-to-stiffness ratio among the six types of TPMS. Therefore, they are suitable for reducing stress shielding and facilitating easier implant removal [27]. Almomani and Mourad studied the fracture toughness K_IC_ of the P-lattice using single-edge notched bend (SENB) specimens. The fracture toughness was found to be linearly related to linear density and the square root of the unit cell size [28]. Figure 1 presents a primitive TPMS surface, whose design is inspired by natural structures.

### 2.2. Diamond

**Figure 2:**
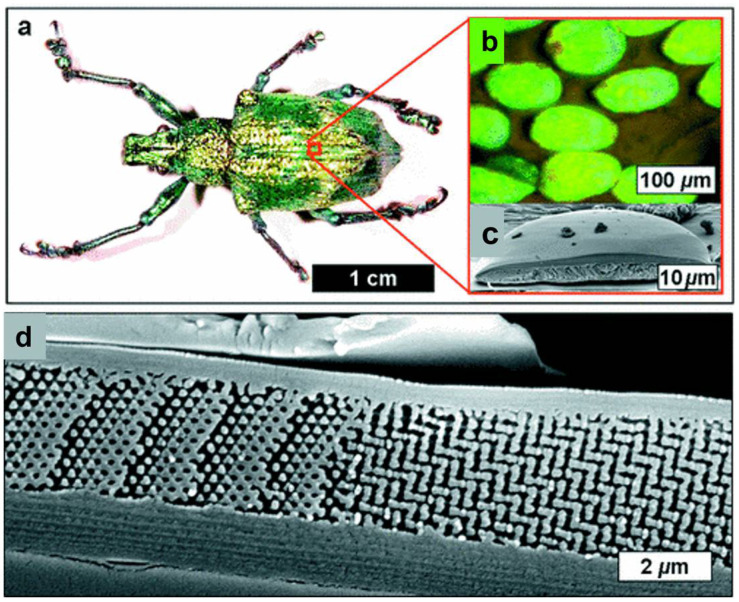
Diamond structure found in weevil exoskeletons. (**a**) photograph of the weevil *L. augustus*. (**b**) optical micrograph of individual scales attached to the exoskeleton of *L. augustus* under white-light illumination. (**c**) cross-sectional SEM image of a single scale. (**d**) SEM image of a region of a scale. Reproduced with permission from [37].

German mathematician Schwarz introduced the diamond surface in 1865. Shah et al. demonstrated that diamond structures exhibit the highest resistance to buckling [30]. The impact of column height on critical buckling load was also examined, revealing that the diamond design shows a uniform decrease with height. Zhang and the group fabricated several TPMS structures using SLM with 316L stainless steel. After comparing their energy absorption capabilities with BCC structures, diamond-type sheet structures were found to perform the best [31]. Spece, DeSantis, and Kurtz performed numerical homogenization to visualize the relationship between the mechanical and structural characteristics of gyroid and diamond structures [32]. Using SLM, Laskowska and colleagues investigated the compressive behavior and energy absorption of diamond TPMS cylindrical constructions made from 316L stainless steel. With greater stability during compression than gyroid structures, bending-dominated designs (n_radial = 1) demonstrated superior energy absorption but less stiffness and strength than stretch-dominated ones [33]. Giorleo et al. evaluated the acoustic absorption of material-jet-printed diamond and gyroid TPMS structures. Due to its increased tortuosity and flow resistance, diamond TPMS with thin walls (0.5 mm) and a low aspect ratio (AR = 0.5) outperforms gyroid in low-frequency sound attenuation, achieving peak absorption (α = 1) at 1250 Hz [34]. Felix and others studied the atomic and macroscale compression behavior of diamond schwarzite (D8bal) structures. The structure’s TPMS topology is significant in controlling mechanical behavior, evidenced by the scale-independent, bending-dominated, layer-by-layer deformation observed in both PLA 3D-printed models and atomistic Molecular Dynamics (MD) simulations [35]. Yeranee and the group performed a thermo-fluid-structural investigation of diamond-type TPMS structures for gas turbine blade trailing edge cooling. The diamond TPMS demonstrated its potential for high-efficiency cooling under extreme thermal loads by achieving a 145.3% increase in heat transfer, a 19.9% decrease in external surface temperature, and significant reductions in thermal displacement (29.3%) and Von Mises stress (28.8%) compared to conventional pin fin arrays [36]. Figure 2 illustrates the diamond TPMS architecture observed in the exoskeletons of weevils.

### 2.3. Gyroid

The gyroid structure was first discovered by the renowned NASA scientist Alan Schoen in 1970 [11]. It is an embedded surface associated with diamond and primitive [38]. The gyroid structures, traditionally utilized for energy absorption and chemical catalysis, are increasingly employed as biomorphic scaffolds for bone repair and regeneration [39]. Stainless steel powder was used to manufacture the structures, and their mechanical behavior was analyzed across two different wall thicknesses. Peng and colleagues presented a graded gyroid sandwich panel inspired by nature, demonstrating improved resistance to blast loading [40]. When subjected to impulsive loads, they conducted numerical investigations into the static and dynamic behaviors of gyroid lattice and cellular composite structures. Fused deposition modeling (FDM) often utilizes the gyroid infill pattern in printed objects. This pattern reduces printing time and material while providing the necessary strength and resistance in the printed part. Gyroid infill also offers nearly isotropic properties due to its cubic symmetry. Prussack and the group compared 3D-printed gyroid and diamond TPMS heaters using volumetric heating and forced convection. Compared to conventional rod bundles, TPMS structures exhibited 8–10 times higher Nusselt numbers (Nu) and up to 275% greater power density. The gyroid had the maximum heat transmission and friction factor, confirming TPMS as a promising core geometry for nuclear reactors [41]. Shaikh et al. used DLP-based additive manufacturing to study functionally graded gyroid TPMS structures for non-pneumatic tires. According to the results, graded designs (1.0–2.0 mm thick) were more effective at distributing loads and exhibiting smoother deformation than uniform designs. They also showed up to 53% more stiffness and 58% less local deformation. These designs are therefore ideal for applications involving harsh terrain [42]. Al-Ketan, Lee, and Al-Rub used implicit functions to create stochastic gyroid-based sheet lattices fabricated via laser powder bed fusion with SS316L. They discovered that near-isotropic behavior was guaranteed when nine or more control points were present. At low relative density, periodic gyroids performed better mechanically than stochastic lattices, which displayed a more uniform stress distribution. Due to better support and fewer flaws, the stochastic structures only showed improved qualities above 19–20% relative density [43].

**Figure 3:**
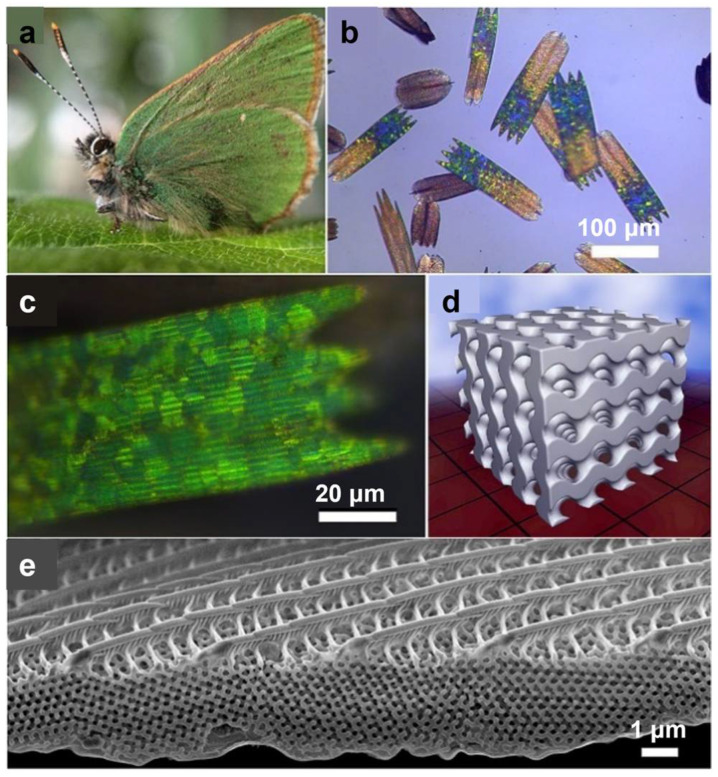
Gyroid structure found in a butterfly wing. (**a**) picture of *Callophrys rubi*, also known as the green hairstreak. (**b**) optical microscope image of both colored and brown cover scales, (**c**) magnified view of the wing scale, (**d**) CAD model of gyroid, (**e**) scanning electron microscopy (SEM) image of the ribbed upper surface. Reproduced with permission from [48].

Vafaeefar et al. evaluated the energy absorption under compression of gyroid, dual-lattice, and spinodoid structures manufactured using stereolithography (SLA). Due to its bending-dominated behavior, the gyroid exhibited greater efficiency and delayed densification, while the dual-lattice demonstrated the most significant energy absorption. These findings imply that the gyroid would be suitable for impact reduction applications [44]. Abueidda and the team investigated the mechanical properties of gyroid structures fabricated using the selective laser sintering (SLS) method [45]. The gyroid structures uniaxial modulus, compressive strength, and energy absorption are compared with those of the I-graph-Wrapped Package (IWP), Neovius, and primitive structures. Gyroid structures exhibited relatively good mechanical properties compared to other TPMS cellular structures. Simsek and others studied the vibration characteristics of the double gyroid structures [46]. Auxetic structures exhibit a negative Poisson’s ratio. They do not fail catastrophically after the first crack, and the structure can endure more load while maintaining its integrity. Nath and Nilufar showed that auxetic structures have a higher energy absorption capacity due to their failure characteristics [47]. Figure 3 depicts the gyroid TPMS structure within butterfly wings.

Viswanath et al. suggested a 3D CNN-based surrogate model for optimizing gyroid TPMS unit cell topology. The model effectively anticipated optimal structures for specified volume fractions and filter radii using voxelized inputs and homogenization-based topology optimization, significantly reducing the time needed to develop metamaterials with desired mechanical properties [49]. Huffman and Youssef conducted a comprehensive investigation into the mechanical performance of gyroid, Schwarz diamond, and Schwarz primitive TPMS structures fabricated via VPP with varying levels of geometrically designed porosity. Their findings demonstrated that increasing porosity led to reduced stiffness and peak stress while promoting extended plateau regions, thereby enhancing deformability under both quasi-static and low-velocity impact conditions. Among the geometries examined, gyroid structures exhibited superior energy absorption efficiency. Additionally, a dual-hybrid strategy combining geometrical porosity with glass microballoon reinforcement further improved the impact mitigation capability of the gyroid architecture [50].

### 2.4. Neovius

The Neovius surface was discovered by Finnish mathematician E.R. Neovius in 1883 [51]. The mechanical properties of the Neovius micro-lattice structure were investigated by Abueidda and colleagues [52]. This structure can be coated with a ceramic (alumina) layer to enhance buckling stability. Hur et al. explored the phononic properties of the Neovius structure for better thermal insulation, reduced sound noise, and conversion of wasted heat into electricity [53]. Khan and Al-Rub studied the viscoelastic behavior of Neovius foams under different loading conditions [54]. A study by Khan and others investigates the compressive behavior of Neovius TPMS structures made with ABS material via fused deposition modeling (FDM). The results show that, regardless of relative density, Neovius lattices have greater yield strength and initial peak strength than IWP lattices [55]. AlMahri and the team assessed the quasi-static and dynamic compressive characteristics of two TPMS sheet-based structures (FRD and Neovius) manufactured with SLM in SS316L. It showed that Neovius performed better than FRD regarding specific energy absorption (SEA) and plateau stress, with greater values under dynamic loading due to improved strain hardening effects and strain-rate sensitivity [56]. Silva et al. conducted a numerical study on phononic bandgap formation across seven TPMS lattice types: primitive, Neovius, gyroid, IWP, diamond, FRD, and Fischer Koch S (FKS) at various volume ratios using finite element simulations. They found that the primitive and Neovius structures exhibited the widest complete band gaps (up to 0.483 kHz and 0.458 kHz, respectively) for volume ratios above 0.3, while the diamond structure consistently showed the narrowest bandgap widths and the highest central frequencies (up to 1.81 kHz). This study serves as a valuable database for designing TPMS-based metamaterials for acoustic and vibration isolation applications [57].

### 2.5. Schoen IWP

Alan Schoen first introduced the IWP surface in his famous NASA technical report [11]. This surface has eight openings located at the vertices of the cube. Baghous et al. conducted a numerical investigation on the impact of various loading conditions on the adequate yield strength of IWP structures [58]. IWP foam proves to be the ideal material for use as a damper when subjected to uniaxial and hydrostatic loading [59]. Several TPMS structures are named FRD, I2-Y, PMY, FKS, Lidinoid, Octo, Split P, and Crossed Layers of Parallel (CLP). The FKS scaffold may serve as a promising option for replacing the defective cortex in the tibial midshaft [60]. Using masked stereolithography (MSLA), Hassan and colleagues created uniform and graded Schoen IWP TPMS structures. They discovered that the graded design’s layer-by-layer collapse behavior led to improved deformation control and higher energy absorption. The result indicated that, compared to the Schoen primitive, the Schoen IWP structure exhibited enhanced mechanical performance, rendering it suitable for impact and lightweight applications [61]. Xu and others presented an innovative method for creating TPMS-like shell lattices, combining intentionally designed periodic boundaries with implicit surface modeling. Through compression tests and homogenization simulations, it was confirmed that their IWP-like structures, when compared to various topologies, had greater stiffness and broader mechanical property ranges than conventional Schoen IWP structures [62]. Kumar, Ramkumar, and Balani utilized ABS resin and the SLA technique to design and fabricate TPMS-based scaffolds (IWP, Neovius, primitive, and FRD), to investigate the impact of porosity and unit cell size on mechanical performance. The results showed that IWP possesses the highest compressive strength (39.8 MPa) and Young’s modulus (1.09 GPa). It also demonstrated that the TPMS lattices exhibit superior deformation behavior and a uniform stress distribution compared to single-unit cells [63]. Surface area, volume fraction, and hydraulic diameter are important geometric indices of TPMS structures made by additive manufacturing. He et al. investigated the relationship between these indices and design parameters. In contrast to conventional methods, a new parallelogram patch method for calculating area is presented, with an inaccuracy of less than 2.5%. Regression equations are developed to predict geometric performance. Schoen IWP, FKS, and Schwarz primitive are the structures with the most favorable geometric properties, providing valuable insights for effective TPMS lattice construction [64].

### 2.6. Mathematical Representation of TPMS Structures

To provide a unified perspective on the geometries discussed above, the most commonly used TPMSs can be expressed through implicit level-set functions. These mathematical formulations define the periodicity, symmetry, and zero-mean-curvature characteristics of each TPMS. They also enable direct precise control of porosity, wall thickness, and relative density through the adjustable design parameters. In these mathematical expressions, the spatial frequencies $\omega_{x}$, $\omega_{y}$, and $\omega_{z}$ defines the periodicity of the surface along the x, y, and z directions, respectively. The unit-cell size in each direction is determined by

$$L_{i}=2\pi /\omega_{i}$$

The level-set parameter C shifts the surface to vary the final porosity and sheet/strut thickness. These mathematical definitions are widely used in computational modeling, CAD generation, and additive manufacturing workflows to ensure geometric accuracy and reproducibility. Table 1 summarizes the implicit functions of the most widely studied TPMS structures, along with the associated design parameters used in computational modeling and CAD construction.

Images are produced using MSLattice 1.0 software, where C is the level-set parameter controlling porosity, wall thickness, and relative density [65]. Here, x, y, z denote Cartesian coordinates; ω_x,_ ω_y_, ω_z_ represent spatial frequencies (rad·mm^−1^), which determine the unit cell size, $L_{i}=\frac{2\pi }{\omega_{i}}$. Variations in ω and C allow the generation of TPMS structures with uniform or graded relative density.

### 2.7. Summary of Geometric Features, Manufacturing Methods and Mechanical Performance

To support the comparison of results discussed above, Table 2 summarizes a selection of representative experimental studies. It includes the fabrication of TPMS lattices from metals, polymers, and hybrid materials through SLM/LPBF, SLA, FFF, and related AM methods. For each study, the table provides TPMS topology, relative-density range, key mechanical properties, and energy-absorption metrics, including specific energy absorption (SEA), total absorbed energy, and densification strain. This comparative review also highlights the characteristic deformation of sheet-based and skeletal TPMS structures. It also provides reference values to support the design of energy-absorbing applications.

The information discussed in Table 2 highlights the trends that consistently appear across different materials and TPMS designs. Increasing relative density generally raises plateau stress and the total absorbed energy. However, the rate of improvement decreases once the deformation mode shifts from bending-dominated to stretching-dominated behavior. Sheet-based architectures such as Diamond and Gyroid typically provide higher energy-absorption capacity than strut-based layouts at comparable density. This improvement is attributed to smoother stress redistribution and the lack of nodal stress concentrations. Post-processing, such as heat treatment of Al-Si10-Mg or reinforcement of polymeric lattices, can alter the collapse behavior and expand the effective plateau region. Graded TPMS structures offer additional flexibility in design. This allows stiffness and deformation response to be tuned through spatial variations in cell morphology. Collectively, these comparisons clarify how topology, density, and processing route influence the mechanical and energy-absorption response of TPMS lattices. These comparisons show how topology, relative density, and processing shape the mechanical and energy absorption behavior of TPMS lattices.

## 3. Additive Manufacturing Methods for TPMS

AM made it possible to fabricate complicated shapes efficiently. This section provides a brief overview of several AM processes that are widely used to manufacture TPMS structures.

### 3.1. Material Extrusion

Material extrusion (MEX) involves pushing a spool of material, typically thermoplastic polymer, through a heated nozzle at a steady pressure in a continuous stream. The desired materials are selectively deposited, layer-by-layer, according to the design, to create 3D parts. It is also called fused filament fabrication (FFF). The Stratasys company first commercialized this technology in 1990, adopting the proprietary name fused deposition modeling (FDM) from there [73]. FDM printers are the prevailing choice for polymer printing. A wide range of materials can be used for this FFF process. The most commonly used thermoplastic materials include acrylonitrile butadiene styrene (ABS), acrylonitrile styrene acrylate (ASA), polycarbonate, polyetherimide, polylactic acid (PLA) high-impact polystyrene (HIPS), thermoplastic polyurethane (TPU), aliphatic polyamides (PA, Nylon), and high-performance plastics such as polyether ether ketone (PEEK), and polyetherimide (PEI) [74,75,76,77]. The FDM technique is widely utilized by various sectors, academia, and consumers for manufacturing prototypes and functional components with commodity and engineering plastics due to its ease of use, reliability, and affordability [78,79,80,81]. These printers are now commonly used to make the TPMS structures. Spece, DeSantis, and Kurtz printed gyroid and diamond structures using PEEK filament and analyzed their structural properties for two different build orientations. Their results showed that the model accurately predicted z-direction properties. At the same time, it was less accurate in the x and y direction due to the weak interlayer bonding and print path irregularities [32]. Hashimi et al. used the material extrusion process to print gyroid, primitive TPMS, and cubic scaffolds. They overcame challenges in print optimization, pore formation, and material parameters. Their findings proved that melt extrusion is a reliable method for producing biomimetic TPMS scaffolds for bone generation [82]. Sabahi and others employed the material extrusion process to print density graded TPMS PEEK scaffolds, integrating gyroid, diamond, and Schwartz primitive structures. Their results indicated that gyroid and diamond closely resemble trabecular bones, making them potential candidates for bone generation [83]. Wang and colleagues printed six different TPMS structures using the fused filament fabrication (FFF) technique and performed quasi-static compression tests. They evaluated compression properties such as total energy absorption (TEA), SEA, mean crushing force, crush force efficiency (CFE), and the fluctuation coefficients [84]. Observations suggest that Schoen IWP specimens underwent stable deformation, characterized by layer-by-layer lobe formation. The Schwarz primitive structure exhibited oscillatory behavior due to visible buckling. The PW hybrid structure displayed the highest specific energy absorption compared to the other configurations. Desole, Gisario, and Barletta analyzed the behavior of three types of cellular structures (strut-based, surface-based TPMS, and spinodal) using PLA to construct these structures. The results demonstrated that TPMS structures are the most durable and resistant to cyclic impact loads, laying the groundwork for developing prototypes with extended life cycles, increased strength, and improved durability [85].

### 3.2. Powder Bed Fusion

PBF methods utilize an electron beam or laser to melt and fuse the material powder. A recoater creates a uniform layer, allowing the subsequent layer to form. Depending on the type of material and heat source employed, the PBF process can be categorized into numerous variations. Commonly, PBF methods include Direct Metal Laser Sintering (DMLS), Electron Beam Melting (EBM), Selective Heat Sintering (SHS), Selective Laser Melting (SLM), Metal Laser Melting (MLM), and Selective Laser Sintering (SLS). Each variant has its advantages and disadvantages, with the application evaluated based on suitability [86]. SLS, often called laser beam PBF (PBF-LB), sinters powdered polymer materials, such as nylon or PEKK, to create complex three-dimensional objects. Each layer is formed by depositing powder materials sintered with a laser beam to create a solid layer. The final product is achieved by building up layers one at a time. SLS with enhanced microscale resolution is known as micro-selective laser sintering (µSLS). This method can achieve feature sizes with a resolution of less than 5 μm [87]. This SLS technique can be used in the microscale fabrication of sensors, actuators, etc. Unlike SLS, which sinters the powder, SLM completely melts it using a laser. SLM is primarily applied to metal powders such as aluminum alloys, titanium and its alloys, and stainless-steel alloys. Like SLM, EBM employs an electron gun instead of a laser, which is referred to as the PBF-EB method. The EBM build chamber operates under a vacuum rather than an inert atmosphere. Extracting powder from high-density porous structures can pose challenges. Khrapov et al. investigated ss techniques for removing trapped powder from sheet-based porous structures produced via electron beam powder bed fusion [88]. Qu, Ding, and Song manufactured TPMS structures using fine SS316L powders in a micro LPBF machine [89]. The results indicated that the TPMS thin-walled structure (TWS) enhances thermal performance. Abueidda and colleagues utilized SLS technology to fabricate three TPMS cellular materials (CM) and examined their linear and non-linear mechanical responses. Neovius-CM and IWP-CM demonstrated greater stiffness and strength compared to the primitive-CM [90]. Alsalla, Hao, and Smith fabricated gyroid lattices using the SLM technique and investigated the impact of building directions on fracture toughness and tensile strength. Results showed that properties of the samples built vertically exhibited greater strength than those constructed horizontally [91]. Hussain et al. studied the fabrication of gyroid and primitive lattices using the LPBF with varying process parameters. Their finding emphasized the significance of process parameters and geometry in optimizing LPBF printed TMPS structures to improve material characteristics and surface quality [92]. Araya and others studied the mechanical properties of Ti-6Al-4V lattice structures fabricated through LPBF, focusing on TPMS gyroid and Stochastic Voronoi designs. The results indicated that the mechanical properties of both lattices are comparable to those of human bones, suggesting significant potential for orthopedic applications [93]. Tilton and the group explored the fatigue performance of LPBF Ti-6Al-4V scaffolds designed with TPMS geometries for orthopedic applications. Their results showed that Schoen-IWP exhibited longer fatigue life than the primitive scaffolds at similar porosity and amplitude. However, the compressive mechanical properties of the primitive scaffolds are more comparable to trabecular bone [94]. Yang, Tang, and Tang investigated the printability and compression behavior of Ni-based alloy ABD-900 AM TPMS structures fabricated via SLM. They analyzed six TPMS designs, all demonstrating high fabrication quality with minor deviations. Among these structures, diamonds were the strongest, followed by Split-P, primitive, gyroid, IWP, and Lidinoid [95].

**Figure 4:**
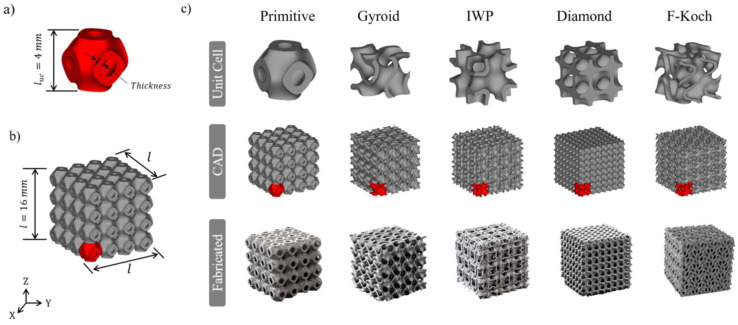
(**a**) Unit cell of Primitive. (**b**) Primitive lattice. (**c**) CAD of unit cells, lattice structures, and fabricated samples of Primitive, Gyroid, IWP, Diamond, and Fisher–Koch lattice structures. Reproduced under the terms of the CC BY 4.0 License [96].

AlMahri et al. [96] fabricated five different types of TPMS lattices using the LPBF process, enabling a direct comparison between the CADs and the printed specimens. Figure 4 shows a comparison that reveals common deviations between the intended design and the manufactured TPMS structures.

The printed TPMS specimens generally preserved the intended topology; however, systematic deviations from the CADs were observed. Wall (sheet) thicknesses measured from micro-CT and SEM were consistently smaller than the designed values. For example, the primitive unit cell was designed with a wall thickness of 770 μm, but the actual measured thickness was 723 μm. These reductions in local thickness translated to lower actual relative densities: the primitive lattice designed at 30% relative density had an actual measured density of 28%. Deviations increased with higher design relative density. The primitive lattice had deviations of 6.2% at 30% and 8.8% at 50%. The principal causes are process-related. Insufficient local energy input leads to a lack of fusion and undersized sheets. Suboptimal hatch spacing and manufacturing defects such as voids and partially melted particles can increase surface roughness and reduce the effective volume. These observations highlight the importance of process-parameter control when designing TPMS lattices to achieve target densities and mechanical performance.

### 3.3. Vat Photopolymerization

#### 3.3.1. Stereolithography (SLA)

SLA is the Vat Photopolymerization (VPP) process, developed by Charles Hull in the mid-1980s. It is also known as vector scan or point-wise printing. Currently, it is widely used across various industries. This process requires a resin container filled with photopolymers and a light source. Photopolymers react with ultraviolet (UV) radiation, though some resins also respond to visible light. Vat photopolymers comprise photoinitiators, reactive diluents, flexibilizers, stabilizers, and liquid monomers. Upon UV irradiation, the photoinitiator undergoes a chemical change and reacts with a monomer, which then interacts with other monomers, creating a chain reaction. Depending on the position of the UV laser beam, there are two types of SLA printing methods: the top-down approach and the bottom-up approach. In the top-down printing method, a UV laser beam is directed downward and follows the design path on the surface of the resin. This action cures the resin and forms a solid printing layer at the desired depth from the resin surface. After printing the first layer, the printing base is lowered, and the printing of the next layer begins. Careful adjustment in printing orientation can prevent the entrapment of uncured resin-specific designs. In the bottom-up approach, a UV laser beam is projected upward. The bottom of the resin tank features a window that allows the laser to pass through. The distance between the printing base and the resin tank determines the layer height. UV light cures the resin beneath the printing base, and after each layer is printed, the printing base is raised by the predetermined layer height. The printing size in this process is constrained by the adhesion of the base layer to the printing base. Santoliquido et al. compared both “top-down” and “bottom-up” approaches for additive manufacturing of ceramic components [97]. Gabrieli and the team fabricated Schwarz and gyroid-structured scaffolds using stereolithography technology to mimic the properties of cancellous bone. The scaffolds exhibited 40–85% porosity, comparable to spongy bone, although the gyroid had larger pores [98]. Liang and colleagues examined the quasi-static mechanical properties of the resin-based homogenous and gradient TPMS structures fabricated via SLA. The experimental and numerical results showed that the gradient TPMS structures exhibited superior energy absorption compared to the homogeneous TPMS structures. They also showed that single-direction gradient TPMS structures have the best mechanical properties when aligned with the force direction, as their layer-by-layer deformation enhances energy absorption [99].

#### 3.3.2. Digital Light Processing (DLP)

DLP is similar to SLA. However, in this process, the photo-resin is cured using a DLP projector instead of UV radiation [100]. Consequently, DLP offers a faster printing speed than SLA. Due to mask projection, there is a trade-off between resolution and the size of the solidified cross-section. Modern digital micromirror devices (DMDs) have a resolution of 1920 × 1080, and the maximum part size at 50 μm resolution is 96 × 54 mm.

A drawback of VPP processes is that printing materials are restricted to acrylates, vinyl ethers, and epoxies. Epoxies continue to cure even after the light source is turned off; however, acrylics only cure to about 75 to 80% because curing stops as soon as the light source is switched off [101]. These materials do not possess the impact strength and durability of high-quality injection-molded thermoplastics. Additionally, their mechanical properties deteriorate over time [2].

**Figure 5:**
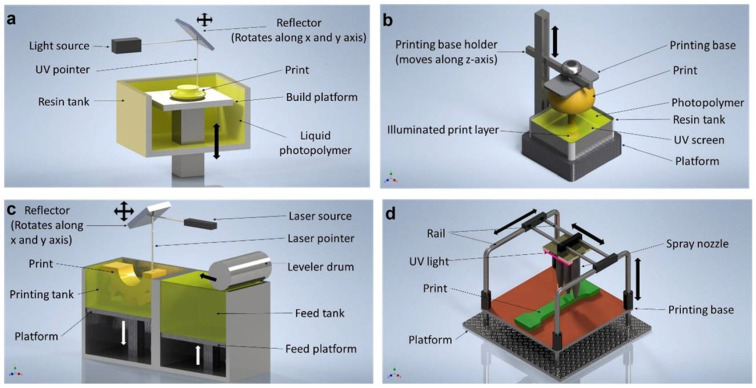
AM techniques commonly used in manufacturing TPMS structures. (**a**) stereolithography (SLA), (**b**) digital light processing (DLP), (**c**) selective laser sintering (SLS), (**d**) material jetting (MJT). Reproduced under the terms of the CC BY 4.0 License [105].

Roohani et al. presented a Liquid Crystal Display (LCD) technique for manufacturing highly porous bioceramic TPMS scaffolds using VPP. Their findings highlighted that LCD could serve as a robust alternative to conventional SLA for manufacturing optimized bone scaffolds and patient-specific implants [102]. Chen and others fabricated biodegradable Zn-1Mg scaffolds with TPMS by combining VPP and casting for orthopedic implants. The results indicated that these scaffolds exhibited superior mechanical strength, stability, biocompatibility, and antibacterial properties, making them a promising candidate for orthopedic implants [103]. Hua and the group studied the bioactivity and mechanical strength enhancement of hydroxyapatite (HA) bioceramic using TPMS structures fabricated by DLP and incorporating akermanite (Ca_2_MgSi_2_O_7_). The result demonstrated that the scaffolds hold potential for repairing the cancellous bone defects [104]. Figure 5 exhibits different AM methods commonly used for polymer TPMS manufacturing.

### 3.4. Material Jetting

Material jetting is one of the fastest and most accurate AM processes. It follows a procedure similar to that of a two-dimensional inkjet printer [106,107]. Thermoset polymers are typically used for these AM processes, as they are available in liquid form. However, a variety of materials such as ABS, rubber, and fully transparent materials can also be employed in this process [73]. It employs either a continuous method or a drop-on-demand (DOD) technique to deposit material onto a build platform. This material is placed onto the build surface, where it solidifies, layer-by-layer, to form the model. Solid materials must be heated to a liquid state at room temperature to enable jetting. In the case of high-viscosity fluids, the viscosity of the material needs to be reduced to facilitate jetting. The most common methods include using heat, solvents, or other low-viscosity components in the fluid. Polyjet printing technology is employed to print several TPMS structures. Afsar et al. investigated the mechanical properties and deformation mechanisms of TPMS-based scaffolds with linearly graded porosity. Their experimental and numerical results were closely aligned, which confirmed the effectiveness of graded porosity in optimizing scaffold performance [67]. Multiple nozzles are used in this process, speeding up the printing method. Support material can also be injected through the support nozzles. Sathishkumar and colleagues first examined the compressive failure of Schoen’s OCTO and FRD-based lattice structures [108]. Polyjet printing was used to manufacture those lattice structures. Schoen’s OCTO demonstrated superior mechanical strength, withstanding 26.9% higher stress than FRD structures of the same thickness. Shilova discussed the possibility of TPMS formation in sol–gel-derived thin films [109]. Table 3 summarizes the different TPMS structures fabricated using various AM techniques and a wide range of materials.

### 3.5. Design for Manufacturability (DfM) Considerations for TPMS Printing

TPMS structures have continuous, highly intricate geometries, which make them particularly sensitive to manufacturing limitations. Unlike strut-based lattices, TPMS structures are defined by curvature continuity; thus, even small dimensional deviations can alter relative density, surface curvature, and structural performance. The manufacturability of TPMS lattices depends on the energy input, layer thickness, and material rheology characteristic of each AM process. Furthermore, their bicontinuous nature creates challenges in powder removal, resin drainage, and support accessibility. Table 4 summarizes representative designs for manufacturability (DfM) parameters and process-specific guidelines for TPMS fabrication across major AM techniques.

## 4. Process Parameters and Post-Processing Considerations

The fabrication quality of TPMS structures is highly sensitive to the control of process parameters across VPP, LPBF, and MEX-based AM systems. Since TPMS structures contain thin walls, continuously curved surfaces, and interconnected porosity, small variations in processing conditions can lead to significant deviations in geometric accuracy, mechanical performance, and reproducibility. To support practical implementation, this section combines evidence from TPMS studies and established AM process-parameter assessments.

**Figure 6:**
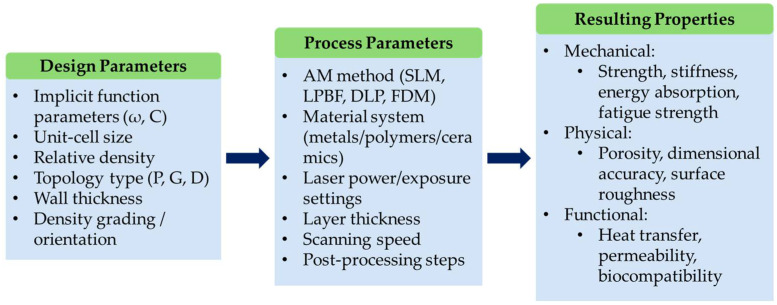
Relationship between TPMS design parameters, AM process parameters, and resulting properties.

The flowchart presented in Figure 6 illustrates the relationship between TPMS lattice design parameters, additive manufacturing (AM) process parameters, and the resulting structural and functional properties. At the design stage, geometry is defined using implicit mathematical functions, where variables such as spatial frequency (ω), level-set constant (C), and unit-cell size directly influence the sheet thickness and relative density of the structure. Additional geometric features, such as density grading, orientation, and the number of unit cells, allow further customization of mechanical and functional responses. These design choices not only define the overall morphology but also determine manufacturability and sensitivity to process parameters.

Process parameters such as laser power, scanning speed, layer thickness, hatch spacing, build orientation, and post-processing (e.g., heat treatment, surface finishing) directly affect how the design is produced during the additive manufacturing process. Variations in these parameters can lead to deviations in relative density, wall thickness, porosity distribution, and surface roughness. They govern how closely the printed structure will resemble the original design. As both design and process parameters interact, they determine the final properties of the printed TPMS structures. These resulting properties include mechanical performance, dimensional accuracy, porosity, surface morphology, and overall structural integrity.

### 4.1. Dimensional Accuracy

#### 4.1.1. Layer Thickness

Layer thickness directly influences the degree to which a printed TPMS captures its mathematically defined curvature. For mSLA printing, Ramírez Rodríguez et al. [142] demonstrated that decreasing layer thickness and applying surface offsets significantly reduced surface roughness in Gyroid TPMS lattices. These trends are consistent with classical stereolithography photopolymerization theory, where smaller layer heights reduce staircase effects and improve vertical resolution [198]. In LPBF, thin layers stabilize melt-pool geometry, reduce defect formation, and improve dimensional accuracy, commonly observed in metallic AM process reviews [6,199,200].

#### 4.1.2. Energy Input (Laser Power, Scanning Speed, UV Exposure)

Optimizing energy density is essential for achieving accurate TPMS geometries. In VPP, curing depth follows Jacobs’ exponential attenuation model, where increasing exposure energy enhances polymer conversion and reduces feature loss [198]. For LPBF, energy density (a function of laser power, scan speed, and hatch spacing) governs melt-pool penetration and continuity. Higher energy densities reduce the lack of fusion defects, improve feature definition, and enhance geometric accuracy in thin-walled structures [6,199,200].

#### 4.1.3. Hatch/Scan Spacing

Hatch spacing determines melt-pool overlap. Smaller hatch spacing increases bonding between adjacent tracks, reduces porosity, and improves surface continuity in curved TPMS sheets. Foundational LPBF studies show that insufficient overlap leads to discontinuities and dimensional error in architected structures [6,199,200].

### 4.2. Mechanical Performance

#### 4.2.1. Structure Parameters and Wall Quality

For SLM-fabricated 316L Gyroid TPMS structures, Szatkiewicz and Laskowska [115] reported that wall thickness and unit cell configuration strongly influence stiffness, deformation modes, and energy absorption. Their findings align with LPBF process microstructure mechanics correlations described in the metallic AM literature [6,200], where melt-pool stability and cooling rate dictate mechanical homogeneity.

#### 4.2.2. Curing Depth and Resin Exposure (VPP)

Photopolymer network formation depends on curing depth, exposure energy, and resin formulation. Jacobs’ original stereolithography model [198] and subsequent ceramic SLA studies [201] show that deeper curing increases the degree of conversion, reduces internal weakness, and enhances mechanical robustness in printed lattice structures. These principles directly translate to VPP TPMS fabrication.

#### 4.2.3. Material Feedstock and Thermal Conditions (FFF/MEX)

Guo et al. [141] optimized extrusion temperature, printing speed, and cooling conditions for PLA/GO TPMS scaffolds and demonstrated improvements in porosity uniformity and compressive response. These observations are consistent with established MEX parameter studies, which show that extrusion temperature, raster pattern, and cooling rate substantially influence crystallinity and mechanical behavior [199,202].

### 4.3. Reproducibility

#### 4.3.1. Environmental Conditions

Reproducibility in polymer AM is affected by temperature and humidity, which alter resin viscosity (VPP) and affect cooling/solidification dynamics (FFF/MEX). Stable environmental control improves dimensional consistency and inter-batch uniformity [202].

#### 4.3.2. Post-Curing (VPP)

Post-curing increases polymer conversion, reduces residual monomers, and improves mechanical stability. Classical photopolymerization research demonstrates that extended UV exposure enhances crosslink density and reduces variability [198,201].

#### 4.3.3. Powder Characteristics and Reuse Fraction (LPBF)

Powder morphology, particle size distribution, and refresh ratios influence melt-pool stability and porosity formation in LPBF systems. Foundational reviews highlight the need for maintaining controlled powder reuse ratios to minimize variability in dimensional accuracy and mechanical properties [6,199,200].

### 4.4. Post-Processing Effects on TPMS Structures

**Figure 7:**
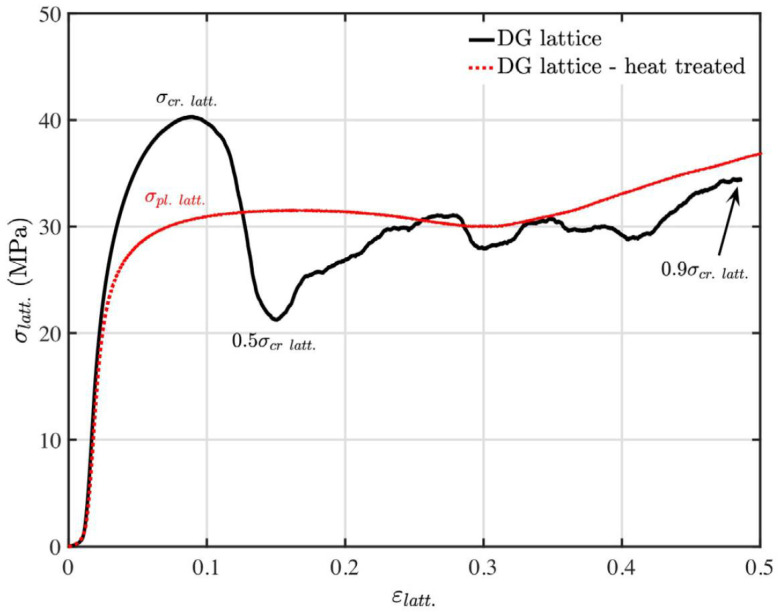
Effect of heat treatment on the stress–strain curve of double gyroid lattice structure. Reproduced under the terms of the CC BY 4.0 License [20].

Conventional manufacturing typically involves several post-processing steps, including support removal, material and surface enhancements, heat treatment, and blasting. Similarly, additively manufactured parts often need post-processing and finishing to meet the desired dimensions and properties. These operations may include heat treatments, machining, and procedures that extend beyond simple media blasting, sanding, or coating. Yan and group performed heat treatment of SLM-made Ti-6Al-4V gyroid and diamond structures at 680 °C for 4 h [66]. The as-built samples contained α′ martensite with a width of 100~300 nm. Post-processing with heat treatment, followed by sandblasting, transformed the fine α′ martensitic structure into a mixture of α and β. The width of the α grains ranged from 500 to 800 nm, which is larger than that of the α′ martensitic structure. Maskery and colleagues explored the effect of heat treatment in double gyroid lattices made of Al-Si10-Mg [20]. The heat treatment improved the material’s ductility while decreasing its ultimate tensile strength, as shown in Figure 7.

### 4.5. Best Practice Guidelines for TPMS Fabrication

Table 5 presents the recommended best practices. These guidelines incorporate TPMS-specific characteristics together with well-established AM process–structure relationships. In doing so, they provide a framework for improving geometric accuracy, mechanical performance, and fabrication reproducibility.

## 5. Relative Density and Unit Cell Grading

**Figure 8:**
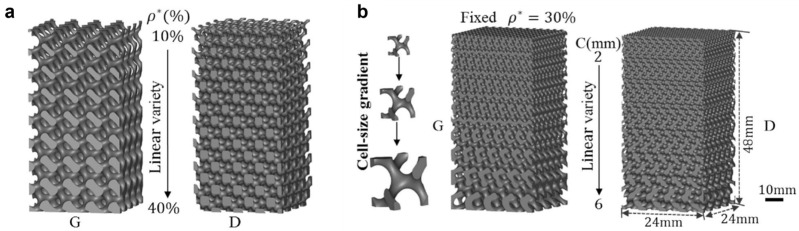
Designed models of gradient samples (**a**) in relative density and (**b**) in cell size. Reproduced under the terms of the CC BY-NC-ND License [203].

The geometries significantly influence the performance of TPMS cellular structures. The dimensions of the unit cell can be gradually varied in different directions. Liu et al. studied graded porous scaffolds generated from gyroid and diamond surfaces, shown in Figure 8 [203].

Additionally, cell size was varied while maintaining constant relative density. Scaffolds with smaller cells exhibited higher strength than those with larger cells. Yan and others reported similar results while investigating gyroid lattice structures built by the SLM process [204]. Both yield strength and Young’s modulus decreased with increasing unit cell size.

Cell type grading, or hybridization, can be achieved by combining two or more TPMS structures. Maskery and the team demonstrated a design approach for such heterogeneous TPMS porous structures and predicted the elastic modulus of the graded lattices [205]. Chen and colleagues studied hybrid cellular materials based on TPMS [206]. Previously, Yoo and Kim designed and manufactured multi-morphology cellular structures based on TPMS equations [207]. The influence of wall thickness grading and cell size grading has been analyzed and compared with uniform TPMS sandwich panels [208,209]. Zhao et al. investigated the mechanical properties of functionally graded sheet (FGS) TPMS structures and compared them with the uniform sheet (US) structures. The study revealed that US structures underwent abrupt shear band failures with a sharp drop in load-bearing capacity after the initial peak stress. In contrast, FGS structures deformed in a stable, layer-by-layer manner, resulting in improved structural integrity under compression. This behavior led to approximately 60% higher energy absorption in FGS structures, demonstrating their suitability for applications requiring controlled deformation and enhanced energy dissipation [65].

## 6. Applications for TPMS

Triply periodic minimal surfaces (TPMS) have a wide range of intriguing characteristics and applications. They have been utilized in the creation of new materials and structures and in modeling the structure of certain crystals and other materials. The following section presents a comprehensive overview of the applications of TPMS structures.

### 6.1. Mechanical Applications

A variety of factors, including cell topology, relative density, and wall thickness or strut diameter, influence the mechanical properties of TPMS structures. Jones et al. showed that material properties can be tailored by adjusting proportion, shape, and scale. Such parameters impact the properties of the materials like strength, stiffness, and ductility [210]. Altamimi et al. investigated 13 different strut-based lattice architectures with cubic symmetry derived from TPMS, Platonic, and Archimedean solids under uniaxial, shear, and hydrostatic loadings at various relative densities. This study provides valuable insights into topology-property relationships [211]. Araya and others investigated the mechanical properties of TPMS gyroid and Stochastic Voronoi designs manufactured by LPBF. They also conducted digital image correlation (DIC) and scanning electron microscopy (SEM) to analyze fabrication parameters, lattice morphology, and microstructural characteristics [93]. Shaikh and the group investigated how the mechanical characteristics of TPMS are affected by laser processing settings. According to their research, TPMS lattice mechanical performance can be optimized by functionally grading laser scanning techniques, allowing customized structural characteristics for cutting-edge applications [212]. Xu et al. investigated the scale effect on the mechanical properties of sheet-based TPMS lattice structures. Their investigation focused on the influence of wall thickness and unit cell size on deformation mechanisms, failure modes, stiffness, yield strength, first peak stress, and energy absorption [213]. BJ is one of the fastest and cost-effective techniques in AM. Xie and colleagues explored the fabrication, mechanical properties, and deformation behavior of uniform (U-GLS) and graded (G-GLS) gyroid lattice structures using the BJ technique with Ti6Al4V alloy. They analyzed the compressive properties and deformation behavior along with the FEA to simulate the mechanical response [169]. Yeo and others took a novel approach, focusing on TPMS-based Architectured Materials (HTAM). They created it by superimposing multiple triply periodic bicontinuous structures (TPBSs). Their novelty enabled designing the structures with enhanced mechanical stiffness and reduced weight that surpassed conventional single TPBS designs [214]. For the damping and mechanical properties, Wei and the team worked with Split P and BCC lattice structures, which were fabricated using LPBF. The compression and modal results showed that the TPMS structures outperformed the beam structures in mechanical performance. They also demonstrated that Split-P cells offer higher energy absorption and are suitable for lightweight applications. Additionally, their results showed that TPMS structures exhibited better damping performance [215]. Coarse-grained has a significant impact on the properties of additively manufactured materials. Hu et al. investigated the effect of grain coarsening on the mechanical properties of SS316L TPMS structures, which were fabricated by μLPBF. They showed that TPMS diamond structures with 25.7 μm grains showed improved energy absorption compared to fine-grained (20.2 μm) components [216]. Singh and others evaluated the fatigue performance of gyroid and IWP-based TPMS cellular materials, which were manufactured by hybrid casting and PBF methods. Results showed that gyroid TPMS outperformed IWP. Furthermore, gyroids exhibit lower or comparable fatigue damage while showing higher stiffness and stress resistance [125]. Dyer et al. investigated the fatigue behavior of Ti-6Al-4V TPMS lattices, including diamond and gyroid structures at 50–70% porosity produced by LPBF. They found that traditional normalization methods could not reliably capture the fatigue response and showed that local stress models and a new instability model offer stronger and more practical predictions [217]. Wang and colleagues examined TPMS lattices fabricated by LPBF using an AlMgScZr alloy with 5 vol% TiCN reinforcement. Results showed that adding TiCN notably improved compressive strength but had minimal impact on energy absorption. Also, among the three TPMS geometries, the diamond structure showed the best load-bearing performance, outperforming gyroid and primitive designs for lightweight applications [218]. Mirzavand et al. investigated modified Primitive TPMS lattices fabricated from PLA reinforced with carbon fiber using FDM. By eliminating curved nodal connections and adjusting strut diameters (1.5–3.5 mm), the yield strength increased from 6.49 to 16.77 MPa without compromising the strength-to-weight ratio. The lattices exhibited up to 90% energy absorption efficiency, with the CFLS-25 design achieving the best balance between weight and performance, underscoring the potential of PLA–CF TPMS structures for lightweight, high-energy-absorbing applications [219]. To investigate the fatigue performance and biocompatibility of TPMS gyroid and stochastic structures, Calvo et al. fabricated the structures via PBF-LB, where the materials were as-built and chemically etched. Their results showed that chemical etching significantly improves the surface quality, along with an increase in mechanical strength and fatigue resistance. They also showed that in terms of mechanical and fatigue performance, gyroid structures outperformed stochastic structures, and etching provided additional benefits [124]. Li and co-workers investigated hydrothermal aging in FDM-printed PLA TPMS lattices with primitive, gyroid, diamond, and IWP geometries. After immersion at 50 °C for up to 30 days, all structures showed reduced compressive strength. Gyroid and diamond retained performance longer, while primitive and IWP degraded faster. The study also showed that perpendicular loading accelerated degradation, emphasizing the combined influence of geometry and build orientation on TPMS durability [220]. Lu and the group investigated the fracture behavior of ceramic TPMS in their study and optimized the mechanical properties through a hybrid design strategy called LCM. Using this hybrid design, they printed gyroid and primitive TPMS structures and proved that hybrid TPMS can enhance mechanical properties [221]. Hu et al. fabricated TiCN-reinforced AlZnMgCu composites via LPBF to improve the strength and cracking resistance of high-strength aluminum alloys. The optimized 5 vol% TiCN composite showed a refined microstructure with UTS of 621 MPa, YS of 538 MPa, and excellent wear resistance.Polymer-infiltrated TPMS lattices made from this material achieved over 186% higher compressive strength and significantly improved energy absorption. These results demonstrate the potential of LPBF Al matrix composites for developing lightweight, high-performance structures [222]. Fan and colleagues investigated the mechanical properties of primitive, IWP, and optimized P-I structures, focusing on their porosity and resistance to the pinch-off phenomenon. According to their experimental findings, yield strength and elastic modulus decreased as porosity increased for all three structures. Also, at the same porosity level, P-I structure has higher compressive strength and elastic modulus than IWP structures [223]. Residual stress significantly impacts the mechanical behavior of the additively manufactured TPMS structures. Huang et al. used a thermal coupling model in their study to analyze the formation of residual stress in TPMS structures (primitive, gyroid, diamond, and IWP). Additionally, they proposed an optimized scheme to minimize these residual stresses. The results showed that due to poor articulation, IWP structures have the most significant residual stress, while D-type structures maintain lower stress levels because of their uniform cross-sections [224]. Hollow glass microballoons filled epoxy composites significantly affect the mechanical properties [225]. Huffman and others developed a photocurable resin reinforced with glass microballoon for the VPP process and characterized the mechanical behavior of Schwarz diamond TPMS structures. Their results showed that the increasing microballoon content improved stiffness, peak stress, and SEA. The DIC and FEA results showed that Schwarz diamond structures exhibited complex strain distributions (shear and axial). They concluded that stiffness and peak stress increased with greater reinforcement, but due to the embrittlement, strain-to-failure decreased [226]. Kim et al. developed a rotated primitive-type auxetic structure (RPAS), where they compared its compressive behavior with the conventional honeycomb spoke. The simulation and experimental results confirmed that RPAS has higher rigidity and stability under compression, particularly on the flat surfaces and obstacles. This variable stiffness property reduces local deformations and enhances shock absorption [227].

Chawla and Kiran studied the effect of a negative Poisson’s ratio on the effective elastic and shear modulus, Poisson’s ratio, and CTE of Schwarz and gyroid TPMS-based composites [228]. Maszybrocka and the group investigated the compression properties of cellular lattice structures with radial gradient TPMS architecture [161]. Shah and others studied the buckling properties of several TPMS structures both experimentally and numerically [30]. Kladovasilakis et al. analyzed the compression properties of gyroid, Schwarz primitive, and diamond structures at 10%, 20%, and 30% relative densities [71]. The fused filament fabrication (FFF) technique was used to print specimens, and the strain rate was set to a compressive strain rate of 5 mm/min. Schwarz’s primitive structure absorbed the highest amount of energy before failure. Gyroid structure exhibited the highest effective elastic modulus among the TPMS structures. Lin, Pan, and Li analyzed the mechanical strength of primitive, S2, IWP, and FRD structures at various relative densities [229]. Results obtained from the three-point bending test were compared with the analytical results. Sixt et al. designed a flexible strain sensor using a Schwarz primitive structure [230]. Additionally, a size optimization was performed using a multi-objective adaptive firefly algorithm (MOAFA). Peng, Marzocca, and Tran investigated TPMS-based honeycomb structures with tunable mechanical responses [231]. Primitive-honeycomb exhibited higher elastic modulus and plateau stress than gyroid-honeycomb at various relative densities. By combining gyroid and primitive structures with a sigmoid function, a gyroid-primitive hybrid structure was created, which displayed distinct properties at different compressive strains. Préve and colleagues used a 3D phase-field model to study fracture behavior in TPMS foams (primitive, gyroid, IWP, diamond, Neovius) under compression. The simulations showed topology-dependent crack initiation and propagation, with Neovius and IWP showing the highest stiffness and strength. Ashby plots confirmed that the TPMS foams outperform conventional aluminum foams, demonstrating their superior mechanical efficiency and suitability for lightweight structural applications [232]. Gawronska and Dyja numerically showed the impact of geometry on the thermal and mechanical properties of TPMS structures [233]. They observed reduced maximum displacement for structures with increased relative thickness. Stress analysis showed that all parts of the primitive surface participate in stress distribution. In contrast, diamond and gyroid surfaces contain some volumes with very low effective stress values, suggesting that they do not contribute to a structure’s overall strength. Wu and the team investigated the flexural behavior of Cu-Cr-Zr lattice beams with gyroid, primitive, and IWP TPMS architectures fabricated by SLM. Three-point bending tests and simulation results showed that graded designs outperformed uniform ones, increasing bending strength by up to 38.5% and energy absorption by 66.6%. Among the geometries, the gyroid structure exhibited the best performance, confirming gradient design as an effective approach to enhance strength and crashworthiness in TPMS beams [234].

Dalaq et al. investigated the mechanical behavior of novel types of three-dimensional (3D) architectured two-phase interpenetrating phase composites (IPCs) both experimentally and computationally [235]. They also explored the anisotropy induced by 3D printing. The Schwarz primitive reinforcement was found to be the least susceptible to debonding and provided a significant improvement in all mechanical properties, including toughness, ductility, and strength. The anisotropic behavior of primitive, gyroid, diamond, and IWP surfaces was analyzed by rotating around the [96], [106], and [105] axes at multiple angles [236]. Based on the FEA results, a gradient rotation structure was designed to improve the energy absorption characteristics of TPMS. Khalegi and the team generated hybrid structures by combining primitive and Neovius structures with other TPMS, obtaining an optimal combination ratio of the parent structures that resulted in the least universal anisotropy [237]. Quasi-static loading and dynamic loading were applied to several TPMS structures [96]. The IWP structure offered the highest plateau stress under both loading conditions, while the primitive structure exhibited the lowest plateau stress levels. The diamond lattice structure demonstrated the highest SEA capacity, followed by the gyroid, IWP, FKS, and, lastly, the primitive structure. Park et al. studied the tensile properties and deformation behavior of Neovius and IWP structures for different unit cell sizes [156]. Relative densities and yield strengths of both structures were decreased, and the elongations increased with the increase in unit cell size.

**Figure 9:**
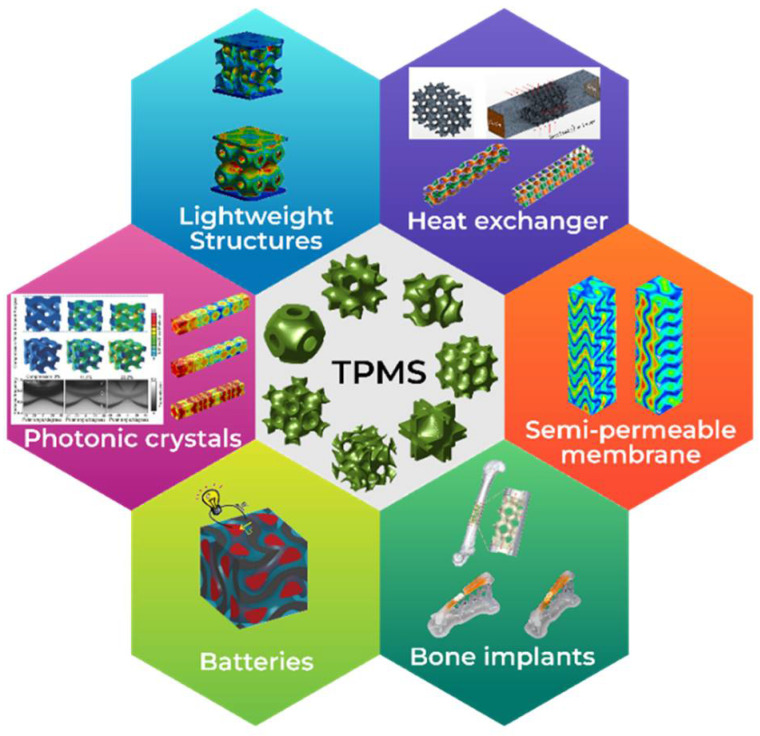
Different applications of TPMS units.

Superelasticity and the shape memory effect are two unique material properties of shape memory alloys (SMAs) that are not found in conventional materials. They are utilized in a variety of cutting-edge fields, particularly biomedicine, corrosion resistance, fatigue, and aerospace. By using SMAs as the base material, superior materials can be produced that inherit the distinctive properties of both TPMS and SMAs. The superelastic deformation of SMA primitive-foam cellular structures is enhanced by lower density and reduced temperature [238]. Viet and Zaki proposed a novel artificial neural network-based framework to investigate the mechanical behavior of the SMA Schwarz primitive TPMS structure [239]. Components made with NiTi alloy exhibit shape recovery under stress-free conditions. Sun and colleagues studied the mechanical and shape memory properties of three NiTi TPMS structures (primitive, diamond, and gyroid) [240]. Figure 9 illustrates an overview of the applications of TPMS structures across various domains.

### 6.2. Heat Transfer Applications

TPMS are an excellent choice for developing low-temperature waste heat recovery systems due to their outstanding thermophysical properties. To enhance efficacy, compactness, and lightweight designs, TPMS-based structures have been employed in heat exchangers (HEXs) across several studies [241,242]. Liu et al. investigated the characteristics of the TPMS, which was fabricated via the LPBF process. The results showed that among the homogeneous TPMS structures, the primitive TPMS performs better under forced convection, while the gyroid TPMS excels at natural convection. They also found that the average convection heat transfer coefficient increases in the P-Quadratic II (Quadratic primitive) structure, demonstrating the best overall heat transfer performance under both forced and natural convection [243]. Guillermo and the team conducted a numerical study on fluid flow and heat transfer in gyroid TPMS under incompressible, steady conditions, varying the inlet velocity, porosity, and working fluid. They validated their numerical model against experimental data, providing an effective tool for predicting pressure drop and heat transfer performance [244]. Wang and colleagues designed and evaluated a crossflow heat exchanger utilizing gyroid TPMS. They used hot air and cold aviation kerosene as the working fluids. The numerical findings demonstrated that the gyroid TPMS structure significantly enhances fluid mixing and heat transfer by inducing secondary flows (split-merge, parallel, and circulation flows). Their study emphasized the importance of using AM to fabricate a thin-walled TPMS structure for efficient heat transfer [245]. Kruzel, Dutkowski, and Bohdal designed a gyroid TPMS heat exchanger via 3D printing. They tested the flow to analyze pressure drops by varying temperatures and mass flow rate. The results showed that the conventional flow resistance model does not apply to gyroid heat exchangers. Based on their experiments, they calculated the friction coefficient (f) to develop a new equation that predicts the flow resistance in TMPS structures [246]. For aerospace applications, Lai et al. designed and analyzed the performance of a gyroid-based TPMS exchanger, varying the flow rates and temperatures. Their experimental and computational results showed that the gyroid HEX outperforms the conventional plate HEX, exhibiting higher stiffness. Additionally, they found that an aluminum gyroid HEX has a higher heat transfer rate than a stainless steel HEX [247]. Reynolds, Lecarpentier, and Holland evaluated gyroid TPMS heat exchangers fabricated from polymers, metals, and ceramics, showing up to 10% deviation in porosity and hydraulic diameter between design and printed structures. Using corrected μCT parameters, consistent Nusselt–Reynolds correlations were obtained across all materials, highlighting the importance of geometric fidelity in TPMS-based heat exchanger performance [248]. Men and others proposed a topology optimization method for IWP TPMS to enhance heat exchanger performance, using an effective porous media model. By establishing connections between the porosity and effective factors, the model simplifies flow and heat transfer calculations. The results showed that the optimized IWP TPMS significantly reduces peak temperature and pressure drop [249]. While traditional heat dissipation method struggles under extreme conditions and compact designs, gyroid TPMS enhances heat transfer due to its compact design and thermophysical properties. Qin and the group introduced a control factor (β) to modify the gyroid surface, inducing “wrinkles”. Their numerical and experimental results showed that as the value of β increases, peak temperature decreases along with an increase in the convective heat transfer coefficient and Nusselt number. This approach offers an avenue for improving TPMS heat transfer efficiency [250]. Renon and Jeanningros numerically studied the thermohydraulic performance of the Schwarz-diamond and Schoen-gyroid TPMS-based heat exchange channels. They found that the Schwarz-diamond and Schoen-gyroid exhibit similar thermal performance, although the Schoen-gyroid has higher pressure drops [251]. Yan and the team designed TPMS-based HEX by introducing two hybridization methods, Proportional Hybridization (PHM) and Sigmoid Function (SF). Using the AM technique, they fabricated gyroid and Schwartz diamond TPMS-type heat exchangers. The result indicated that the gyroid has the lowest pressure drop and the highest Nusselt (Nu) number. Both techniques successfully improved the efficiency of TPMS heat exchangers, presenting innovative approaches for high-performance, lightweight heat exchangers [252]. Yan et al. conducted an experimental and computational evaluation of a 316L stainless steel gyroid heat exchanger manufactured using SLM. The calculated correlation, Nu = 0.471Re^0.627^ Pr^1/3^ (150 < Re < 3000), aligned well (±7.5%) with the experimental data. The gyroid structure proved suitable for high-performance, compact applications by outperforming conventional designs in heat transfer efficiency despite a larger pressure drop [253]. Li, Li, and Yu numerically investigated the thermal and hydraulic performances of the TPMS Schwarz-diamond and TPMS Schoen-gyroid heat exchangers [254]. A printed circuit heat exchanger (PCHE) was a reference heat exchanger. Attarzadeh, Rovira, and Duwig also analyzed the performance of Schwarz-diamond heat exchangers for recycling waste heat in low-pitch thermal systems [178]. Wadso and Holmqvist investigated the performance of both gyroid and diamond, utilizing experimental and numerical methods [255]. Aluminum alloy AlSi10Mg was used for manufacturing these structures. Gao et al. conducted the first experimental study examining the impact of TPMS cell size and its gradient on additively manufactured heat exchangers [256]. They focused on the diamond surface and manufactured heat exchangers with pure copper using SLM technology. A smaller cell size resulted in a more uniform temperature distribution and a higher pressure drop. The cell size of 4 mm yielded the highest effectiveness and a greater volume-based power density. Yan and colleagues numerically studied four TPMS-structured heat exchangers [257]. Under varying mass flow rate conditions, the mixing performance of the diamond structure outperformed gyroid and IWP structures. Oh et al. proposed a multifunctional gradation strategy for TPMS-based heat exchangers, integrating filtering, cell-size, and level-set gradations to balance efficiency and strength. The graded gyroid design enhanced heat exchange capacity by 30% while maintaining a minimal pressure drop. This resulted in a 28% overall performance improvement, demonstrating the effectiveness of gradation in optimizing TPMS heat exchanger performance [258]. Gado, Ookawara, and Hassan implemented TPMS structures in adsorbers to enhance the performance of adsorption cooling systems (ACSs) [259]. TPMS-based structures surpassed fin structures in specific cooling power (SCP). Compared to the Lidinoid and Fins structures, gyroid, diamond, and primitive structures showed faster kinetics. Fan and others proposed a P-IWP type TPMS to enhance the local heat transfer of PCM [260]. The TPMS-based Battery Thermal Energy Storage (BTES) system reduced the temperature rise of the battery and extended the duration of the unit temperature drop.

TPMS nowadays are widely used in various applications, including microprocessor cooling. Ansari et al. used a gyroid-based TPMS structure as an advanced thermal management solution for this purpose. Their numerical investigation compared the cooling effectiveness of the gyroid TPMS with the conventional pinfin heat sinks (PHS) for electronic devices. The results showed that the gyroid heat sink consistently exhibits superior thermal efficiency compared to the pinfin heat sink. However, it requires higher pumping power due to an increased pressure drop [261]. Also, for electronic cooling, Chen and colleagues compared TPMS-based cold plates with diamond, gyroid, and IWP geometries against a conventional serpentine design. Results showed that the diamond structure exhibited superior heat transfer and temperature uniformity compared to other designs. The graded diamond variant further achieved a heat flux of 256.9 W/cm^2^ at low pumping power, underscoring the potential of TPMS architectures for advanced liquid cooling systems [262]. Barakat and Sun explored the heat transfer performance of new TPMS structures (TPMS4 and TPMS5) compared to diamond, gyroid, SplitP, and Lidinoid models through airflow experiments and numerical simulations. The results showed that the new TPMS structure exhibits superior heat transfer performance, with numerical improvements in the NU and convective heat transfer coefficient, outperforming the other TPMS structures [263]. Brambati, Guilizzoni, and Foletti investigated the use of TPMS HEXs and highlighted their advantages in enhancing heat transfer due to increased surface area and improved convection. The convective heat transfer coefficient is predicted to use a single heat transfer correlation for various fluids, unit cell sizes, porosities, turbulent flow regimes, and TPMS topologies (gyroid, Schwarz-primitive, and Schwarz-diamond). The accuracy of the proposed correlation surpasses that of conventional and existing TPMS correlations, particularly when adjusted to incorporate total temperature in high-speed flows [264]. Wang and the group, in their study, investigated the use of TPMS to improve the heat exchanger efficiency and reduce flow resistance. The surface area and porosity were accurately controlled using the FKS TPMS structure. Numerical simulations and tests were conducted to evaluate complex geometries fabricated using SLM. Results showed a strong correlation between Nu and Reynolds (Re) numbers for the FKS structure [265]. Surface roughness significantly impacts the mechanical and thermal properties of the additively manufactured materials. Yan and others used abrasive jet polishing to address the surface roughness problem in additively manufactured gyroid-structured heat exchangers. The results revealed that abrasive jet polishing greatly minimizes interior surface protrusion microstructures. Additionally, with reduced surface roughness and wall thickness, the polished prototype decreases differential pressure by 40% while maintaining nearly constant heat transfer capacity. Moreover, the polished gyroid structure’s Nu~Re and f~Re correlations align well with numerical and experimental data. The heat transfer performance is considerably better, even though the friction factor exceeds that of offset strip fin heat exchangers and traditional PCHEs [266]. Zhang et al. quantitatively examined the impact of model height on the thermal performance of latent heat thermal energy storage (LHTES) systems with TPMS structures (IWP and primitive) compared to conventional BCC foams. Their pore-scale simulations showed that thermal conduction was significantly decreased with increasing model height (15–45 mm), with IWP exhibiting the most significant drop (49.4%). In IWP, convective heat transmission remained relatively constant, while in primitive and BCC structures, it initially increased and then declined with height. Notably, the primitive structure performed better at greater heights. In contrast, IWP performed better at lower heights, underscoring the significance of geometry-dependent structural choices for maximizing the solar thermal storage efficiency [267]. TPMS structures are nowadays used in the aerospace industry. Huang and colleagues used CFD to assess the diamond and FKS TPMS structures for aero-engine regenerators. The results showed that in contrast to PCHEs, TPMS structures improved their performance by 10.9–32.5%. They also increased the power-to-weight ratio by up to 4.8 times and enhanced heat transport by up to 86% [268]. Pelanconi et al. fabricated SiC ceramics with gyroid TPMS architectures using a hybrid process combining PBF, BJ, and chemical vapor infiltration (CVI). After two CVI cycles, BJ specimens showed clear densification and reached a compressive strength of approximately 13.3 MPa. Oxidation tests at 1500 °C revealed the formation of protective SiO_2_ layers, confirming the potential of AM–CVI hybrid processing for producing oxidation-resistant SiC TPMS structures for high-temperature and energy applications [269].

Qureshi et al. compared the heat transfer performances of TPMS-based foams with those of conventional metal foams. All TPMS foams outperformed conventional metal foams in pure conduction and natural convection by reducing the melting time of the PCM and increasing the average heat transfer coefficient. In pure conduction, the IWP foam outperformed both gyroid and primitive cells [270]. Femmer, Kuehne, and Wessling prototyped micro heat exchangers using the DLP method and implemented primitive, diamond, gyroid, and IWP structures. Experiments indicated that Schwarz-diamond had the lowest friction-to-heat transfer ratio among the other TPMS structures [271]. Dharmalingam and others computationally analyzed the thermal-hydraulic performance of three FK TPMS structures. The results showed that FK HEXs outperform the Schwarz-diamond heat exchangers. Compared to the other TPMS geometries considered in the study, the FKS TPMS HX demonstrated a significantly better thermal-hydraulic performance [272]. Zhang et al. numerically investigated anisotropies in the thermo-fluid behavior of gyroid and diamond-based sandwich panels. Across all rotating angles, diamond outperformed gyroid in terms of heat transfer and pressure drop [273]. Fan, Gao, and Liu developed a TPMS-based phase change material system to enhance heat transfer and avoid thermal saturation in the battery thermal management system (BTMS). Results showed that the hybrid PCM/liquid/TPMS design reduced battery temperature by 40% at a 2C discharge rate. A lower PCM phase-transition temperature of 35 °C further improved cooling by 8.5%, demonstrating a compact and efficient approach for advanced battery thermal management [274]. Penubarthi and colleagues investigated the fluid transport behavior of SLA-printed porous structures, comparing TPMS geometries (gyroid, diamond) with lattice-based Octet and Isotruss designs. Using high-speed imaging and numerical simulations, they examined droplet dynamics, contact angles, and permeability. The results showed that unit cell geometry strongly governed capillary action and wettability, with the gyroid structure exhibiting superior capillary rise and the Octet achieving the highest permeability. The study highlighted the geometry-dependent nature of fluid transport in porous architectures and provided valuable insights for designing structures for efficient cooling and filtration applications [275].

### 6.3. Biomedical Applications

TPMS structures find extensive application within the realm of tissue engineering, primarily due to the resemblance of TPMS to trabecular bone, which possesses nearly zero mean curvature. Rajagopalan and Robb presented the first examples of tissue engineering scaffolds generated from TPMS [276]. Kapfer et al. explored a large number of bicontinuous TPMS and distinguished between sheet solids and network solids [277]. To maximize stress distribution and longevity, Alemayehu and colleagues investigated the biomechanical performance of completely and hybrid gyroid TPMS latticed dental implants. Six implant designs were assessed, including hybrid implants with solid necks (HI_111, HI_222, and HI_333) and fully latticed implants (FLI_111, FLI_222, and FLI_333). Stiffness and flexibility were balanced using FEA in nTopology, and dynamic mastication loading was simulated. The findings demonstrated that hybrid implants, particularly HI_222, had better stress distribution, enhanced osseointegration, and reduced micromotions [278]. Baumer and others fabricated FKS ceramic scaffolds for bone tissue engineering (BTE) for the first time, and they compared them to the commonly utilized gyroid topology. Both scaffold types demonstrated reliable quasi-brittle failure behavior and mechanical characteristics throughout the lower range of trabecular bone. Their results proved that FKS ceramic scaffolds outperform gyroid in strength, energy absorption, and reliability, positioning them as more suitable for bone regeneration applications [279]. Cheloni et al. studied four lattice structures: BCC, gyroid, diamond, and Voronoi (graded and regular), fabricated via Ti-6Al-4V using PBF-LB. The results showed that diamond structures have the highest strength and energy absorption. The simulation results also showed that in TPMS, stress distributions are more favorable, while BCC/Voronoi structures have stress concentrators, leading to lower collapse loads [280]. D’Andrea et al. explored the 3D-printed hydroxyapatite TPMS scaffolds using VPP for BTE in their study. They worked with diamond, I-graph, IWP TPMS scaffolds. The experimental data closely resembled the numerical data. The findings indicated that VPP can effectively print intricate TPMS structures with thin (hundreds of micrometers) walls. Furthermore, they demonstrated that diamond structures had the lowest specific strength among the TPMS geometries, whereas I-graph and IWP scaffolds exhibited the highest strength [281]. Firoz and the group investigated 3D printed biodegradable PLA/Fe_3_O_4_ gyroid scaffolds, varying the infill densities for bone repair. It showed that at 50% porosity, PLA/Fe_3_O_4_ composites exhibited ferromagnetic behavior and mechanical characteristics suitable for cancellous bone healing. Notably, up to 85% of the shape memory effect was observed in porous scaffolds, while 100% was seen in nonporous PLA ribbons. Emphasizing architectural improvements and in vivo testing under magnetic fields, their results suggest that PLA-based scaffolds may be used in soft robotics, artificial muscles, and bone regeneration [282]. Guneshekar and colleagues investigated the TPMS lattices for bone implants, and they analyzed their mechanical properties and anisotropic behavior. The compression test and numerical results showed that the lattices exhibited stretch-dominated behavior, while minor deviations were due to the manufacturing inconsistencies. Their results provided important information for creating next-generation TPMS lattices for biological and load-bearing applications [283]. Wang et al. fabricated four TPMS gyroid scaffolds (TG15, TG20, TG25, TG30) via the SLM method. They examined the impact of the unit cells in TG titanium scaffolds on osseointegration. The results showed that TG15 and TG20 exhibited maximum compressive strengths, while TG20 demonstrated superior biological performance, enhancing osteogenic differentiation, cell adhesion, and proliferation. The effectiveness of TG20 has also been confirmed in vivo, indicating its potential as a scaffold for BTE applications [284]. Due to the widespread use of TPMS structures for making the scaffolds, Koushik, Miller, and Antunes investigated the effects of scaffold architecture on mechanical strength, biofunctionality, and osteointegration in BTE. The results showed that the split-primitive structure had the lowest surface area for bone apatite precipitation, while it had the maximum compressive strength. On the contrary, in simulated bodily fluid, gyroid and Lidinoid structures exhibited greater bone apatite production [285]. Araya et al. investigated the mechanical characteristics of Ti-6Al-4V lattice structures fabricated by LPBF, focusing on TPMS gyroid and Stochastic Voronoi structures. They found that in all densities, gyroid structures exhibited more stiffness due to their stretch-dominated behavior, while stochastic structures displayed bend-dominated responses. The results provide valuable information for enhancing lattice manufacture and design [93]. Pore architecture plays a vital role in the mechanical performance of bone scaffolds. Bakhtiari, Nouri, and Tolouei-Rad showed in their study that the pore architecture impacts the mechanical performance of PLA bone scaffolds under quasi-static and cyclic compression. They used an FDM 3D printer to print the scaffolds at 60% porosity with gyroid, Lidinoid, FK, IWP, and Voronoi structures. It proved that gyroid scaffolds have the highest compressive strength, and the strength increased with the strut thickness. However, in the case of fatigue, Voronoi shows the best performance. Overall, gyroid topology was shown to be the best design for bone scaffolds when considering both static and fatigue strength [181]. Chen studied the manufacturing of NiTi shape memory alloys for biomedical applications using LPBF, with an emphasis on dense and TPMS gyroid structures. According to their study, graded gyroid cellular structures (GCSs) developed by LPBF exhibited elastic moduli (5–7 GPa) that mimicked the human bone and displayed tunable mechanical and fatigue behavior. They also showed that among the designs, Z-GCSs (graded parallel) displayed distinct layer-by-layer failure modes, while Y-GCSs (graded perpendicular to load) had better fatigue resistance. Their results demonstrate the potential of gyroid TPMS architectures for improving load-bearing implant performance by controlling deformation response and porosity [286]. Melchels compared gyroid architecture with random pore architecture [287]. Their study showed that gyroid architecture exhibits over a tenfold increase in permeability compared to structures with size-limiting pore interconnections. This increased permeability noticeably enhanced the wetting characteristics of hydrophobic scaffolds and accelerated the cell settling rate during the static seeding of immortalized mesenchymal stem cells. Yoo discussed the porous scaffold modeling method using different TPMS structures. Hexahedral brick elements were implemented to generate scaffold architecture [288]. Tikhonov et al. studied the SLA fabrication of three-dimensional permeable scaffolds from calcium phosphates CaP/PEGDA hydrogel biocomposites for use as bone grafts [289]. Tsai and Cheng conducted a study on the behavior of surgical screw insertion and extraction in bone implants with TPMS structures [290]. On both the TPMS and solid bone implants, the pullout strength is found to be closely associated with the maximum insertion force. Furthermore, the TPMS bone implants have significantly larger pullout strengths. TPMS implants effectively constrained the damaged area during screw insertion, resulting in superior fixation stability compared to the solid bone. For additive and lithographic fabrication processes such as DLP, inkjet printing, and nanoimprint lithography, Kainz et al. presented a thiol-ene photocurable resin. This resin enabled the creation of biomimetic scaffold architectures spanning six orders of magnitude, from millimeter-scale TPMS lattices to nanoscale surface features. Their results showed that among the successful prints, TPMS-based gyroid structures revealed outstanding fidelity and structural plasticity. The low cytotoxicity, hydrolytic degradability, and good mechanical properties make this resin suitable for the scaffold design in biomedical applications [291]. Shen and colleagues studied bioceramic scaffolds with TPMS geometries, which were fabricated using the DLP technique [292]. The study revealed that gyroid and diamond pore scaffolds have a notable capacity to promote osteogenic differentiation in bone marrow mesenchymal stem cells (BMSCs). Li and others introduced a gradient porous scaffold inspired by the Haversian system, utilizing TPMS architectures with pore size varying from the edge to the center [293]. The gradient TPMS scaffold demonstrated appropriate shear stress and larger permeability, facilitating better bone ingrowth. Liu et al. proposed a 3D deformation equation of TPMS. They studied the effects of the c value of TPMS, periodic function η, and deformation index γ on the mechanical properties of the auxetic structure [123]. They also analyzed a heterostructure based on primitive and gyroid surfaces, applicable to hip joint design. Belda and the group conducted a numerical investigation to analyze the correlations between morphometry and mechanical properties for TPMS configurations in designing bone implants [294]. Gyroid, diamond, FK, and IWP architectures were analyzed. Their work provided a framework for selecting the appropriate TPMS configuration for patient-specific applications. Maevskaia et al. investigated diamond, gyroid, and primitive microarchitectures for BTE [183].

Blanquer and colleagues designed eight TPMS-based scaffolds and fabricated them by SLA using a photocurable resin called polytrimethylene carbonate (PTMC) [295]. Micro-computed tomography (μCT) analysis was employed to examine the internal 3D structures, revealing a fabrication accuracy of approximately 94% to 98% for SLA. The highest intrinsic water permeabilities were found in the scaffolds with pore channel architectures (primitive, diamond, and gyroid). Zou and others proposed an enhanced porous scaffold based on TPMS and investigated their manufacturability, biocompatibility, and mechanical properties [154]. The permeability coefficient of the porous scaffolds was experimentally measured using the constant head method and was found to be consistent with the range of human bones. Zhu et al. studied the Schwarz primitive structure to design porous scaffolds with optimized thickness [164]. The numerical investigation revealed that the scaffolds with optimized thickness had lower permeability due to the rough inner surface. Hayashi and the group experimentally validated that gyroid scaffolds have better cell migration and tissue ingrowth compared to grid scaffolds [117]. Furthermore, the gyroid structure showed higher mechanical strength and porosity. Kumar, Birru, and Muthu optimized gyroid TPMS scaffolds made from PLA/MgTiO_3_ composites using FDM for bone tissue engineering. A multi-response framework combining Taguchi, VIKOR, and TOPSIS methods identified optimal parameters, achieving a compressive strength of 45.7 MPa and Young’s modulus of 0.47 GPa. The addition of MgTiO_3_ enhanced mechanical strength, energy absorption, and bioactivity, making the scaffolds suitable for load-bearing biomedical applications [296]. In another study, Kumar and colleagues designed and fabricated gyroid TPMS hybrid scaffolds using the FFF process with PLA/MgTiO_3_ composites. The incorporation of MgTiO_3_ enhanced thermal stability, compressive strength, and surface wettability. It also promoted apatite formation, mesenchymal stem cell differentiation, and antibacterial activity against *E. coli*. These results demonstrate the strong potential of PLA/MgTiO_3_ TPMS scaffolds for bone tissue engineering applications [297]. To develop optimized architectures for intervertebral lumbar cages, Reshadinezhad et al. designed Ti-6Al-4V TPMS lattices via SLM and assessed their mechanical response. Gyroid and diamond structures with 70–75% porosity exhibited elastic moduli (9–16 GPa) comparable to human bone, indicating their suitability for cage fabrication. In contrast, Schwarz lattices were limited by manufacturability and lower surface-to-volume ratios. The finite element simulations confirmed that these TPMS cages effectively minimized stress shielding and maintained safe stress levels under compression, shear, and torsion [298].

Karuna and colleagues studied the mechanical and fluid characteristics of graded IWP structures [299]. Samples were manufactured using Ti-6Al-4V by the LPBF technique. Following the baseline presented by Li et al., samples were constructed to represent trabecular and cortical bones [300]. Grading features such as local relative density, unit cell size, pore size, and wall thickness were found to have a significant impact on the effective modulus and fluid permeability. Conversely, Gaussian curvatures and the wall shear stress induced by fluid flow experienced relatively minor impacts. Machine learning (ML) is nowadays widely used in TPMS structure optimization and other applications. Wu and others used a machine learning (ML)-based method for optimizing ceramic scaffold design for bone regeneration. To satisfy biomechanical requirements, the functionally graded TPMS tissue scaffolds were created by ceramic 3D printing and integrated with Bayesian optimization (BO). The proposed approach allows for minimal computing cost and time-dependent mechano-biological optimization. A simulation was conducted on a sheep tibia segmental defect, which demonstrates that optimal scaffolds significantly enhance bone ingrowth [301]. FEM analyses are also used for the mechanical behavior of biomedical implants. Ziaie and the group modeled solid and sheet network TPMS structures (gyroid, diamond, and primitive) where the unit cell sizes ranged from 1 to 2.5 mm and varied the porosity. They ensured compatibility with biomedical applications such as cell seeding, vascularization, and osseointegration. Their results showed that an optimal pore size is between 500 and 1000 μm, while the unit cell size is 2.5 mm [302].

### 6.4. Chemical Applications

Werner et al. created a 3D gyroidal nanohybrid for solid-state lithium-sulfur batteries using block copolymers. This innovation integrates the anode, electrolyte, and cathode into a co-continuous architecture with feature sizes of less than 20 nm. Compared to earlier nanoscale designs, the self-assembled structure achieved an open-circuit voltage of 2.8 V and an aerial capacity that is 45 times larger, facilitating efficient ion transport and ensuring mechanical stability. This bottom-up strategy provides a scalable framework for future 3D solid-state energy [303]. Yang and colleagues proposed a hybrid BTMS that combines a temperature-triggered delayed cooling system with TPMS-structured PCM. Their findings indicated that the system maintained safe battery temperatures across various operating conditions, while improving thermal conductivity (21.3 W/m·K), PCM utilization to 97%, and pump energy use to 73%. Furthermore, the system demonstrated its resilience for electric car applications by performing well in a variety of environmental conditions [304]. Zhang and others demonstrated that heat conductivity and melting capability were significantly improved by incorporating 3D-printed TPMS aluminum lattices into paraffin-based PCMs. They found that the melting time decreased by approximately 20 min with a 10% TPMS structure, and the GS-65-20 design exhibited the highest heat storage rate (1.85 J/s), which is 1.5 times that of pure PCM [305]. Qureshi and the group conducted a numerical study comparing sheet and solid configurations of TPMS (gyroid and IWP) embedded in low-conductivity organic PCM (docosane) for latent heat thermal energy storage. Their results showed that IWP sheet structures exhibited the highest thermal conductivity and fastest melting time (118 s) under pure conduction, while IWP solid performed best with natural convection (108 s). Additionally, IWP sheets demonstrated greater temperature uniformity under isoflux conditions, underscoring the significance of boundary conditions and architecture in PCM-TPMS composite design [306].

Lei et al. used the SLM technique to create a porous stainless steel catalyst support with a D-type TPMS architecture. In comparison to commercial felts, the structure demonstrated superior hydrogen production in methanol steam reforming due to its high porosity (82.1%) and improved catalyst adherence [307]. TPMS-based spacers increase turbulence and reduce the boundary layer at the membrane surface, thereby improving the heat transfer coefficient (63%) in membrane distillation. Thomas and colleagues assessed the impact of several TPMS spacers, finding that the transverse CLP (tCLP) spacer had the best water flux performance (60%) [137]. Sreedhar and the group designed and fabricated several TPMS-based spacers and investigated their impact on mass transfer, pressure drop, and critical flux in a flat-sheet ultrafiltration (UF) setup for protein separation in an aqueous environment. All the TPMS spacers exhibited better mass transfer performance than the commercial spacers. Gyroid and CLP spacers showed the highest Sherwood number values at any given Re [308]. Hawken et al. studied Schwarz-diamond TPMS structures and proposed two new correlations for predicting pressure drop across various porosities and hydraulic diameters. To produce high-quality printed components without notable channel blockage, the hydraulic diameter should be greater than 2 mm, and the porosity should be greater than 40% [309]. Terret and Frankcombe simulated Ni/Al bimetallic nanostructures based on Triply Periodic Minimal Surfaces (Gyroid, Schwarz P, Schwarz D, and Neovius) and compared them with conventional nanolaminates. Results showed that TPMS architectures exhibited lower ignition energy, faster combustion, and greater heat release. Gyroid and Schwarz D structures achieved rapid self-sustained reactions, highlighting their potential for advanced energetic and reactive sintering applications [310]. Bertero et al. fabricated TPMS-based mullite monoliths (Schwartz primitive and gyroid) via DLP and functionalized them with HKUST-1 for CO_2_ capture. The gyroid design provided higher surface-to-volume ratios, lower pressure drops, and longer retention times compared to conventional powder systems. These findings highlight the strong potential of TPMS–MOF hybrids for efficient carbon capture applications [311]. Yu and the team demonstrated the integration of TPMS architectures into 3D-printed concrete systems to advance carbon capture and utilization. The tailored TPMS geometries enhanced CO_2_ absorption efficiency by maximizing surface area, facilitating mass transport through interconnected porosity. Their work highlighted the potential of functionally architected materials in addressing scalable, sustainable carbon mitigation strategies [312].

Xu and the group have developed a machine learning model to optimize the framework for TPMS-based porous structures, enhancing solar thermochemical fuel production. To predict reaction efficiency, fuel yield, and thermal gradients, neural network models were trained using high-fidelity 3D multiphysics simulations. In contrast to the reference scenario, their findings demonstrated that gradient structures with c_1_ = c_2_ = 0.5 and ω_1_ = 0.2, ω_2_ = 0.8 significantly reduced thermal gradients while achieving a 58% improvement in efficiency and an approximately eightfold increase in fuel production [313].

### 6.5. Other Applications

Desiccant air conditioning (DAC) systems have gained attention due to their significant benefits in integrating with renewable energy sources, providing effective moisture management, and employing greener refrigerants compared to traditional vapor-compression air conditioners [314]. Kurup et al. studied the aeroelastic flutter of uniform and functionally graded TPMS beams [315]. Increasing relative cell density led to higher critical aerodynamic pressure and fluttering frequency. Abueidda and colleagues conducted a comprehensive computational investigation on PMMA-based cellular solids derived from TPMS. The study focused on three TPMS geometries: primitive, IWP, and Neovius, and examined the acoustic band gap characteristics and uniaxial elastic properties of these architectured materials. The study demonstrated that Neovius structures exhibited the widest and lowest-frequency complete band gaps, while all TPMS architectures displayed tunable acoustic and mechanical performance as a function of porosity. These results underscore the potential of TPMS-based materials for lightweight and multifunctional acoustic metamaterial applications [316]. Hur, Hennig, and Wiesner conducted a systematic computational investigation of 16 bicontinuous cubic network structures and identified six that exhibit complete phononic bandgaps. Among these, the IWP structure demonstrated the largest bandgap width (0.41). The study elucidated the influence of network topology, strut thickness, and material density on bandgap formation, providing valuable design guidelines for developing phononic materials for acoustic and thermal applications [53]. Dolan and others investigated the origin of linear dichroism in gyroid optical metamaterials and attributed it to anisotropic surface terminations that disrupt the inherent bulk cubic symmetry. The study highlights the critical role of nanoscale surface morphology in influencing optical responses, emphasizing that surface effects can dominate over bulk structural properties in metamaterial design [317]. Tran and Peng utilized machine learning models to predict the photonic bandgap characteristics of complex self-assembled gyroid structures. Their approach enabled efficient exploration of design parameters, thereby accelerating the discovery and optimization of gyroid-based photonic crystals for advanced optical applications [318]. Zhang et al. fabricated functionally graded TPMS structures via DLP and demonstrated their superior mechanical strength and energy absorption capabilities compared to uniform TPMS counterparts. The study underscores the potential of graded TPMS architectures for lightweight structural and impact-resistant applications [31]. Feng and the group found that the titanium alloy-made TPMS scaffolds concentrated deformation during the damping phase, significantly increasing the energy dissipation. The resultant composite outperformed traditional and commercial damping materials with a loss modulus of up to 1.54 GPa. Their research demonstrates how well TPMS-based lattice structures allow high-specific-damping materials for controlling structural vibration, particularly in aerospace applications [319]. Foamed TPMS structures are well-suited for automotive and aerospace applications where lightweight structures are needed to absorb energy. Weber and others applied a gas foaming process to gyroid, FKS, and PMY structures to investigate the benefits of higher porosity on energy absorption [320]. In both unfoamed and foamed conditions, the PMY structure exhibited the highest energy absorption. Ellebracht and colleagues assessed gyroid, diamond, and primitive as advanced packing geometries for CO_2_ capture and evaluated their effectiveness compared to a conventional structured packing, Mellapak 250Y [321].

Abueidda et al. investigated the effective electrical and thermal conductivities, as well as the elastic moduli of TPMS foams [322]. TPMS foams exhibiting cubic symmetry displayed similar effective conductivities, with the conductivity varying linearly. Schwarz CLP foam was found to have the highest anisotropy among the other TPMS foams. Kolibaba and the group applied a nanobrick wall flame-retardant coating layer-by-layer to vat photopolymerized gyroid TPMS lattices to increase their fire resistance. The coated gyroids demonstrated a significant improvement in the time to failure (up to 340%) and a better compressive modulus in large-cell designs. In contrast, uncoated lattices showed decreased fire resistance due to their high surface area. Their research demonstrates how surface coatings and TPMS geometry work together to create lightweight, fire-safe lattice structures for extreme applications [323]. Li and others developed self-healing, electrically conductive poly(dimethylsiloxane) PDMS composites by wrapping poly(dimethylsiloxane) covalent adaptable networks (PDMS-CANs) with single-walled carbon nanotubes (SWCNTs) and fabricating them using SLS. To achieve an ultralow percolation threshold (0.007 wt.%) and excellent multifunctionality, such as strain sensing and self-healing fueled by heat, electricity, or NIR light, they constructed various kinds of self-healing lattice structures, including TPMS Schwarz surfaces. According to the finite element modeling, TPMS Schwarz structures possessed the highest strain sensitivity among the tested designs due to the localized strain concentration [324]. TPMS structures exhibit significant potential for use as HEXs due to their enhanced surface-to-volume properties and robust designs that are capable of withstanding mechanical loads. Lesmana and Aziz developed metal-hybrid-based hydrogen storage using the gyroid structure [325]. They numerically evaluated the capability of this structure to withstand the working pressure and load. Forced convection was found to be a preferable cooling solution that requires low energy consumption while providing adequate cooling. The gyroid reactor was manufactured using AlSi10Mg powder via SLM. The experiment showed that the increased surface area inside the TPMS reactor significantly improved the internal heating [326]. Using equation-based modeling, Ribeiro and the team presented a unique TPMS-based design for gas–liquid contactor packing that allows for fine control of strut geometry and cavity formation. The improved TPMS structure improved the gas–liquid interaction and liquid film stability. Experimental and CFD results demonstrated potential for advanced separation processes by confirming lower pressure drop and better performance compared to traditional packings [327]. Liu et al. used the FDM technique to create TPMS IWP lattices using hybrid configurations and materials (PLA, PLA-CF, and PDMS). They achieved multi-stage energy absorption, programmable shape recovery, and temperature-dependent mechanical behavior. Their research showed that hybrid densities and material designs greatly enhance vibration isolation and energy absorption, enabling a promising prospect for reusable, innovative TPMS structures [328]. Rollo and colleagues used SLS to manufacture TPU and graphene/MWCNT composites to develop nonporous designs and diamond TPMS porous structures for wearable sensing applications. They achieved linear and stable strain-resistance responses over a range of 0.1–2 N by optimizing piezoresistive performance through variations in porosity and thickness. Their results also show that the sensors (0.3 mm thickness) maintained sensitivity and reproducibility and are potentially applicable for prosthetics, consumer products, and health-monitoring systems [329]. Su and others used VPP and electrostatic self-assembly to create SiCw@MXene/SiOC gyroid TPMS metastructures for terahertz EMI shielding. The optimized gyroid structures have shown a strong electron-to-heat conversion capability, low thermal conductivity, and an excellent EMI shielding efficiency of 58.6 to 66.4 dB in the range of 0.2–1.6 THz. Their research demonstrates the multifunctional potential of 3D printed TPMS metastructures for electrical protection in harsh environments [330]. Leveraging the DLP, Saadi et al. created MWCNT/polymer nanocomposites to develop self-sensing gyroid TPMS lattices. Their optimized resin demonstrated distinct strain and damage sensing during deformation and collapse, enabling ultralow percolation of 0.01 phr (parts per hundred resin) MWCNTs with improved piezoresistive sensitivity. Their work on wearable sensors and structural health monitoring shows the potential of DLP printed multifunctional TPMS structures [331].

## 7. Limitations and Research Opportunities

Despite the extensive progress in the design and additive manufacturing of TPMS-based structures, several limitations still constrain their transition from research prototypes to practical engineering applications. One major challenge is reproducibility across length scales. Although TPMS unit cells are mathematically defined, actual fabrication often introduces geometric deviations and surface roughness that influence mechanical and transport properties. Fatigue reliability under cyclic and multi-axial loading remains insufficiently characterized, as shown by Dyer et al. [217]. Even high-quality metallic TPMS structures exhibit scatter in fatigue life depending on surface finish and internal defects. Manufacturability and scalability represent another key limitation. Thin-walled TPMS architectures fabricated through PBF or VPP are highly sensitive to process parameters, and powder entrapment in closed channels continues to impede post-processing and quality assurance [88,129,332,333,334]. Multi-material and graded-material fabrication, although conceptually promising for functionally graded TPMS, is limited by interfacial bonding strength, thermal mismatch stresses, and insufficient printer resolution [335,336,337]. Standardization and metrology frameworks are still emerging, hindering consistent comparison across different studies [338,339]. For biomedical applications, biocompatibility and regulatory validation remain challenging [340]. Zhou et al. [341] reported improved permeability and cytocompatibility in Ti64-5Cu alloy TPMS scaffolds fabricated by PBF. However, long-term in vivo data and regulatory processes remain underdeveloped. A recent review by Wakjira et al. [342] further highlights the need for standardized biological testing, surface modification, and multi-scale modeling for bone-related TPMS scaffolds. Finally, lifecycle sustainability and cost analyses are seldom addressed, despite their importance for industrial viability [343]. Future research should therefore focus on (i) establishing reproducible design to manufacture workflows, (ii) developing standardized testing and certification protocols, (iii) advancing AI-assisted process monitoring for defect prediction [344,345], and (iv) expanding sustainable and recyclable material options [346]. Overcoming these limitations will enhance the progression of TPMS metamaterials for high-volume fabrication, practical deployment, and compliance with regulatory standards.

## 8. Conclusions

TPMS structures have gained significant attention as material systems that integrate precise geometric definition with diverse functional performance. The development of additive manufacturing has made it possible to create these complex geometries with consistent accuracy across a wide range of materials and scales. Through controlled porosity and continuous topology, TPMS lattices can achieve outstanding combinations of mechanical strength, energy absorption, and thermal regulation, making them highly suitable for biomedical, structural, and thermal applications. Their periodic and interconnected geometries also allow precise control over stiffness, permeability, and surface area, enabling optimized performance across multiple functions. Future investigations should focus on incorporating TPMS structures into emerging areas such as electrochemical energy systems (battery and fuel-cell electrodes), catalytic supports, and bio-inspired materials. Since additive manufacturing continues to develop, particularly in multi-material printing and in situ process monitoring, the potential of TPMS structures will continue to grow. These advancements will further reinforce their role in the development of sustainable, high-performance, and multifunctional engineering applications.

## Figures and Tables

**Table 1:** Implicit functions and symbol definitions for commonly used TPMS geometries.

Unit Name	Mathematical Expression	3D Models
Primitive (P)	*f*(*x*,*y*,*z*) = cos(*ω_x_x*) + cos(*ω_y_y*) + cos(*ω_z_z*) = *C*	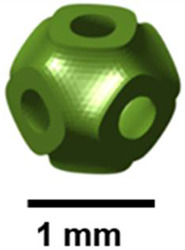
Gyroid (G)	*f*(*x*,*y*,*z*) = sin(*ω_x_x*) cos(*ω_y_y*) + sin(*ω_z_z*) cos(*ω_x_x*) + sin(*ω_y_y*) cos(*ω_z_z*) = *C*	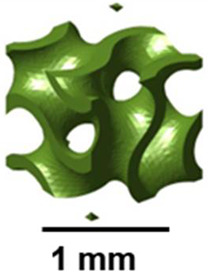
Diamond (D)	*f*(*x*,*y*,*z*) = cos(*ω_x_x*) cos(*ω_y_y*) cos(*ω_z_z*) − sin(*ω_x_x*) sin(*ω_y_y*) sin(*ω_z_z*) = *C*	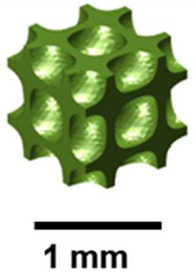
Neovius (N)	*f*(*x*,*y*,*z*) = 3[cos(*ω_x_x*) + cos(*ω_y_y*) + cos(*ω_z_z*)] + 4cos(*ω_x_x*) cos(*ω_y_y*) cos(*ω_z_z*) = *C*	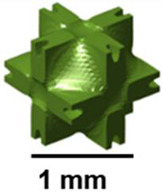
IWP	*f*(*x*,*y*,*z*) = 2[cos(*ω_x_x*) cos(*ω_y_y*) + cos(*ω_y_y*) cos(*ω_z_z*) + cos(*ω_z_z*) cos(*ω_x_x*)] − [cos(2*ω_x_x*) + cos(2*ω_y_y*) + cos(2*ω_z_z*)] = *C*	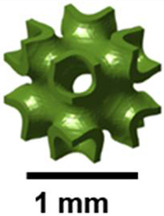
FRD	*f*(*x*,*y*,*z*) = 4cos(*ω_x_x*) cos(*ω_y_y*) cos(*ω_z_z*) − [cos(2*ω_x_x*) cos(2*ω_y_y*) + cos(2*ω_y_y*) cos(2*ω_z_z*) + cos(2*ω_z_z*) cos(2*ω_x_x*)] = *C*	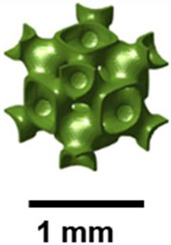
I_2_Y	*f*(*x*,*y*,*z*) = 2[sin(2*ω_x_x*) cos(*ω_y_y*) sin(*ω_y_z*) + sin(*ω_x_x*) sin(2*ω_y_y*) cos(*ω_z_z*) + cos(*ω_x_x*) sin(*ω_y_y*) sin(2*ω_z_z*)] + cos(2*ω_x_x*) cos(2*ω_y_y*) + cos(2*ω_y_y*) cos(2*ω_z_z*) + cos(2*ω_x_x*) cos(2*ω_z_z*) = *C*	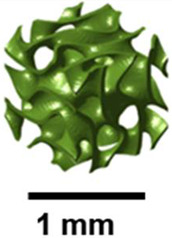
PMY	*f*(*x*,*y*,*z*) = 2cos(*ω_x_x*) cos(*ω_y_y*) cos(*ω_z_z*) + sin(2*ω_x_x*) sin(*ω_y_y*) + sin(*ω_x_x*) sin(2*ω_z_z*) + sin(2*ω_y_y*) sin(*ω_z_z*) = *C*	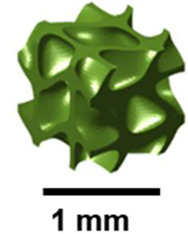
FKS	*f*(*x*,*y*,*z*) = cos(2*ω_x_x*) sin(*ω_y_y*) cos(*ω_z_z*) + cos(*ω_x_x*) cos(2*ω_y_y*) sin (*ω_z_z*) + sin(*ω_x_x*) cos(*ω_y_y*) cos(2*ω_z_z*) = *C*	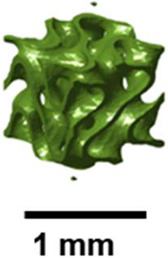

**Table 2:** A summary of properties and applications of TPMS structures printed with different materials using various AM techniques.

TPMS Geometry	Material & AM Process	Relative Density/Porosity	Key Mechanical Properties	Energy-Absorption/Efficiency	Application	References
Sheet-based Primitive, Diamond, and Gyroid; cubic unit-cell arrangement	316L stainless steel; SLM	ρ* = 0.15–0.40 (varied via wall thickness); unit-cell size 6–10 mm	E and σᵧ increase with ρ*; Diamond exhibits the highest stiffness and plateau stress; Diamond and Gyroid show stable collapse under quasi-static compression	Diamond sheet achieves the highest absorbed energy and SEA up to mid-strain; densification strain εᴰ 0.45–0.55	Sheet-based metallic TPMS identified as effective lightweight energy-absorbing cores; performance exceeds BCC lattices of similar density	[31]
Gyroid & Diamond TPMS lattices	Ti-6Al-4V; SLM	Porosity 80–95%; pore sizes 560–1600 μm (Gyroid) and 480–1450 μm (Diamond)	Compressive modulus 0.12–1.25 GPa (trabecular-bone range); strength increases with decreasing porosity; stable non-brittle deformation	-	Bone-implant scaffolds; designed to match bone stiffness while maintaining high permeability	[66]
Diamond & Gyroid TPMS	Ti-6Al-4V; SLM	Porosity up to 71% across various cell morphologies	Elastic modulus 3.2 GPa; yield strength 92–276 MPa; permeability 0.05–6.1 × 10^−9^ m^2^; fatigue limit up to 60% σᵧ	-	Load-bearing bone scaffolds require bone-like stiffness and long fatigue life	[12]
Graded TPMS scaffolds (P-surface & D-surface)	PolyJet AM (photopolymer resin)	Linearly graded porosity along the build direction, varied layer-by-layer	Intermediate mechanical response between low- and high-density regions; smoother stress–strain behavior than uniform scaffolds	-	Tissue-engineering scaffolds with spatially graded porosity	[67]
Multi-morphology TPMS combining P and G surfaces	Ti-6Al-4V; DMLS	Porosity not explicitly quantified; morphology varies spatially due to hybrid topology	Combination of lower modulus and higher yield strength; progressive collapse with delayed failure	-	Ti-6Al-4V scaffolds; hybrid topology mimics bone’s gradient stiffness.	[68]
Double Gyroid (sheet-based TPMS)	Al-Si10-Mg; SLM	Several designed cell sizes and nominal relative densities (RD)	Heat-treated structures show stable plateau behavior and suppress brittle failure	SEA 16 MJ·m^−3^ up to 50% strain for heat-treated samples	Lightweight TPMS cores for crash and impact mitigation; heat treatment improves ductility and EA performance	[20]
TPMS-modified BCC (BT10, BT20, BT30).	Ti-6Al-4V; SLM	Volume fractions 10%, 20%, 30% corresponding to BT variants	Higher post-yield capacity and improved failure modes than classical BCC; strength increases with RD	BT30 achieves 33.16 MJ·m^−3^, 3 times higher than conventional BCC (B30) at identical density	Crash-protection components; TPMS curvature improves SEA and collapse stability	[69]
Sheet and strut-based Gyroid (uniform & graded)	Photopolymer resin; SLA	Multiple designed porosity levels; uniform and graded density profiles	Sheet-based gyroid: more isotropic and stiffer than strut gyroid; graded structures deform more smoothly	EA behavior assessed qualitatively via deformation behavior	Suitable for crash-mitigation and impact-moderation applications	[70]
Schwarz Primitive (P), Schwarz Diamond (D), Gyroid (G), Neovius (N)	PLA; FFF	Designed RD values 10%, 20%, 30%	Schwarz D highest strength; Gyroid/Neovius intermediate; SP lowest; stiffness increases with RD	Results focused on stress–strain shape and qualitative collapse behavior	Low-cost polymer TPMS cores for moderate load applications	[71]
Cylindrical Diamond TPMS	316L stainless steel; SLM	Designed RD 20%; actual mass-based RD 19.6–20.3%; porosity 80%	Bending-dominated (nᵣ = 1): smoother plateau; stretch-dominated (nᵣ ≥ 1.5): oscillatory collapse	SEA to densification: 7.67–17.53 MJ·m^−3^ (highest for D9,1,0.64)	Metal TPMS cylinders for crash/impact mitigation	[33]
Gyroid, Schwarz Diamond, Neovius, D-Prime TPMS	PLA; FDM	Fill factor 17.5–32%; measured densities 0.20–0.36 g·cm^−3^	Compressive strength 2.64–7.43 MPa; yield strain 4–15%; densification strain 46–54%	A30 (specific energy absorption at 30% strain): 0.65–1.95 MJ·m^−3^, increasing with fill factor and geometric complexity	TPMS energy absorbers for the aerospace industry	[72]
FRD (Fischer–Koch S surface) and Neovius (sheet-based TPMS)	SS316L; SLM	Designed RD = 30%; measured RD = 27.34% (Neovius) and 27.75% (FRD) (7–9% deviation due to defects)	Neovius shows higher plateau stress under both quasi-static and dynamic loading (102 MPa and 118 MPa). FRD: 94.15 MPa quasi-static, 20% higher under dynamic loading. Both structures exhibit enhanced strength at high strain rates (strain-hardening effect)	SEA (specific energy absorption): Neovius = 22.11 J/g (quasi-static) and 24.8 J/g (dynamic). FRD shows a 14% SEA increase under dynamic loading. Both geometries absorb more energy at high deformation rates	Demonstrates the effect of strain rate sensitivity on TPMS-based metallic lattices; relevant for impact mitigation and dynamic loading applications (automotive, aerospace, defense)	[56]

**Table 3:** A summary of different materials used for printing several types of TPMS structures.

Process	Materials	TPMS	Design	Simulation	References
LCD	eResin-PLA	D	Rhino 7	-	[110]
SLA	Ti6Al4V powder	G	MSLattice	-	[111]
SLA	White resin	P, G	MathMod	-	[112]
SLA	Standard white, BioMed Amber resins	G	nTopology	nTopology	[113]
SLA	Dental LT clear resin	P, G	Python	-	[98]
MSLA	Clear resin	G	Taguchi L9, nTopology, Fusion 360	-	[114]
SLM	MetcoAddTM 316L-A powder	G	nTopology	-	[115]
SLA	UV resin	P, G	MathMod	-	[112]
SLA	UV resin	P	Solidworks	Abaqus	[116]
SLA	CaCO3 & Resin	G	Fusion 360	Autodesk	[117]
SLA	Photopolymer	P, G, IWP, Split P	FLatt Pack	ANSYS Fluent	[118]
DLP	Photopolymer	G	nTopology	ANSYS CFX MATLAB	[119]
DLP	Photopolymer	G	nTopology	nTopology	[120]
DLP	Slurry	G, D, L	nTopology	-	[121]
DLP	Acrylic Resin	G, D	-	Moldex3D	[122]
DLP	Flexible Resin	P, G	Marching Cubes	Abaqus	[123]
SLM	Ti-6Al-4V	G, Stochastic Voronoi	nTopology	-	[124]
L-PBF	Ti Alloy Ti64	G, Stochastic Voronoi	nTopology	-	[93]
PBF	AlSi10Mg	G, IWP	in-house script	Abaqus	[125]
PBF-LB	Atomized 316L SS	G	FLatt Pack	Abaqus	[126]
L-PBF	Ti-6Al-4V	D, G, P	nTopology	nTopology	[127]
PBF-LB	AlSi10Mg	G	nTopology	-	[128]
E-PBF	Ti-6Al-4V	G	Wolfram Mathematica	ANSYS	[129]
L-PBF	TiCN-IN718	D, G, P	MSLattice	ANSYS	[130]
PBF	AlSi10Mg	G, D	SolidWorks	ANSYS	[131]
PBF	AlSi10Mg	G	SpaceClaim, nTopology	ANSYS	[132]
L-PBF	NiTi	G	MATLAB	Abaqus/Explicit	[133]
L-PBF	Ti-6Al-4V	G, P	Grasshopper	Abaqus, COMSOL	[134]
SLS	PA 12	G	Rhino 7, Grasshopper		[135]
SLS	PA1102	D, G, IWP	MSlattice	Abaqus	[136]
SLS	PA 2200	P, IWP, N	Surface Evolver, SolidWorks	Abaqus, PolyUMod	[90]
SLS	PA 2202	P, G, FK, CLP	-	-	[137]
SLS	PLA/pFe	IWP	MSLattice	-	[138]
FDM	ePA-GF	D, G, P	-	-	[139]
FDM	PLA + CF	P, G, D	Creo 8.0	-	[140]
FDM	PLA/GO	P, G	SolidWorks	-	[141]
FDM	PLA	D, G	MSlattice	Abaqus	[142]
FDM	PLA	G	nTopology	ANSYS	[143]
FDM	PLA	P, N, IWP	Matlab	ABAQUS/Explicit	[144]
FDM	MXene/MWCNTs with TPU	G	Rhinoceros 7	ABAQUS 6.11	[145]
FDM	PEGDA/GelMA hydrogel	G, D, IWP	MSLattice	ANSYS	[146]
FDM	PEEK/SiN	G	MSLattice	ABAQUS	[147]
FFF	PLA	G	nTopology	ANSYS	[143]
FFF	PLA	G	MS Lattice	ANSYS	[148]
LCD	LCD resin	G	MATLAB	ANSYS	[149]
FFF	PLA	P, G, D, S, IWP, PW	MS Lattice Rhinoceros	-	[84]
FFF	PEEK	G, D	MathMod	-	[32]
FFF	PLA	P	SolidWorks	NX Nastran 2019.1	[150]
FFF	PLA	P, G	PTC Creo Parametric 7.0	-	[151]
μLPBF	SS316L	P, G, D	Materialize Magics and MATLAB	Abaqus/Explicit 2017	[89,152]
LPBF	Ti-6Al-4V	G	Rhino7.0	-	[153]
LPBF	Ti-6A-l4V	G, D, FKS	Materialize Magics	Abaqus/CFD 6.14	[154]
LPBF	Ti-42Nb	IWP	MATLAB	Pam-Crash	[155]
LPBF	SS316L	P, G, D, IWP, FK	-	-	[96]
LPBF	CoCrMo	N, IWP	CTvox software	-	[156]
LPBF	Nylon Powder	ST-P	Mathematica 11.2	ANSYS Workbench	[157]
SLM	316LSS	D, G, P	-	ABAQUS/EXPLICIT	[158]
SLM	Ti-6A-l4V	G	Mathematica		[159]
SLM	Ti-6A-l4V	D	Matlab	Abaqus/Standard 2016	[160]
SLM	CpTi (α Ti)	G, D, I2Y, IWP	MATLAB	-	[161]
SLM	AlSi10Mg	G		-	[20]
SLM	AlSi10Mg	P, G, D, GD, GP, DP	MSLattice	-	[162]
SLM	Al alloy	P, G, S-D	-	ANSYS CFX	[163]
SLM	SS304L	P	SolidWorks	-	[164]
SLM	SS316L	G	MATLAB	ABAQUS/Standard 6.14	[165]
SLM	Ti-6Al-4V	P, G	MATLAB	ABAQUS/Explicit	[166]
SLM	Ti6Al4V	G, D	-	ANSYS Fluent	[167]
MLM	HS188	G	MATLAB	Dewesoft	[46]
MJT	ABS resin	P, D	MATLAB	ABAQUS	[67]
MJT	VisiJet M3 Crystal	P, G, D	-	ANSYS Fluent	[168]
BJ	Ti-6Al-4V	U-GLS, G-GLS	MSLattice	ABAQUS	[169]
BJ	Alumina ceramics	IWP, G, D, P	-	-	[170]
SLS, FDM	PA 2200, PLA	G, D, N, IWP	Rhinoceros 6	-	[171]
LCD	Photopolymer Ceramic Resin	G, Schwarz, D, L, N, SplitP	nTop	-	[172]
LCD	Photocuring ink	IWP, D, G, P	-	-	[173]
LCD	Photopolymer	D, G	SolidWorks	-	[174]
LCD	Sacrificial Resin	D, G, IWP	MSLattice	-	[175]
-	AlSi10Mg	P, G	MSLattice	STAR-CCM +	[176]
-	-	P, G, FKS	Rhinoceros 6	ANSYS Fluent	[177]
-	Al	D	MathMod	OpenFOAM	[178]
-	Steel	P, G, D, FRD, IWP	MS Lattice	Abaqus	[179]
-	AlSi10Mg	P, G, IWP	MSLattice	ANSYS Fluent	[180]

**Table 4:** Practical DfM guidelines for TPMS structures across major AM processes.

AM Techniques	Typical Minimum Printable Wall Thickness	Geometric Deviations	TPMS Specific Concerns	Practical Mitigation Strategies	References
Material Extrusion (FFF/FDM/MEX)	0.8–1.2 mm, constrained by nozzle diameter, bead width, and cooling limitations	Bead swelling, staircase stepping, warpage, and gaps between adjacent roads	Fragility of thin TPMS sheets; loss of curvature fidelity; anisotropic behavior from raster orientation	Follow TPMS curvature with perimeters; reduce print speed; optimize extrusion and cooling; anneal semi-crystalline polymers if required	[181,182]
Vat Photopolymerization (SLA/DLP/MSLA)	0.25–0.40 mm, limited by pixel size/laser spot and resin curing depth	Over polymerization, lateral light scattering, stair-stepping on shallow slopes, and shrinkage during post-curing	Resin entrapment in bicontinuous channels; suction cup delamination; difficulty draining enclosed TPMS porosity	Tilt orientation for drainage; add vent holes; adjust exposure energy; apply controlled UV post-curing to minimize shrinkage	[98,183,184]
Powder Bed Fusion Polymers (SLS/PA12)	0.5–0.8 mm for stable self-supporting PA12 TPMS sheets	Corner rounding, partial sintering, stair stepping, and spatial variability in surface roughness	Powder retention in TPMS networks increases resistance to flow and adds mass	Provide vertical escape vents; moderate laser power to prevent over-sintering; post-process via bead blasting or infiltration to improve surface quality	[24,185,186]
Powder Bed Fusion Metals (LPBF/SLM)	0.3–0.5 mm for Ti-6Al-4V and CoCr TPMS sheets	Down-facing roughness, partially fused powder, melt pool instability, and residual stresses	Powder trapped in internal channels; internal roughness reduces permeability and increases pressure drop; geometric distortion during cooling	Add powder escape paths; orient structures for gravity-assisted drainage; apply contour plus hatch for thin sheets; use stress relief heat treatments	[12,187]
Electron Beam PBF (E-PBF)	0.8–1.2 mm required for stable Ti-6Al-4V thin walls; thinner sections (<0.8 mm) show severe roughness and geometric inaccuracy	Pronounced surface roughness and melt pool-induced rippling; orientation-dependent distortion of thin walls; reduced feature resolution compared with LPBF	Internal roughness and limited resolution degrade TPMS curvature fidelity; powder entrapment becomes problematic in narrow TPMS channels; down-facing regions are prone to geometric drift	Use thicker TPMS sheets (≥1 mm) to ensure stability; orient structures for gravity-assisted powder evacuation; include drain/vent pathways; apply ultrasonic de powdering (E-PBF); use electropolishing or surface finishing to reduce roughness	[188,189,190]
Material jetting (MJT/Polyjet)	0.6–1.0 mm for robust free-standing TPMS sheets	Edge chipping, droplet coalescence, and dimensional bias between matte and glossy surfaces	Support material entrapment in TPMS channels; increased mass and hindered drainage	Prefer matte orientation; add drain or clean out windows; ensure line of sight to internal channels; validate support removal using cleaning tests	[106,191]
Binder Jetting (BJ-Metals/Ceramics)	1.5–2.0 mm minimum green body wall thickness for safe handling, depowdering, and sintering	Significant shrinkage (5–20%), warpage during debinding, and distortion from nonuniform green density	Binder/powder accumulation in TPMS channels; cracking at thick junction regions; reduction in designed porosity after sintering	Maintain uniform wall thickness; incorporate vent/drain channels; apply controlled debinding sintering ramps; use geometry compensation strategies	[192,193,194,195]
Directed Energy Deposition (DED)	≥1.5–2.0 mm for stable walls due to large melt pool width; thin walls (<1.5 mm) exhibit poor stability, waviness, and dimensional inaccuracy.	Melt pool oversize leading to wide beads; ripple formation and waviness along deposition tracks; reduced resolution of fine features; thermal gradients contribute to distortion and residual stress	Poor resolution limits fabrication of thin TPMS sheets; excessive bead width blurs curvature fidelity; internal TPMS channels cannot be reliably formed; surface roughness degrades mechanical uniformity	Restrict TPMS designs to coarse features (≥2 mm); use optimized deposition paths to reduce waviness; apply surface machining or grinding for smooth final geometry; manage preheat/interpass temperature to reduce thermal distortion	[196,197]

**Table 5:** Critical AM parameters influencing TPMS dimensional accuracy, mechanical behavior, and reproducibility.

Parameter	Recommended Practice	Effect on Dimensional Accuracy	Effect on Mechanical Properties	Effect on Reproducibility	References
Layer thickness (VPP/LPBF)	Use smaller layer heights to capture curved features more accurately	Reduces staircase effects and improves surface precision, especially in regions of high curvature	Produces more uniform walls, which can result in slightly improved strength	Provides more consistent geometry across multiple prints	[6,114,198,199,200]
Energy input (laser power, UV exposure)	Adjust laser/UV energy to achieve sufficient curing or melting without overexposure	Enhance wall resolution and reduce voids or incomplete fusion	Improves curing depth in VPP and stabilizes melt-pool formation in LPBF, leading to better mechanical stability	Decreases the occurrence of process-induced defects, resulting in more predictable outputs	[198,199,201]
Hatch spacing (LPBF)	Select a smaller hatch spacing to improve overlap between melt tracks	Minimizes lack of fusion pores and improves continuity in thin TPMS sheet structures	Produces more uniform load-bearing walls, which enhances stiffness and compressive strength	Improves repeatability of printed components as the melt-pool behavior becomes more stable	[6,200]
Structure parameters (TPMS geometry)	Choose appropriate wall thickness and unit cell size based on the accuracy limits of the printing process	Determines the smallest printable features and reduces geometric deviation in thin-walled TPMSs	Strongly influences stiffness, deformation behavior, and energy absorption capacity	More consistent structural design contributes to stable mechanical responses across builds	[200]
Feedstock & thermal conditions (FFF/MEX)	Optimize extrusion temperature, printing speed, and cooling rate	Improves dimensional uniformity by minimizing thermal distortion and material shrinkage	Enhance structural integrity and increase mechanical strength by reducing air gaps or weak bonds	Stable thermal control during printing results in more reproducible mechanical performance	[141,202]
Post-curing (VPP)	Apply sufficient post-curing under UV light to complete polymer conversion	Improves surface stability and reduces minor dimensional variations that arise from incomplete polymerization	Increases crosslink density and improves modulus, yield strength, and overall robustness	Reduces batch-to-batch variations originating from differences in polymer conversion	[198,201]
Environmental control (VPP/FFF)	Maintain controlled temperature and humidity throughout the printing process	Reduces warping, dimensional drift, and layer misalignment caused by environmental fluctuations	Promotes consistent interlayer bonding, resulting in more stable mechanical properties	Provides a more predictable printing environment that improves overall reproducibility	[202]
Powder quality (LPBF)	Use fine particle size distribution and limit the ratio of recycled powder in each build	Improves melt-pool stability and geometric precision, particularly in thin TPMS walls	Leads to more uniform mechanical properties by reducing defects associated with irregular powder morphology	Ensures consistent powder behavior across builds, improving dimensional and mechanical repeatability	[6,200]

## Data Availability

No new data were created or analyzed in this study. Data sharing is not applicable to this article.

## References

[1] (2021). Additive Manufacturing—General Principles—Terminology.

[2] Gibson I., Rosen D., Stucker B. (2015). Additive Manufacturing Technologies: 3D Printing, Rapid Prototyping, and Direct Digital Manufacturing.

[3] Thompson M.K., Moroni G., Vaneker T., Fadel G., Campbell R.I., Gibson I., Bernard A., Schulz J., Graf P., Ahuja B. (2016). Design for Additive Manufacturing: Trends, opportunities, considerations, and constraints. CIRP Ann..

[4] Ngo T.D., Kashani A., Imbalzano G., Nguyen K.T., Hui D. (2018). Additive manufacturing (3D printing): A review of materials, methods, applications and challenges. Compos. Part B Eng..

[5] Frazier W.E. (2014). Metal additive manufacturing: A review. J. Mater. Eng. Perform..

[6] DebRoy T., Wei H.L., Zuback J.S., Mukherjee T., Elmer J.W., Milewski J.O., Beese A.M., Wilson-Heid A., De A., Zhang W. (2018). Additive manufacturing of metallic components–process, structure and properties. Prog. Mater. Sci..

[7] (2015). Standard Terminology for Additive Manufacturing—General Principles—Terminology.

[8] Hossain M.S., Rabi S.N., Mohammad S., Cook K., Chowdhury F., Nilufar S. (2025). Investigation of Thermomechanical Properties of Hollow Glass Microballoon-Filled Composite Materials Developed by Additive Manufacturing with Machine Learning Validation. Polymers.

[9] Gu D. (2015). Laser Additive Manufacturing of High-Performance Materials.

[10] Gibson I., Rosen D., Stucker B., Khorasani M., Rosen D., Stucker B., Khorasani M. (2021). Additive Manufacturing Technologies.

[11] Schoen A.H. Infinite Periodic Minimal Surfaces Without Self-Intersections. https://ntrs.nasa.gov/citations/19700020472.

[12] Bobbert F., Lietaert K., Eftekhari A.A., Pouran B., Ahmadi S., Weinans H., Zadpoor A. (2017). Additively manufactured metallic porous biomaterials based on minimal surfaces: A unique combination of topological, mechanical, and mass transport properties. Acta Biomater..

[13] Al-Ketan O., Rowshan R., Al-Rub R.K.A. (2018). Topology-mechanical property relationship of 3D printed strut, skeletal, and sheet based periodic metallic cellular materials. Addit. Manuf..

[14] Maskery I., Aboulkhair N., Aremu A., Tuck C., Ashcroft I., Wildman R.D., Hague R. (2016). A mechanical property evaluation of graded density Al-Si10-Mg lattice structures manufactured by selective laser melting. Mater. Sci. Eng. A.

[15] Vijayavenkataraman S., Kuan L.Y., Lu W.F. (2020). 3D-printed ceramic triply periodic minimal surface structures for design of functionally graded bone implants. Mater. Des..

[16] Zhang Y., Zhang J., Zhao X., Li Y., Che S., Yang W., Han L. (2022). Mechanical behaviors regulation of triply periodic minimal surface structures with crystal twinning. Addit. Manuf..

[17] Hyde S., Blum Z., Landh T., Lidin S., Ninham B., Andersson S., Larsson K. (1996). The Language of Shape: The Role of Curvature in Condensed Matter: Physics, Chemistry and Biology.

[18] Al-Ketan O., Abu Al-Rub R.K. (2019). Multifunctional mechanical metamaterials based on triply periodic minimal surface lattices. Adv. Eng. Mater..

[19] Karcher H. (1989). The triply periodic minimal surfaces of Alan Schoen and their constant mean curvature companions. Manuscripta Math..

[20] Maskery I., Aboulkhair N.T., Aremu A.O., Tuck C., Ashcroft I.A. (2017). Compressive failure modes and energy absorption in additively manufactured double gyroid lattices. Addit. Manuf..

[21] Schwarz H.A. (1972). Gesammelte Mathematische Abhandlungen.

[22] Guo X., Ding J., Li X., Qu S., Fuh J.Y.H., Lu W.F., Song X., Zhai W. (2023). Interpenetrating phase composites with 3D printed triply periodic minimal surface (TPMS) lattice structures. Compos. B Eng..

[23] Cheung S., Kang J., Lin Y., Goodson K.E., Asheghi M., Gu X.W. (2025). Triply periodic minimal surfaces for thermo-mechanical protection. Sci. Rep..

[24] Maskery I., Sturm L., Aremu A.O., Panesar A., Williams C.B., Tuck C.J., Wildman R.D., Ashcroft I.A., Hague R.J.M. (2018). Insights into the mechanical properties of several triply periodic minimal surface lattice structures made by polymer additive manufacturing. Polymer.

[25] Mulhi A., Dehgahi S., Waghmare P., Qureshi A. (2023). Dimensional assessment of uniformly periodic porosity primitive TPMS lattices using additive manufacturing laser powder bed fusion technique. Int. J. Adv. Manuf. Tech..

[26] Jagadeesh B., Duraiselvam M. (2023). Investigations on the compressive behaviour of novel cell size graded primitive lattice structure produced by Metal Additive Manufacturing. Mater. Lett..

[27] Ebrahimzadeh Dehaghani A., Javanbakht Z., Barzan M., Lloyd D.G., Feih S. (2024). Multifunctional design of triply periodic minimal surface structures for temporary pediatric fixation devices. Adv. Eng. Mater..

[28] Almomani A., Mourad A.H.I. (2023). The fracture toughness of Schwarz Primitive triply periodic minimal surface lattice. Theor. Appl. Fract. Mec..

[29] Lai M., Kulak A.N., Law D., Zhang Z., Meldrum F.C., Riley D.J. (2007). Profiting from nature: Macroporous copper with superior mechanical properties. Chem. Commun..

[30] Shah G.J., Nazir A., Lin S.C., Jeng J.Y. (2022). Design for Additive Manufacturing and Investigation of Surface-Based Lattice Structures for Buckling Properties Using Experimental and Finite Element Methods. Materials.

[31] Zhang L., Feih S., Daynes S., Chang S., Wang M.Y., Wei J., Lu W.F. (2018). Energy absorption characteristics of metallic triply periodic minimal surface sheet structures under compressive loading. Addit. Manuf..

[32] Spece H., DeSantis P.M., Kurtz S.M. (2022). Development of an architecture-property model for triply periodic minimal surface structures and validation using material extrusion additive manufacturing with polyetheretherketone (PEEK). J. Mech. Behav. Biomed. Mater..

[33] Laskowska D., Szatkiewicz T., Bałasz B., Mitura K. (2023). Mechanical Properties and Energy Absorption Abilities of Diamond TPMS Cylindrical Structures Fabricated by Selective Laser Melting with 316L Stainless Steel. Materials.

[34] Giorleo L., Basu S., Piana E. (2025). Acoustic performances of triply periodic minimal surfaces fabricated by additive manufacturing: Effects of cell geometry, aspect ratio, and wall thickness. Addit. Manuf..

[35] Felix L.C., Gaál V., Woellner C.F., Rodrigues V., Galvao D.S. (2020). Mechanical Properties of Diamond Schwarzites: From Atomistic Models to 3D-Printed Structures. MRS Adv..

[36] Yeranee K., Rao Y., Xu C., Zhang Y., Su X. (2023). Turbulent flow heat transfer and thermal stress improvement of gas turbine blade trailing edge cooling with diamond-type TPMS structure. Aerospace.

[37] Galusha J.W., Richey L.R., Gardner J.S., Cha J.N., Bartl M.H. (2008). Discovery of a diamond-based photonic crystal structure in beetle scales. Phys. Rev. E—Stat. Nonlinear Soft Matter Phys..

[38] Große-Brauckmann K., Meinhard W. (1996). The gyroid is embedded and has constant mean curvature companions. Calc. Var. Partial. Differ. Equ..

[39] Caiazzo F., Alfieri V., Bujazha B.D. (2021). Additive manufacturing of biomorphic scaffolds for bone tissue engineering. Int. J. Adv. Manuf. Tech..

[40] Peng C.X., Tran P. (2020). Bioinspired functionally graded gyroid sandwich panel subjected to impulsive loadings. Compos. B Eng..

[41] Prussack B., Jentz I., Moreira T.A., Woolstenhulme N., Jesse C., Nellis G., Anderson M. (2024). Thermal and hydraulic performance of volumetrically heated triply periodic minimal surface heaters. Appl. Therm. Eng..

[42] Shaikh A., Saxena A., Griffis J., Shahed K., Manogharan G. (2024). Functionally graded TPMS gyroid structures for additive manufacturing of non-pneumatic tires. Mater. Sci. Addit. Man..

[43] Al-Ketan O., Lee D.W., Abu Al-Rub R.K. (2021). Mechanical properties of additively-manufactured sheet-based gyroidal stochastic cellular materials. Addit. Manuf..

[44] Vafaeefar M., Moerman K.M., Vaughan T.J. (2024). Experimental and computational analysis of energy absorption characteristics of three biomimetic lattice structures under compression. J. Mech. Behav. Biomed. Mater..

[45] Abueidda D.W., Elhebeary M., Shiang C.S., Pang S.Y., Abu Al-Rub R.K., Jasiuk I.M. (2019). Mechanical properties of 3D printed polymeric Gyroid cellular structures: Experimental and finite element study. Mater. Des..

[46] Simsek U., Arslan T., Kavas B., Gayir C.E., Sendur P. (2021). Parametric studies on vibration characteristics of triply periodic minimum surface sandwich lattice structures. Int. J. Adv. Manuf. Tech..

[47] Nath S.D., Nilufar S. (2022). Performance Evaluation of Sandwich Structures Printed by Vat Photopolymerization. Polymers.

[48] Corkery R.W., Tyrode E.C. (2017). On the colour of wing scales in butterflies: Iridescence and preferred orientation of single gyroid photonic crystals. Interface Focus.

[49] Viswanath A., Abueidda D.W., Modrek M., Khan K.A., Koric S., Al-Rub R.K.A. (2023). Gyroid-like metamaterials: Topology optimization and deep learning. arXiv.

[50] Huffman B., Youssef G. (2025). Additive manufacturing of triply periodic minimal surface structures with geometrical porosity. Prog. Addit. Manuf..

[51] Neovius E. (1883). Bestimmung Zweier Spezieller Periodischer Minimalflächen, Akad.

[52] Abueidda D.W., Elhebeary M., Shiang C.S., Abu Al-Rub R.K., Jasiuk I.M. (2020). Compression and buckling of microarchitectured Neovius-lattice. Extrem. Mech. Lett..

[53] Hur K., Hennig R.G., Wiesner U. (2017). Exploring Periodic Bicontinuous Cubic Network Structures with Complete Phononic Bandgaps. J. Phys. Chem. C.

[54] Khan K.A., Abu Al-Rub R.K. (2017). Time dependent response of architectured Neovius foams. Int. J. Mech. Sci..

[55] Khan S.Z., Masood S.H., Ibrahim E., Ahmad Z. (2019). Compressive behaviour of Neovius Triply Periodic Minimal Surface cellular structure manufactured by fused deposition modelling. Virtual Phys. Prototyp..

[56] AlMahri S., Santiago R., Lee D., Ramos H., Alabdouli H., Alteneiji M., Guan Z., Alves M. Investigation of the specific energy absorption of triply periodic minimal surfaces subjected to impact loading. Proceedings of the 13th International Conference on the Mechanical and Physical Behaviour of Materials Under Dynamic Loading.

[57] Silva T., Lu J.-Y., Abu Al-Rub R.K., Lee D.-W. (2024). Investigation on tailoring the width and central frequency of bandgaps of TPMS structures. Int. J. Mech. Mater. Des..

[58] Baghous N., Barsoum I., Al-Rub R.K.A. (2022). The effect of Lode parameter on the yield surface of Schoen’s IWP triply periodic minimal surface lattice. Mech. Mater..

[59] Khan K.A., Abu Al-Rub R.K. (2020). Viscoelastic properties of architected foams based on the Schoen IWP triply periodic minimal surface. Mech. Adv. Mater. Struct..

[60] Lu Y., Zhao W., Cui Z., Zhu H., Wu C. (2019). The anisotropic elastic behavior of the widely-used triply-periodic minimal surface based scaffolds. J. Mech. Behav. Biomed. Mater..

[61] Hassan I.M., Enab T.A., Fouda N., Eldesouky I. (2023). Design, fabrication, and evaluation of functionally graded triply periodic minimal surface structures fabricated by 3D printing. J. Braz. Soc. Mech. Sci..

[62] Xu Y.L., Pan H., Wang R.N., Du Q., Lu L. (2023). New families of triply periodic minimal surface-like shell lattices. Addit. Manuf..

[63] Kumar R., Ramkumar J., Balani K. (2024). Design and parametrization of TPMS lattice using computational and experimental approach. Eng. Res. Express.

[64] He X., Yang C., Zheng M., Zhang H., Du Y. (2025). A fast evaluation method for surface area, volume fraction, and hydraulic diameter of TPMS with different geometric characteristics. Proc. Inst. Mech. Eng. C J. Mech. Eng. Sci..

[65] Zhao M., Zhang D.Z., Liu F., Li Z.H., Ma Z.B., Ren Z.H. (2020). Mechanical and energy absorption characteristics of additively manufactured functionally graded sheet lattice structures with minimal surfaces. Int. J. Mech. Sci..

[66] Yan C., Hao L., Hussein A., Young P. (2015). Ti–6Al–4V triply periodic minimal surface structures for bone implants fabricated via selective laser melting. J. Mech. Behav. Biomed. Mater..

[67] Afshar M., Anaraki A.P., Montazerian H., Kadkhodapour J. (2016). Additive manufacturing and mechanical characterization of graded porosity scaffolds designed based on triply periodic minimal surface architectures. J. Mech. Behav. Biomed. Mater..

[68] Zhu L.Y., Li L., Shi J.P., Li Z.A., Yang J.Q. (2018). Mechanical characterization of 3D printed multi-morphology porous Ti6Al4V scaffolds based on triply periodic minimal surface architectures. Am. J. Transl. Res..

[69] Zhao M., Liu F., Fu G., Zhang D.Z., Zhang T., Zhou H. (2018). Improved mechanical properties and energy absorption of BCC lattice structures with triply periodic minimal surfaces fabricated by SLM. Materials.

[70] Li D., Liao W., Dai N., Xie Y.M. (2019). Comparison of mechanical properties and energy absorption of sheet-based and strut-based gyroid cellular structures with graded densities. Materials.

[71] Kladovasilakis N., Tsongas K., Tzetzis D. (2021). Mechanical and FEA-assisted characterization of fused filament fabricated triply periodic minimal surface structures. J. Compos. Sci..

[72] Sychov M., Lebedev L., Dyachenko S., Nefedova L. (2018). Mechanical properties of energy-absorbing structures with triply periodic minimal surface topology. Acta Astronaut..

[73] Saleh Alghamdi S., John S., Roy Choudhury N., Dutta N.K. (2021). Additive Manufacturing of Polymer Materials: Progress, Promise and Challenges. Polymers.

[74] Chiulan I., Frone A.N., Brandabur C., Panaitescu D.M. (2017). Recent Advances in 3D Printing of Aliphatic Polyesters. Bioengineering.

[75] Placone J.K., Engler A.J. (2018). Recent Advances in Extrusion-Based 3D Printing for Biomedical Applications. Adv. Healthc. Mater..

[76] Luo C., Wang X., Migler K.B., Seppala J.E. (2020). Upper bound of feed rates in thermoplastic material extrusion additive manufacturing(☆). Addit. Manuf..

[77] Gonzalez-Gutierrez J., Cano S., Schuschnigg S., Kukla C., Sapkota J., Holzer C. (2018). Additive Manufacturing of Metallic and Ceramic Components by the Material Extrusion of Highly-Filled Polymers: A Review and Future Perspectives. Materials.

[78] Liu Y., Chou T.W. (2020). Additive manufacturing of multidirectional preforms and composites: From three-dimensional to four-dimensional. Mater. Today Adv..

[79] Penumakala P.K., Santo J., Thomas A. (2020). A critical review on the fused deposition modeling of thermoplastic polymer composites. Compos. B Eng..

[80] Daminabo S.C., Goel S., Grammatikos S.A., Nezhad H.Y., Thakur V.K. (2020). Fused deposition modeling-based additive manufacturing (3D printing): Techniques for polymer material systems. Mater. Today Chem..

[81] Sinha S.K., Sadasivuni K.K., Deshmukh K., Almaadeed M.A. (2020). Additive manufacturing (AM) of medical devices and scaffolds for tissue engineering based on 3D and 4D printing. 3D and 4D Printing of Polymer Nanocomposite Materials: Processes, Applications, and Challenges.

[82] Al Hashimi N.S., Soman S.S., Govindharaj M., Vijayavenkataraman S. (2022). 3D printing of complex architected metamaterial structures by simple material extrusion for bone tissue engineering. Mater. Today Commun..

[83] Sabahi N., Farajzadeh E., Roohani I., Wang C.H., Li X.P. (2024). Material extrusion 3D printing of polyether-ether-ketone scaffolds based on triply periodic minimal surface designs: A numerical and experimental investigation. Appl. Mater. Today.

[84] Wang X., Gao T., Shi C., Zhou Y., Li Z., Wang Z. (2023). Effect of geometric configuration on compression behavior of 3D-printed polymeric triply periodic minimal surface sheets. Mech. Adv. Mater. Struct..

[85] Desole M.P., Gisario A., Barletta M. (2024). Energy absorption of PLA-based metamaterials manufactured by material extrusion: Dynamic loads and shape recovery. Int. J. Adv. Manuf. Tech..

[86] Zocca A., Gomes C.M., Mühler T., Günster J. (2014). Powder-Bed Stabilization for Powder-Based Additive Manufacturing. Adv. Mech. Eng..

[87] Roy N.K., Behera D., Dibua O.G., Foong C.S., Cullinan M.A. (2019). A novel microscale selective laser sintering (mu-SLS) process for the fabrication of microelectronic parts. Microsyst. Nanoeng..

[88] Khrapov D., Paveleva A., Kozadayeva M., Evsevleev S., Mishurova T., Bruno G., Surmenev R., Koptyug A., Surmeneva M. (2023). Trapped powder removal from sheet-based porous structures based on triply periodic minimal surfaces fabricated by electron beam powder bed fusion. Mater. Sci. Eng. A.

[89] Qu S., Ding J., Song X. (2021). Achieving Triply Periodic Minimal Surface Thin-Walled Structures by Micro Laser Powder Bed Fusion Process. Micromachines.

[90] Abueidda D.W., Bakir M., Abu Al-Rub R.K., Bergström J.S., Sobh N.A., Jasiuk I. (2017). Mechanical properties of 3D printed polymeric cellular materials with triply periodic minimal surface architectures. Mater. Des..

[91] Alsalla H., Hao L., Smith C. (2016). Fracture toughness and tensile strength of 316L stainless steel cellular lattice structures manufactured using the selective laser melting technique. Mater. Sci. Eng. A.

[92] Hussain S., Alagha A.N., Haidemenopoulos G.N., Zaki W. (2023). Microstructural and surface analysis of NiTi TPMS lattice sections fabricated by laser powder bed fusion. J. Manuf. Process..

[93] Araya M., Jaskari M., Rautio T., Guillén T., Järvenpää A. (2024). Assessing the compressive and tensile properties of TPMS-Gyroid and stochastic Ti64 lattice structures: A study on laser powder bed fusion manufacturing for biomedical implants. J. Sci. Adv. Mater. Devices.

[94] Tilton M., Borjali A., Griffis J.C., Varadarajan K.M., Manogharan G.P. (2023). Fatigue properties of Ti-6Al-4V TPMS scaffolds fabricated via laser powder bed fusion. Manuf. Lett..

[95] Yang K., Tang D.N., Tang H.B. (2024). Fabrication characterization and compression failure analysis of Ni-based alloy ABD-900AM TMPS structures via laser powder bed fusion. J. Manuf. Process..

[96] AlMahri S., Santiago R., Lee D.-W., Ramos H., Alabdouli H., Alteneiji M., Guan Z., Cantwell W., Alves M. (2021). Evaluation of the dynamic response of triply periodic minimal surfaces subjected to high strain-rate compression. Addit. Manuf..

[97] Santoliquido O., Colonabo P., Ortona A. (2019). Additive Manufacturing of ceramic components by Digital Light Processing: A comparison between the “bottom-up” and the “top-down” approaches. J. Eur. Ceram. Soc..

[98] Gabrieli R., Wenger R., Mazza M., Verné E., Baino F. (2024). Design, Stereolithographic 3D Printing, and Characterization of TPMS Scaffolds. Materials.

[99] Liang Y.J., He H.Y., Yin J., Liu Y.J., Huang J.Z., Wu Z.G., Zhai Y., Hui D.V., Yan L.W. (2024). Energy absorption of gradient triply periodic minimal surface structure manufactured by stereolithography. Rev. Adv. Mater. Sci..

[100] Zhang J., Hu Q., Wang S., Tao J., Gou M. (2020). Digital Light Processing Based Three-dimensional Printing for Medical Applications. Int. J. Bioprint.

[101] Sun C., Wang Y., McMurtrey M.D., Jerred N.D., Liou F., Li J. (2021). Additive manufacturing for energy: A review. Appl. Energy.

[102] Roohani I., Entezari A., Zreiqat H. (2023). Liquid crystal display technique (LCD) for high resolution 3D printing of triply periodic minimal surface lattices bioceramics. Addit. Manuf..

[103] Chen B., Sun X., Liu D., Tian H., Gao J. (2023). A novel method combining VAT photopolymerization and casting for the fabrication of biodegradable Zn-1Mg scaffolds with triply periodic minimal surface. J. Mech. Behav. Biomed. Mater..

[104] Hua S.-B., Yuan X., Wu J.-M., Su J., Cheng L.-J., Zheng W., Pan M.-Z., Xiao J., Shi Y.-S. (2022). Digital light processing porous TPMS structural HA & akermanite bioceramics with optimized performance for cancellous bone repair. Ceram. Int..

[105] Nath S.D., Nilufar S. (2020). An overview of additive manufacturing of polymers and associated composites. Polymers.

[106] Dilag J., Chen T., Li S., Bateman S.A. (2019). Design and direct additive manufacturing of three-dimensional surface micro-structures using material jetting technologies. Addit. Manuf..

[107] Shen X.C., Naguib H.E. (2019). A robust ink deposition system for binder jetting and material jetting. Addit. Manuf..

[108] Sathishkumar N., Vivekanandan N., Balamurugan L., Arunkumar N., Ahamed I. (2020). Mechanical Properties of Triply Periodic Minimal Surface based lattices made by Polyjet Printing. Mater. Today Proc..

[109] Shilova O.A. (2020). Fractals, morphogenesis and triply periodic minimal surfaces in sol-gel-derived thin films. J. Sol-Gel Sci. Technol..

[110] Zeng C., Hu J., Liu L., Zhao W., Xin X., Song X., Liu Y., Leng J. (2025). Mechanical properties of diamond-type triply periodic minimal surface structures fabricated by photo-curing 3D printing. Compos. Struct..

[111] Zheng X.X., Duan F., Song Z.Y., Mo H.B., Li Z.H., Song Y.H., Su Y.C., Wang X.Y. (2022). A TMPS-designed personalized mandibular scaffolds with optimized SLA parameters and mechanical properties. Front. Mater..

[112] Yu S.X., Sun J.X., Bai J.M. (2019). Investigation of functionally graded TPMS structures fabricated by additive manufacturing. Mater. Des..

[113] Araya M., Murillo J., Vindas R., Guillén T. (2024). Compressive behavior of SLA open-cell lattices: A comparison between triply periodic minimal surface gyroid and stochastic structures for artificial bone. Materialia.

[114] Ramírez Rodríguez C.A., Narváez Tovar C.A., Garzon-Alvarado D.A. Effect of unit cell size, surface offset, and layer thickness on the surface roughness of gyroid TPMS lattice structures additively manufactured by mSLA. https://papers.ssrn.com/sol3/papers.cfm?abstract_id=5028165.

[115] Szatkiewicz T., Laskowska D., Bałasz B., Mitura K. (2022). The Influence of the Structure Parameters on the Mechanical Properties of Cylindrically Mapped Gyroid TPMS Fabricated by Selective Laser Melting with 316L Stainless Steel Powder. Materials.

[116] Sengsri P., Fu H., Kaewunruen S. (2022). Mechanical Properties and Energy-Absorption Capability of a 3D-Printed TPMS Sandwich Lattice Model for Meta-Functional Composite Bridge Bearing Applications. J. Compos. Sci..

[117] Hayashi K., Kishida R., Tsuchiya A., Ishikawa K. (2023). Superiority of Triply Periodic Minimal Surface Gyroid Structure to Strut-Based Grid Structure in Both Strength and Bone Regeneration. ACS Appl. Mater. Interfaces.

[118] AlWeqayyan Y., Dasinor E., Obeng B., Abbas A., Phelan P. (2023). Experimental and simulated thermal resistance of thermogalvanic cells with triply periodic minimal surface structures. Int. J. Therm. Sci..

[119] Lumba L.T., Chernyshikhin S., Mahato B., Abaimov S.G., Shishkovsky I. (2023). The assessment of permeability of biological implant structure using DLP-manufactured TPMS lattice physical models. Mater. Today Proc..

[120] Mancini E., Utzeri M., Farotti E., Lattanzi A., Sasso M. (2024). DLP printed 3D gyroid structure: Mechanical response at meso and macro scale. Mech. Mater..

[121] Chen Z.Q., Liu P.Z., Wu T.Y., Bai G.J., Xia Z.J., Han P. (2024). Parametric design and performance evaluation of TPMS porous vitrified bond diamond grinding wheel using digital light processing. Ceram. Int..

[122] Oh S.H., Ha J.W., Park K. (2022). Adaptive Conformal Cooling of Injection Molds Using Additively Manufactured TPMS Structures. Polymers.

[123] Liu B., Feng J.W., Lin Z.W., He Y., Fu J.Z. (2023). Controllable three-dimension auxetic structure design strategies based on triply periodic minimal surfaces and the application in hip implant. Virtual Phys. Prototyp..

[124] Araya-Calvo M., Järvenpää A., Rautio T., Morales-Sanchez J.E., Guillen-Girón T. (2024). Comparative fatigue performance of as-built vs etched Ti64 in TPMS-gyroid and stochastic structures fabricated via PBF-LB for biomedical applications. Rapid Prototyp. J..

[125] Singh A., Jaber A., Karathanasopoulos N. (2025). Fatigue performance of TPMS-based AlSi10Mg architected cellular materials fabricated via hybrid and powder bed fusion methods. Int. J. Fatigue.

[126] Sheng X.L., Guo A.F., Guo S., Sui S., Yang W.L., Tang R.J., Li X.J., Qu P., Wang M., Lin X. (2024). Laser powder bed fusion for the fabrication of triply periodic minimal surface lattice structures: Synergistic macroscopic and microscopic optimization. J. Manuf. Process..

[127] Shi W.T., Lin Y.X., Li J., Yang M.H., Liu B. (2024). Optimization of mechanical properties of Ti-6Al-4V triply periodic minimal surface porous structures prepared by laser beam powder bed fusion technology based on orientation control. Mat. Sci. Eng. A.

[128] Schultheiß G., Heine B., Merkel M. (2024). Characterization of flexural fatigue behaviour of additively manufactured (PBF–LB) gyroid structures. Prog. Addit. Manuf..

[129] Khrapov D., Koptyug A., Surmenev R., Surmeneva M. (2024). Expanding manufacturability of sheet-based triply periodic minimal surfaces by electron beam powder bed fusion in Wafer theme. Mater. Today Commun..

[130] Yang W.X., He W.T., Hu Z.J., Duan W., Ni X.N., Deng X., Wang A.S., Luo Y.K., Xie F.Y., Chen Z.R. (2024). Fabrication of Inconel 718 composites reinforced with TiCN via laser powder bed fusion: Integration of triply periodic minimal surface lattice structures. J. Mater. Res. Technol..

[131] Özen İ., Aslan M. (2023). Investigation of Energy Absorbing and Damage Behavior of Gyroid and Diamond Cell Based Lattice Structures Manufactured through Powder Bed Fusion Technology. Int. J. Automot. Sci. Technol..

[132] Özen İ., Çava K., İpek H., Sezer R., Aslan M. (2024). Crushing response of triply periodic minimal surface structures fabricated by investment casting and powder bed fusion method. Mater. Today Commun..

[133] Chen Z.Q., Wang X.Q., Tao Y.K., Wen S.F., Zhou Y., Shi Y.S. (2024). Volume Fraction Effect on the Mechanical and Shape Memory Properties of NiTi Gyroid Lattice Structure Fabricated by Laser Powder Bed Fusion. JOM.

[134] Liu S.Y., Feng J.L., Zhang F.L., Jiang W.B., Vasilieva T.M., Lu P., Lu S. (2024). Parametric design and performance study of continuous gradient triply periodic minimal surface bone scaffold. Int. J. Bioprinting.

[135] Anwajler B. (2022). The Thermal Properties of a Prototype Insulation with a Gyroid Structure-Optimization of the Structure of a Cellular Composite Made Using SLS Printing Technology. Materials.

[136] Abou-Ali A.M., Al-Ketan O., Lee D.W., Rowshan R., Abu Al-Rub R.K. (2020). Mechanical behavior of polymeric selective laser sintered ligament and sheet based lattices of triply periodic minimal surface architectures. Mater. Des..

[137] Thomas N., Sreedhar N., Al-Ketan O., Rowshan R., Abu Al-Rub R.K., Arafat H. (2018). 3D printed triply periodic minimal surfaces as spacers for enhanced heat and mass transfer in membrane distillation. Desalination.

[138] Xu Y., Ding W.H., Chen M.G., Guo X.P., Li P., Li M.Q. (2023). Porous iron-reinforced polylactic acid TPMS bio-scaffolds: Interlocking reinforcement and synergistic degradation. Mater. Des..

[139] Lazar P.J.L., Subramanian J., Natarajan E., Markandan K., Ramesh S. (2023). Anisotropic structure-property relations of FDM printed short glass fiber reinforced polyamide TPMS structures under quasi-static compression. J. Mater. Res. Technol..

[140] Saleh M., Anwar S., Al-Ahmari A.M., Alfaify A. (2022). Compression Performance and Failure Analysis of 3D-Printed Carbon Fiber/PLA Composite TPMS Lattice Structures. Polymers.

[141] Guo W., Yang Y., Liu C., Bu W., Guo F., Li J., Wang E., Peng Z., Mai H., You H. (2023). 3D printed TPMS structural PLA/GO scaffold: Process parameter optimization, porous structure, mechanical and biological properties. J. Mech. Behav. Biomed. Mater..

[142] Feng G.Z., Li S., Xiao L.J., Song W.D. (2023). Mechanical properties and deformation behavior of functionally graded TPMS structures under static and dynamic loading. Int. J. Impact Eng..

[143] Alemayehu D., Todoh M. (2024). Enhanced energy absorption in bioinspired combined TPMS-gyroid and walled TPMS-gyroid lattice structure manufactured via Fused Filament Fabrication (FFF). J. Manuf. Mater. Process..

[144] Peng C.X., Fox K., Qian M., Nguyen-Xuan H., Tran P. (2021). 3D printed sandwich beams with bioinspired cores: Mechanical performance and modelling. Thin Wall Struct..

[145] Li Z., Feng D., Li B., Zhao W., Xie D., Mei Y., Liu P. (2023). Ultra-Wide Range, High Sensitivity Piezoresistive Sensor Based on Triple Periodic Minimum Surface Construction. Small.

[146] Zhu X., Chen F., Cao H., Li L., He N., Han X. (2023). Design and fused deposition modeling of triply periodic minimal surface scaffolds with channels and hydrogel for breast reconstruction. Int. J. Bioprint.

[147] Du X., Ronayne S., Lee S.S., Hendry J., Hoxworth D., Bock R., Ferguson S.J. (2023). 3D-Printed PEEK/Silicon Nitride Scaffolds with a Triply Periodic Minimal Surface Structure for Spinal Fusion Implants. ACS Appl. Bio Mater..

[148] Razi S.S., Pervaiz S., Susantyoko R.A., Alyammahi M. (2024). Optimization of Environment-Friendly and Sustainable Polylactic Acid (PLA)-Constructed Triply Periodic Minimal Surface (TPMS)-Based Gyroid Structures. Polymers.

[149] Ciula A., Rubino G., Fanelli P. (2025). The Mechanical Characterization of a Gyroid-Based Metamaterial by Compression Testing. Eng. Proc..

[150] Oliveira A., Reis L., Leite M., Alves F., de Deus A.M., Sardinha M., Vaz M.F. (2022). Evaluation of cellular structures with triply periodic minimal surfaces fabricated by additive manufacturing. Manuf. Lett..

[151] Agarwal R., Malhotra S., Gupta V., Jain V. (2023). Three-dimensional printing of triply periodic minimal surface structured scaffolds for load-bearing bone defects. Polym. Eng. Sci..

[152] Fu J., Ding J.H., Qu S., Zhang L., Wang M.Y., Fu M., Song X. (2022). Improved light-weighting potential of SS316L triply periodic minimal surface shell lattices by micro laser powder bed fusion. Mater. Des..

[153] Zhang J.F., Chen X.H., Sun Y.X., Yang J.X., Chen R., Xiong Y., Hou W.S., Bai L. (2022). Design of a biomimetic graded TPMS scaffold with quantitatively adjustable pore size. Mater. Des..

[154] Zou S.J., Mu Y.R., Pan B.C., Li G.Y., Shao L., Du J.K., Jin Y. (2022). Mechanical and biological properties of enhanced porous scaffolds based on triply periodic minimal surfaces. Mater. Des..

[155] Günther F., Hirsch F., Pilz S., Wagner M., Gebert A., Kästner M., Zimmermann M. (2022). Structure-property relationships of imperfect additively manufactured lattices based on triply periodic minimal surfaces. Mater. Des..

[156] Park S.Y., Kim K.S., AlMangour B., Grzesiak D., Lee K.A. (2021). Effect of unit cell topology on the tensile loading responses of additive manufactured CoCrMo triply periodic minimal surface sheet lattices. Mater. Des..

[157] Yang N., Qian Z., Wei H.X., Zhao M. (2023). Anisotropy and deformation of triply periodic minimal surface based lattices with skew transformation. Mater. Des..

[158] Wan L., Hu D.Y., Zhang H.B., Yang Z.Y. (2024). Energy absorption of foam-filled TPMS-based tubular lattice structures subjected to quasi-static lateral crushing. Eng. Struct..

[159] Ge J., Huang Q., Wang Y., Zhang C., Liu Q., Lu Z., Yin S. (2023). Microstructural optimization and mechanical enhancement of SLM Ti6Al4V TPMS scaffolds through vacuum annealing treatment. J. Alloys Compd..

[160] Maszybrocka J., Gapinski B., Dworak M., Skrabalak G., Stwora A. (2019). The manufacturability and compression properties of the Schwarz Diamond type Ti6Al4V cellular lattice fabricated by selective laser melting. Int. J. Adv. Manuf. Tech..

[161] Maszybrocka J., Gapinski B., Dworak M., Skrabalak G., Stwora A. (2019). Modelling, manufacturability and compression properties of the CpTi grade 2 cellular lattice with radial gradient TPMS architecture. Bull. Pol. Acad. Sci. Tech. Sci..

[162] Yan G.H., Sun M.R., Zhang Z.D., Liang Y.Q., Jiang N., Pang X.D., Song Y.C., Liu Y., Zhao J.F. (2023). Experimental study on flow and heat transfer performance of triply periodic minimal surface structures and their hybrid form as disturbance structure. Int. Commun. Heat Mass.

[163] Liang D., Shi C., Li W., Chen W., Chyu M.K. (2023). Design, flow characteristics and performance evaluation of bioinspired heat exchangers based on triply periodic minimal surfaces. Int. J. Heat Mass Transf..

[164] Zhu J., Zou S., Mu Y., Wang J., Jin Y. (2022). Additively Manufactured Scaffolds with Optimized Thickness Based on Triply Periodic Minimal Surface. Materials.

[165] Yang L., Yan C.Z., Cao W.C., Liu Z.F., Song B., Wen S.F., Zhang C., Shi Y.S., Yang S.F. (2019). Compression-compression fatigue behaviour of gyroid-type triply periodic minimal surface porous structures fabricated by selective laser melting. Acta Mater..

[166] Qiu N., Zhang J.Z., Li C.Y., Shen Y.J., Fang J.G. (2023). Mechanical properties of three-dimensional functionally graded triply periodic minimum surface structures. Int. J. Mech. Sci..

[167] Ye J., He W., Wei T., Sun C., Zeng S. (2023). Mechanical Properties Directionality and Permeability of Fused Triply Periodic Minimal Surface Porous Scaffolds Fabricated by Selective Laser Melting. ACS Biomater. Sci. Eng..

[168] Pires T., Santos J., Ruben R.B., Gouveia B.P., Castro A.P.G., Fernandes P.R. (2021). Numerical-experimental analysis of the permeability-porosity relationship in triply periodic minimal surfaces scaffolds. J. Biomech..

[169] Xie Y.M., Mao Y.W., Heng Y.H., Tao J.Q., Xiang L., Qin X.Y., Wei Q.S. (2025). Mechanical responses of triply periodic minimal surface gyroid lattice structures fabricated by binder jetting additive manufacturing. J. Mater. Res. Technol..

[170] Wu H.D., Jiang C., Tang C., Wang L., Huang S.W., Ge S., Li B., Deng X., Wu S.H. (2024). Binder jetting printed in situ mullite strengthened alumina ceramics with excellent mechanical and thermal properties through multi-phase infiltration. Virtual Phys. Prototyp..

[171] Shevchenko V., Balabanov S., Sychov M., Karimova L. (2023). Prediction of Cellular Structure Mechanical Properties with the Geometry of Triply Periodic Minimal Surfaces (TPMS). ACS Omega.

[172] Alam M.R., Khondoker M.A.H. (2024). Characterization of Post-Sintering Shrinkage of Ceramic-Based Lattice Structures Printed Using LCD Resin Printer. Eng. Proc..

[173] Li H., Liang L., Zeng W.X., Deng Y.C., Ge N.P., Shan W.B. (2023). 3D printing polyurethane acrylate(PUA) based elastomer and its mechanical behavior. Mater. Res. Express.

[174] Kancs A. (2025). Manufacturability and Performance Study of Triply Periodic Minimal Surface Air-to-Air Heat Exchanger. Latv. J. Phys. Tech. Sci..

[175] Peng K., Yu T., Wu P., Chen M. (2024). Piezoresistive Porous Composites with Triply Periodic Minimal Surface Structures Prepared by Self-Resistance Electric Heating and 3D Printing. Sensors.

[176] Alteneiji M., Ali M.I.H., Khan K.A., Al-Rub R.K.A. (2022). Heat transfer effectiveness characteristics maps for additively manufactured TPMS compact heat exchangers. Energy Storage Sav..

[177] Asbai-Ghoudan R., Ruiz de Galarreta S., Rodriguez-Florez N. (2021). Analytical model for the prediction of permeability of triply periodic minimal surfaces. J. Mech. Behav. Biomed. Mater..

[178] Attarzadeh R., Rovira M., Duwig C. (2021). Design analysis of the “Schwartz D” based heat exchanger: A numerical study. Int. J. Heat Mass Transf..

[179] Baghous N., Barsoum I., Abu Al-Rub R.K. (2023). Generalized yield surface for sheet-based triply periodic minimal surface lattices. Int. J. Mech. Sci..

[180] Qureshi Z.A., Al-Omari S.A.B., Elnajjar E., Al-Ketan O., Al-Rub R.A. (2022). On the effect of porosity and functional grading of 3D printable triply periodic minimal surface (TPMS) based architected lattices embedded with a phase change material. Int. J. Heat Mass Transf..

[181] Bakhtiari H., Nouri A., Tolouei-Rad M. (2024). Fatigue Performance of 3D-Printed Poly-Lactic-Acid Bone Scaffolds with Triply Periodic Minimal Surface and Voronoi Pore Structures. Polymers.

[182] Diez-Escudero A., Harlin H., Isaksson P., Persson C. (2020). Porous polylactic acid scaffolds for bone regeneration: A study of additively manufactured triply periodic minimal surfaces and their osteogenic potential. J. Tissue Eng..

[183] Maevskaia E., Guerrero J., Ghayor C., Bhattacharya I., Weber F.E. (2023). Triply periodic minimal surface-based scaffolds for bone tissue engineering: A mechanical, in vitro and in vivo study. Tissue Eng. Part A.

[184] Melchels F.P., Bertoldi K., Gabbrielli R., Velders A.H., Feijen J., Grijpma D.W. (2010). Mathematically defined tissue engineering scaffold architectures prepared by stereolithography. Biomaterials.

[185] Cobian L., Maire E., Adrien J., Freitas U., Fernández-Blázquez J., Monclús M., Segurado J. (2024). Effect of sample dimensions on the stiffness of PA12 Lattice materials fabricated using Powder Bed Fusion. Addit. Manuf..

[186] Colucci G., Fontana L., Barberi J., Vitale Brovarone C., Messori M. (2024). Chess-like Pieces Realized by Selective Laser Sintering of PA12 Powder: 3D Printing and Micro-Tomographic Assessment. Polymers.

[187] Naghavi S.A., Tamaddon M., Marghoub A., Wang K., Babamiri B.B., Hazeli K., Xu W., Lu X., Sun C., Wang L. (2022). Mechanical characterisation and numerical modelling of TPMS-based gyroid and diamond Ti6Al4V scaffolds for bone implants: An integrated approach for translational consideration. Bioengineering.

[188] Li H., Liang X., Li Y., Lin F. (2022). Performance of high-layer-thickness Ti6Al4V fabricated by electron beam powder bed fusion under different accelerating voltage values. Materials.

[189] Maculotti G., Piscopo G., Marchiandi G., Atzeni E., Salmi A., Iuliano L. (2022). Build orientation effect on Ti6Al4V thin-wall topography by electron beam powder bed fusion. Procedia CIRP.

[190] Megahed S., Aniko V., Schleifenbaum J.H. (2022). Electron beam-melting and laser powder bed fusion of Ti6Al4V: Transferability of process parameters. Metals.

[191] Meisel N.A., Williams C.B. Design for additive manufacturing: An investigation of key manufacturing considerations in multi-material polyjet 3d printing. Proceedings of the 2014 International Solid Freeform Fabrication Symposium.

[192] Borujeni S.S., Shad A., Venkata K.A., Günther N., Ploshikhin V. (2022). Numerical simulation of shrinkage and deformation during sintering in metal binder jetting with experimental validation. Mater. Des..

[193] Li M., Du W., Elwany A., Pei Z., Ma C. (2020). Metal binder jetting additive manufacturing: A literature review. J. Manuf. Sci. Eng..

[194] Ziaee M., Crane N.B. (2019). Binder jetting: A review of process, materials, and methods. Addit. Manuf..

[195] Paudel B.J., Conover D., Lee J.-K., To A.C. (2021). A computational framework for modeling distortion during sintering of binder jet printed parts. J. Micromech. Mol. Phys..

[196] Özel T., Shokri H., Loizeau R. (2023). A review on wire-fed directed energy deposition based metal additive manufacturing. J. Manuf. Mater. Process..

[197] Singh A., Kapil S., Das M. (2020). A comprehensive review of the methods and mechanisms for powder feedstock handling in directed energy deposition. Addit. Manuf..

[198] Jacobs P.F. (1992). Fundamentals of Stereolithography.

[199] Yap C.Y., Chua C.K., Dong Z.L., Liu Z.H., Zhang D.Q., Loh L.E., Sing S.L. (2015). Review of selective laser melting: Materials and applications. Appl. Phys. Rev..

[200] Herzog D., Seyda V., Wycisk E., Emmelmann C. (2016). Additive manufacturing of metals. Acta Mater..

[201] Chartier T., Chaput C., Doreau F., Loiseau M. (2002). Stereolithography of structural complex ceramic parts. J. Mater. Sci..

[202] Turner B.N., Strong R., Gold S.A. (2014). A review of melt extrusion additive manufacturing processes: I. Process design and modeling. Rapid Prototyp. J..

[203] Liu F., Mao Z.F., Zhang P., Zhang D.Z., Jiang J.J., Ma Z.B. (2018). Functionally graded porous scaffolds in multiple patterns: New design method, physical and mechanical properties. Mater. Des..

[204] Yan C.Z., Hao L., Hussein A., Raymont D. (2012). Evaluations of cellular lattice structures manufactured using selective laser melting. Int. J. Mach. Tools Manuf..

[205] Maskery I., Aremu A.O., Parry L., Wildman R.D., Tuck C.J., Ashcroft I.A. (2018). Effective design and simulation of surface-based lattice structures featuring volume fraction and cell type grading. Mater. Des..

[206] Chen Z.Y., Xie Y.M., Wu X., Wang Z., Li Q., Zhou S.W. (2019). On hybrid cellular materials based on triply periodic minimal surfaces with extreme mechanical properties. Mater. Des..

[207] Yoo D.J., Kim K.H. (2015). An advanced multi-morphology porous scaffold design method using volumetric distance field and beta growth function. Int. J. Precis. Eng. Manuf..

[208] Novak N., Borovinsek M., Al-Ketan O., Ren Z.R., Vesenjak M. (2022). Impact and blast resistance of uniform and graded sandwich panels with TPMS cellular structures. Compos. Struct..

[209] Zhong M., Zhou W., Xi H., Liang Y., Wu Z. (2021). Double-Level Energy Absorption of 3D Printed TPMS Cellular Structures via Wall Thickness Gradient Design. Materials.

[210] Jones A., Leary M., Bateman S., Easton M. (2023). Investigating mechanical properties of additively manufactured multimaterial gyroids: The effect of proportion, scale and shape. Addit. Manuf..

[211] Altamimi S., Lee D.-W., Barsoum I., Rowshan R., Jasiuk I.M., Abu Al-Rub R.K. (2025). Stiffness, strength, anisotropy, and buckling of lattices derived from TPMS and Platonic and Archimedean solids. Mech. Adv. Mater. Struct..

[212] Shaikh A., Griffis J., Stebbins R., Shahed K.S., Saxena A., Ross A., Manogharan G. (2024). Towards Gradient Design of TPMS lattices and Laser Powder Bed Fusion Processing- Role of Laser Strategies and Lattice Thickness. Manuf. Lett..

[213] Xu Z., Sarasini F., Medori E., Berto F., Razavi N. (2024). Scale-dependent mechanical performance variations in polylactic acid lattice structures fabricated via additive manufacturing. Fatigue Fract. Eng. Mater. Struct..

[214] Yeo J., Cheung S., Gu X.W., Ryu S. (2025). Hybrid TPMS-based architectured materials (HTAM) for enhanced specific stiffness using data-driven design. Mater. Des..

[215] Wei Y.P., Li H.Q., Han J.J., Ma Y.C., Zhou H.R., Cheng J.C., Shi J., Miao Z.Q., Yu B., Lin F. (2024). Mechanical and damping performances of TPMS lattice metamaterials fabricated by laser powder bed fusion. China Foundry.

[216] Hu D., Wang J.Y., Liao Z.R., Fu M. (2025). Localized strengthening of triply periodic minimal surface lattice structures via tuning the internal material distribution at the grain level. Addit. Manuf..

[217] Dyer K., Amjadi M., Shao S., Shamsaei N., Molaei R. (2025). Understanding Fatigue of Additively Manufactured TPMS Metallic Metamaterials: Experiments and Modeling. Addit. Manuf..

[218] Wang A., Ni X., He D., Hu Z., Yang W., Deng X., Wu S. (2025). Compression Behavior and Energy Absorption of TPMS Structures Prepared by Laser Powder Bed Fusion. Metall. Mater. Trans. B.

[219] Mirzavand K., Zamzamian S.A.H., Adl M., Seyedraoufi Z.-S., Maghsoudipour A. (2025). Investigation on mechanical characteristics of modified TPMS primitive PLA-CF mechanical metamaterials. Sci. Rep..

[220] Li M., Xu Y., Wu C., Li Q., Wu C., Fang J. (2025). Mechanical performance of FDM-printed PLA TPMS lattices under hydrothermal aging. Thin-Walled Struct..

[221] Lu C.X., Ding J., Jiang X., Wen P., Zhang C., Shen Q., Chen F. (2024). Enhancing mechanical properties and damage tolerance of additive manufactured ceramic TPMS lattices by hybrid design. J. Am. Ceram. Soc..

[222] Hu Z., Ni X., Yang W., Duan W., Deng X., Liu J., Wu S. (2025). Optimizing microstructure and mechanical performance in LPBF-processed TiCN-AlZnMgCu composites: From composition to TPMS reinforcement. J. Alloys Compd..

[223] Fan H.L., Yassin A., Tamrin K.F., Hamdan S., Wang C. (2024). Optimized design and mechanical properties of TPMS porous structures based on selective laser sintering. Matéria.

[224] Huang X.Y., Tang H.Y., Wang L. (2024). Effect of residual stress on mechanical properties of Triply periodic minimal surface lattice structures in Additive manufacturing. Comp. Mater. Sci..

[225] Chitrakar R., Hossain M.S., Nilufar S. (2023). The Effect of Microballoon Volume Fraction on the Elastic and Viscoelastic Properties of Hollow Microballoon-Filled Epoxy Composites. Materials.

[226] Huffman B., Singh A., Koohbor B., Youssef G. (2024). Vat photopolymerization 3D printing of glass microballoon-reinforced TPMS meta-structures. Compos. B Eng..

[227] Kim D.Y., Kim H.S., Kamath S.S., Hou X., Choi J.W., Park S.H. (2024). TPMS-based auxetic structure for high-performance airless tires with variable stiffness depending on deformation. Sci. Rep..

[228] Chawla K., Kiran R. (2022). Numerical predictions for the effect of negative Poisson’s ratio on thermoelastic properties of triply periodic minimal surface-based composites. Results Mater..

[229] Lin Z.H., Pan J.H., Li H.Y. (2022). Mechanical Strength of Triply Periodic Minimal Surface Lattices Subjected to Three-Point Bending. Polymers.

[230] Sixt J., Davoodi E., Salehian A., Toyserkani E. (2023). Characterization and optimization of 3D-printed, flexible vibration strain sensors with triply periodic minimal surfaces. Addit. Manuf..

[231] Peng C.X., Marzocca P., Tran P. (2023). Triply periodic minimal surfaces based honeycomb structures with tuneable mechanical responses. Virtual Phys. Prototyp..

[232] Préve D., Lenarda P., Maskery I., Paggi M. (2023). A comprehensive characterization of fracture in unit cell open foams generated from triply periodic minimal surfaces. Eng. Fract. Mech..

[233] Gawronska E., Dyja R. (2021). A Numerical Study of Geometry’s Impact on the Thermal and Mechanical Properties of Periodic Surface Structures. Materials.

[234] Wu H., Li D., Yang B., Yu S. (2025). Improved bending strength and energy absorption in SLM Cu-Cr-Zr lattice-beams using shaped graded TPMS lattice structures. Mater. Today Commun..

[235] Dalaq A.S., Abueidda D.W., Abu Al-Rub R.K. (2016). Mechanical properties of 3D printed interpenetrating phase composites with novel architectured 3D solid-sheet reinforcements. Compos. Part A Appl. Sci. Manuf..

[236] Zhang M.K., Li J.W., Liu C., Deng M.J., Liao X., Wang D. (2023). Study on the Anisotropy of Triply Periodic Minimal Surface Porous Structures. Coatings.

[237] Khaleghi S., Dehnavi F.N., Baghani M., Safdari M., Wang K., Baniassadi M. (2021). On the directional elastic modulus of the TPMS structures and a novel hybridization method to control anisotropy. Mater. Des..

[238] Van Viet N., Abu Al-Rub R., Zaki W. (2022). Mechanical behavior of shape-memory alloy triply periodic minimal surface foam based on schwarz primitive. J. Eng. Mech..

[239] Viet N.V., Zaki W. (2023). Artificial neural network model of the mechanical behaviour of shape memory alloy Schwartz primitive lattice architectures. Mech. Mater..

[240] Sun L.Q., Chen K.Y., Geng P., Zhou Y., Wen S.F., Shi Y.S. (2023). Mechanical and shape memory properties of NiTi triply periodic minimal surface structures fabricated by laser powder bed fusion. J. Manuf. Process..

[241] Reynolds B.W. (2020). Simulation of Flow and Heat Transfer in 3D Printable Triply Periodic Minimal Surface Heat Exchangers. Ph.D. Thesis.

[242] Padrão D., Hancock D., Paterson J., Schoofs F., Tuck C., Maskery I. (2024). New structure-performance relationships for surface-based lattice heat sinks. Appl. Therm. Eng..

[243] Liu C., Zhang M., Bi G., Chen J., Bai Y., Wang D., Deng M. (2025). Research on comprehensive heat dissipation characteristics of AlSi7Mg TPMS heat sinks manufactured by laser powder bed fusion. Appl. Therm. Eng..

[244] Guillermo O.R.L., Arturo G.O., James P.B., Saul P. (2025). Computational analysis and engineering modeling for the heat transfer and fluid flow through the gyroid TPMS structure. Appl. Therm. Eng..

[245] Wang H.-C., Zheng S.-F., Liu G., Yan K.-X., Yang Y.-R., Deng H.-W., Du Q., Wang X.-D. (2025). Influences of the flow rate and fluid volume in air-kerosene cross-flow heat exchangers using Gyroid-typed triply periodic minimal surfaces. Appl. Therm. Eng..

[246] Kruzel M., Dutkowski K., Bohdal T. (2025). Experimental Studies of Fluid Flow Resistance in a Heat Exchanger Based on the Triply Periodic Minimal Surface. Energies.

[247] Lai W.H., Samad A. (2025). Development and flow optimization of “Gyroid” based additive manufacturing heat exchanger: Both computational and experimental analyses. Int. J. Therm. Sci..

[248] Reynolds B., Lecarpentier F., Holland D. (2025). Heat transfer and topological characterisation of TPMS structures using 3D printed materials. Int. J. Heat Mass Transf..

[249] Men Z., Chen W.J., Li Q.H., Liu S.T. (2025). Topology optimization of the IWP triply periodic minimal surfaces (TPMS) heat sink based on porous media effective model. Int. J. Heat Mass Transf..

[250] Qin K.W., Zhuang N.L., Shao C., Zhao H.B., Tang X.B. (2025). Gyroid-type TPMS structure optimization based on mathematical function control and its convective heat transfer performance study. Int. Commun. Heat Mass.

[251] Renon C., Jeanningros X. (2025). A numerical investigation of heat transfer and pressure drop correlations in Gyroid and Diamond TPMS-based heat exchanger channels. Int. J. Heat Mass Transf..

[252] Yan G.H., Zhang Z.D., Li S., Zhang X.K., Zhang X.Y., Duan J.T., Sun M.R., Liu Y., Song Y.C. (2025). Investigative research on the heat exchangers with triply periodic minimal surface structures under two hybrid methods. Int. J. Heat Mass Transf..

[253] Yan K.X., Deng H.W., Xiao Y.W., Wang J.W., Luo Y.Y. (2024). Thermo-hydraulic performance evaluation through experiment and simulation of additive manufactured Gyroid-structured heat exchanger. Appl. Therm. Eng..

[254] Li W.G., Li W.H., Yu Z.B. (2022). Heat transfer enhancement of water-cooled triply periodic minimal surface heat exchangers. Appl. Therm. Eng..

[255] Wadsö I., Holmqvist S. (2020). Additively Manufactured Heat Exchangers—Development and Testing. Master’s Thesis.

[256] Gao S.M., Qu S., Ding J.H., Liu H., Song X. (2023). Influence of cell size and its gradient on thermo-hydraulic characteristics of triply periodic minimal surface heat exchangers. Appl. Therm. Eng..

[257] Yan K.X., Wang J.W., Li L.A., Deng H.W. (2023). Numerical investigation into thermo-hydraulic characteristics and mixing performance of triply periodic minimal surface-structured heat exchangers. Appl. Therm. Eng..

[258] Oh S.-H., Kim J.E., Jang C.H., Kim J., Park C.Y., Park K. (2025). Multifunctional gradations of TPMS architected heat exchanger for enhancements in flow and heat exchange performances. Sci. Rep..

[259] Gado M.G., Ookawara S., Hassan H. (2023). Utilization of triply periodic minimal surfaces for performance enhancement of adsorption cooling systems: Computational fluid dynamics analysis. Energy Convers. Manag..

[260] Fan Z., Fu Y., Gao R., Liu S. (2023). Investigation on heat transfer enhancement of phase change material for battery thermal energy storage system based on composite triply periodic minimal surface. J. Energy Storage.

[261] Ansari D., Duwig C. (2024). A gyroid TPMS heat sink for electronic cooling. Energy Convers. Manag..

[262] Chen J., Liu X., Li Y., Feng X., Chen J., Zhu H., Tao W.-Q. (2025). Numerical and experimental investigation of TPMS-structured cold plates for electronic device cooling. Appl. Energy.

[263] Barakat A., Sun B.B. (2024). Enhanced convective heat transfer in new triply periodic minimal surface structures: Numerical and experimental investigation. Int. J. Heat Mass Transf..

[264] Brambati G., Guilizzoni M., Foletti S. (2024). Convective heat transfer correlations for Triply Periodic Minimal Surfaces based heat exchangers. Appl. Therm. Eng..

[265] Wang J.X., Qian C.Y., Yu B.B., Zhang F.R., Ma R.X., Shi J.Y., Chen J.P. (2024). Design and optimization of additive manufactured Fischer-Koch-structured heat exchanger for enhanced heat transfer efficiency. Int. Commun. Heat Mass.

[266] Yan K.X., Deng H.W., Xiao Y.W., Wang J.W., Luo Y.Y., Yan J.Q. (2024). Influence of polishing process on surface morphology and thermo-hydraulic performance of additively manufactured Gyroid-structured heat exchanger. Appl. Therm. Eng..

[267] Zhang Y., Zhang X.K., Li H.Y., Li S., Zhang Z.D., Sun M.R., Song Y.C. (2025). Improving the efficiency of solar thermal storage systems using TPMS: A pore-scale simulation. Sol. Energy Mater. Sol. Cells.

[268] Huang W.S., Ning H.Y., Li N., Tang G.H., Ma Y., Li Z., Nan X.Y., Li X.H. (2024). Thermal-hydraulic performance of TPMS-based regenerators in combined cycle aero-engine. Appl. Therm. Eng..

[269] Pelanconi M., Bottacin S., Bianchi G., Viganò D., Papageorgiou V., Strakov H., Ortona A. (2025). TPMS SiC structures produced by chemical vapour infiltration of SiC preforms shaped by powder bed fusion and binder jetting: A preliminary study on the early stages of the CVI process. J. Eur. Ceram. Soc..

[270] Qureshi Z.A., Elnajjar E., Al-Ketan O., Abu Al-Rub R., Al-Omari S.B. (2021). Heat transfer performance of a finned metal foam-phase change material (FMF-PCM) system incorporating triply periodic minimal surfaces (TPMS). Int. J. Heat Mass Transf..

[271] Femmer T., Kuehne A.J.C., Wessling M. (2015). Estimation of the structure dependent performance of 3-D rapid prototyped membranes. Chem. Eng. J..

[272] Dharmalingam L.K., Aute V., Ling J. Review of triply periodic minimal surface (TPMS) based heat exchanger designs. Proceedings of the International Refrigeration and Air Conditioning Conference.

[273] Zhang T., Liu F., Zhang K.F., Zhao M., Zhou H.L., Zhang D.Z. (2023). Numerical study on the anisotropy in thermo-fluid behavior of triply periodic minimal surfaces (TPMS). Int. J. Heat Mass Transf..

[274] Fan Z.H., Gao R.J., Liu S.T. (2022). Thermal conductivity enhancement and thermal saturation elimination designs of battery thermal management system for phase change materials based on triply periodic minimal surface. Energy.

[275] Penubarthi G.V., Suresh Babu K.B., Sundararaj S., Kang S.W. (2025). Investigating Experimental and Computational Fluid Dynamics of 3D-Printed TPMS and Lattice Porous Structures. Micromachines.

[276] Rajagopalan S., Robb R.A. (2006). Schwarz meets Schwann: Design and fabrication of biomorphic and durataxic tissue engineering scaffolds. Med. Image Anal..

[277] Kapfer S.C., Hyde S.T., Mecke K., Arns C.H., Schroder-Turk G.E. (2011). Minimal surface scaffold designs for tissue engineering. Biomaterials.

[278] Alemayehu D.B., Todoh M., Huang S.J. (2025). Hybrid Biomechanical Design of Dental Implants: Integrating Solid and Gyroid Triply Periodic Minimal Surface Lattice Architectures for Optimized Stress Distribution. J. Funct. Biomater..

[279] Baumer V., Isaacson N., Kanakamedala S., McGee D., Kaze I., Prawel D. (2024). Comparing ceramic Fischer-Koch-S and gyroid TPMS scaffolds for potential in bone tissue engineering. Front. Bioeng. Biotechnol..

[280] Cheloni J.P.M., Zluhan B., Silveira M.E., Fonseca E.B., Valim D.B., Lopes E.S.N. (2025). Mechanical behavior and failure mode of body-centered cubic, gyroid, diamond, and Voronoi functionally graded additively manufactured biomedical lattice structures. J. Mech. Behav. Biomed. Mater..

[281] D’Andrea L., Gabrieli R., Milano L., Magagnin L., De Cet A., Alidoost D., Schwentenwein M., Verné E., Baino F., Vena P. (2025). Elastic and failure characterization of hydroxyapatite TPMS scaffolds using a combined approach of ultrasound, compression tests and micro-CT based numerical models. Acta Mater..

[282] Firoz A.b., Rybakov V., Fetisova A.A., Shlapakova L.E., Pariy I.O., Toropkov N., Lozhkomoev A.S., Mukhortova Y.R., Sharonova A.A., Wagner D.V. (2025). 3D-printed biodegradable composite poly (lactic acid)-based scaffolds with a shape memory effect for bone tissue engineering. Adv. Compos. Hybrid Mater..

[283] Gunashekar G., Reddy N.D.D., Penumakala P.K., Narala S.K.R. (2025). Compressive response of additively manufactured Ti-6Al-4V Triply Periodic Minimal Surface structures with different unit cell designs for biomedical implant applications. Mater. Today Commun..

[284] Wang J., Huang Z., Han Z., Luan J., Li Z., Guo X., Yang D., Cui Y., Han J., Xu D. (2025). TPMS-Gyroid Scaffold-Mediated Up-Regulation of ITGB1 for Enhanced Cell Adhesion and Immune-Modulatory Osteogenesis. Adv. Healthc. Mater..

[285] Koushik T.M., Miller C.M., Antunes E. (2025). Graded Hydroxyapatite Triply Periodic Minimal Surface Structures for Bone Tissue Engineering Applications. Adv. Healthc. Mater..

[286] Chen W. (2021). Laser Powder Bed Fusion of Shape Memory Alloy NiTi for Biomedical Applications.

[287] Melchels F.P., Barradas A.M., van Blitterswijk C.A., de Boer J., Feijen J., Grijpma D.W. (2010). Effects of the architecture of tissue engineering scaffolds on cell seeding and culturing. Acta Biomater..

[288] Yoo D.J. (2011). Computer-aided Porous Scaffold Design for Tissue Engineering Using Triply Periodic Minimal Surfaces. Int. J. Precis. Eng. Manuf..

[289] Tikhonov A., Evdokimov P., Klimashina E., Tikhonova S., Karpushkin E., Scherbackov I., Dubrov V., Putlayev V. (2020). Stereolithographic fabrication of three-dimensional permeable scaffolds from CaP/PEGDA hydrogel biocomposites for use as bone grafts. J. Mech. Behav. Biomed. Mater..

[290] Tsai Y.-Y., Chang S.-W. (2023). Pullout strength of triply periodic minimal surface-structured bone implants. Int. J. Mech. Sci..

[291] Kainz M., Perak S., Stubauer G., Kopp S., Kauscheder S., Hemetzberger J., Martinez Cendrero A., Diaz Lantada A., Tupe D., Major Z. (2024). Additive and Lithographic Manufacturing of Biomedical Scaffold Structures Using a Versatile Thiol-Ene Photocurable Resin. Polymers.

[292] Shen M., Li Y., Lu F., Gou Y., Zhong C., He S., Zhao C., Yang G., Zhang L., Yang X. (2023). Bioceramic scaffolds with triply periodic minimal surface architectures guide early-stage bone regeneration. Bioact. Mater..

[293] Li L., Wang P., Liang H., Jin J., Zhang Y., Shi J., Zhang Y., He S., Mao H., Xue B. (2023). Design of a Haversian system-like gradient porous scaffold based on triply periodic minimal surfaces for promoting bone regeneration. J. Adv. Res..

[294] Belda R., Megias R., Marco M., Vercher-Martinez A., Giner E. (2023). Numerical analysis of the influence of triply periodic minimal surface structures morphometry on the mechanical response. Comput. Methods Programs Biomed..

[295] Blanquer S.B.G., Werner M., Hannula M., Sharifi S., Lajoinie G.P.R., Eglin D., Hyttinen J., Poot A.A., Grijpma D.W. (2017). Surface curvature in triply-periodic minimal surface architectures as a distinct design parameter in preparing advanced tissue engineering scaffolds. Biofabrication.

[296] Kumar P.V., Birru A.K., Muthu N. (2025). Multi-response optimization of FDM parameters for high-performance PLA/MgTiO3 TPMS gyroid scaffolds: A Taguchi-based VIKOR–TOPSIS approach for biomedical applications. Progress Addit. Manuf..

[297] Kumar P.V., Pal S., Birru A.K., Jaganathan B.G., Muthu N. (2025). 3D-printed TPMS-structured hybrid PLA/MgTiO3 scaffolds: Synergizing bioactivity and antibacterial performance for bone regeneration. Biomater. Adv..

[298] Reshadinezhad M., Badrossamay M., Foroozmehr E., Ghaei A. (2025). Design and mechanical characterization of TPMS cellular structures additively manufactured by the selective laser melting process for use in intervertebral lumbar cages. Progress Addit. Manuf..

[299] Karuna C., Poltue T., Khrueaduangkham S., Promoppatum P. (2022). Mechanical and fluid characteristics of triply periodic minimal surface bone scaffolds under various functionally graded strategies. J. Comput. Des. Eng..

[300] Li J., Chen M., Fan X., Zhou H. (2016). Recent advances in bioprinting techniques: Approaches, applications and future prospects. J. Transl. Med..

[301] Wu C., Wan B., Entezari A., Fang J., Xu Y., Li Q. (2024). Machine learning-based design for additive manufacturing in biomedical engineering. Int. J. Mech. Sci..

[302] Ziaie B., Velay X., Saleem W. (2024). Exploring the optimal mechanical properties of triply periodic minimal surface structures for biomedical applications: A Numerical analysis. J. Mech. Behav. Biomed. Mater..

[303] Werner J.G., Rodríguez-Calero G.G., Abruña H.D., Wiesner U. (2018). Block copolymer derived 3-D interpenetrating multifunctional gyroidal nanohybrids for electrical energy storage. Energy Environ. Sci..

[304] Yang H., Wang Z., Bao R., Zhang B., Zhu X., Wang H. (2025). A novel hybrid battery thermal management system using TPMS structure and delayed cooling scheme. Appl. Therm. Eng..

[305] Zhang T., Deng X., Zhao M., Zhou H., Zhang D.Z. (2022). Experimental study on the thermal storage performance of phase change materials embedded with additively manufactured triply periodic minimal surface architected lattices. Int. J. Heat Mass Transf..

[306] Qureshi Z.A., Al-Omari S.A.B., Elnajjar E., Al-Ketan O., Al-Rub R.A. (2022). Nature-inspired triply periodic minimal surface-based structures in sheet and solid configurations for performance enhancement of a low-thermal-conductivity phase-change material for latent-heat thermal-energy-storage applications. Int. J. Therm. Sci..

[307] Lei H.Y., Li J.R., Wang Q.H., Xu Z.J., Zhou W., Yu C.L., Zheng T.Q. (2019). Feasibility of preparing additive manufactured porous stainless steel felts with mathematical micro pore structure as novel catalyst support for hydrogen production via methanol steam reforming. Int. J. Hydrogen Energy.

[308] Sreedhar N., Thomas N., Al-Ketan O., Rowshan R., Hernandez H.H., Abu Al-Rub R.K., Arafat H.A. (2018). Mass transfer analysis of ultrafiltration using spacers based on triply periodic minimal surfaces: Effects of spacer design, directionality and voidage. J. Membr. Sci..

[309] Hawken M.B., Reid S., Clarke D.A., Watson M., Fee C.J., Holland D.J. (2023). Characterization of pressure drop through Schwarz-Diamond triply periodic minimal surface porous media. Chem. Eng. Sci..

[310] Terrett R.N.L., Frankcombe T.J. (2023). Reactive Bimetallic Nanostructures Based on Triply Periodic Minimal Surfaces: A Molecular Dynamics Study toward the Limits of Performance. ACS Appl. Mater. Interfaces.

[311] Bertero A., Coppola B., Schmitt J., Gimello O., Trens P., Palmero P., Tulliani J.-M. (2025). 3D printed mullite monoliths with triply periodic minimal surface (TPMS) architectures functionalized with HKUST-1 for CO2 capture. Microporous Mesoporous Mater..

[312] Yu K.H., Teng T., Nah S.H., Chai H., Zhi Y., Wang K.Y., Chi Y., Psarras P., Akbarzadeh M., Yang S. (2025). 3D Concrete Printing of Triply Periodic Minimum Surfaces for Enhanced Carbon Capture and Storage. Adv. Funct. Mater..

[313] Xu D., Zhao L., Lin M. (2024). Optimization of porous structures via machine learning for solar thermochemical fuel production. Prog. Nat. Sci. Mater. Int..

[314] Gado M.G., Megahed T.F., Ookawarad S., Nada S., El-Sharkawy I.I. (2021). Performance and economic analysis of solar-powered adsorption-based hybrid cooling systems. Energy Convers. Manag..

[315] Kurup M., Pitchaimani J. (2023). Aeroelastic flutter of triply periodic minimal surface (TPMS) beams. Compos. Part C Open Access.

[316] Abueidda D.W., Jasiuk I., Sobh N.A. (2018). Acoustic band gaps and elastic stiffness of PMMA cellular solids based on triply periodic minimal surfaces. Mater. Des..

[317] Dolan J.A., Dehmel R., Demetriadou A., Gu Y., Wiesner U., Wilkinson T.D., Gunkel I., Hess O., Baumberg J.J., Steiner U. (2019). Metasurfaces Atop Metamaterials: Surface Morphology Induces Linear Dichroism in Gyroid Optical Metamaterials. Adv. Mater..

[318] Tran P., Peng C. (2021). Triply periodic minimal surfaces sandwich structures subjected to shock impact. J. Sandw. Struct. Mater..

[319] Feng H.B., Xu C.H., Xiao L.H., Li L. (2025). Multiscale hierarchical composite with extremely specific damping performance via bottom-up synergistic enhancement strategy. Virtual Phys. Prototyp..

[320] Weber D., Sundarram S.S. (2023). 3D-printed and foamed triply periodic minimal surface lattice structures for energy absorption applications. Polym. Eng. Sci..

[321] Ellebracht N.C., Roy P., Moore T., Gongora A.E., Oyarzun D.I., Stolaroff J.K., Nguyen D.T. (2023). 3D printed triply periodic minimal surfaces as advanced structured packings for solvent-based CO 2 capture. Energy Environ. Sci..

[322] Abueidda D.W., Abu Al-Rub R.K., Dalaq A.S., Lee D.W., Khan K.A., Jasiuk I. (2016). Effective conductivities and elastic moduli of novel foams with triply periodic minimal surfaces. Mech. Mater..

[323] Kolibaba T.J., Iverson E.T., Legendre H., Higgins C.I., Buck Z.N., Weeks T.S., Grunlan J.C., Killgore J.P. (2023). Synergistic Fire Resistance of Nanobrick Wall Coated 3D Printed Photopolymer Lattices. ACS Appl. Mater. Interfaces.

[324] Li X., Ouyang H., Sun S.J., Wang J.Z., Fei G.X., Xia H.S. (2023). Selective Laser Sintering for Electrically Conductive Poly(dimethylsiloxane) Composites with Self-Healing Lattice Structures. ACS Appl. Polym. Mater..

[325] Lesmana L.A., Aziz M. (2023). Adoption of triply periodic minimal surface structure for effective metal hydride-based hydrogen storage. Energy.

[326] Lesmana L.A., Lu C., Chen F., Aziz M. (2023). Triply periodic minimal surface gyroid structure as effective metal hydride hydrogen storage reactor: Experimental study. Therm. Sci. Eng. Prog..

[327] Ribeiro G.H.A.C., Rouzineau D., Meyer M., Raimondi N.D.M. (2025). Novel design approach based on triply periodic minimal surfaces (TPMS) for gas–liquid contactor packing. Chem. Eng. Sci..

[328] Liu T.Z., Zhao W., Yao Y.T., Lin C., Zhao H.X., Cai J.G. (2024). Mechanical and shape-memory properties of TPMS with hybrid configurations and materials. Int. J. Smart Nano Mat..

[329] Rollo G., Ronca A., Cerruti P., Xia H., Gruppioni E., Lavorgna M. (2023). Optimization of Piezoresistive Response of Elastomeric Porous Structures Based on Carbon-Based Hybrid Fillers Created by Selective Laser Sintering. Polymers.

[330] Su R., Liu P., Chen J., Wang W., Chen X., He R., Li Y. (2025). Self-Assembly and 3D Printing of SiCw@MXene/SiOC Metastructure Toward Simultaneously Excellent Terahertz Electromagnetic Interference (EMI) Shielding and Electron-to-Thermal Conversion Properties. Adv. Funct. Mater..

[331] Saadi O.W., Schiffer A., Kumar S. (2023). Piezoresistive behavior of DLP 3D printed CNT/polymer nanocomposites under monotonic and cyclic loading. Int. J. Adv. Manuf. Tech..

[332] Shah M., Ullah A., Azher K., Rehman A.U., Juan W., Aktürk N., Tüfekci C.S., Salamci M.U. (2023). Vat photopolymerization-based 3D printing of polymer nanocomposites: Current trends and applications. RSC Adv..

[333] McGregor M., Patel S., Zhang K., Yu A., Vlasea M., McLachlin S. (2024). A manufacturability evaluation of complex architectures by laser powder bed fusion additive manufacturing. J. Manuf. Sci. Eng..

[334] Tyagi S.A., Manjaiah M. (2024). Printability, post-processing and mechanical behaviour of sub-millimetre sized SS 17-4PH TPMS lattice structures. Mater. Today Commun..

[335] Li Y., Feng Z., Hao L., Huang L., Xin C., Wang Y., Bilotti E., Essa K., Zhang H., Li Z. (2020). A review on functionally graded materials and structures via additive manufacturing: From multi-scale design to versatile functional properties. Adv. Mater. Technol..

[336] Hasanov S., Alkunte S., Rajeshirke M., Gupta A., Huseynov O., Fidan I., Alifui-Segbaya F., Rennie A. (2021). Review on additive manufacturing of multi-material parts: Progress and challenges. J. Manuf. Mater. Process..

[337] Nazir A., Gokcekaya O., Billah K.M.M., Ertugrul O., Jiang J., Sun J., Hussain S. (2023). Multi-material additive manufacturing: A systematic review of design, properties, applications, challenges, and 3D printing of materials and cellular metamaterials. Mater. Des..

[338] Vora H.D., Sanyal S. (2020). A comprehensive review: Metrology in additive manufacturing and 3D printing technology. Progress Addit. Manuf..

[339] Pei E., Kabir I., Breški T., Godec D., Nordin A. (2022). A review of geometric dimensioning and tolerancing (GD&T) of additive manufacturing and powder bed fusion lattices. Progress Addit. Manuf..

[340] Hossain M.S., Li H., Chowdhury F., Nilufar S. (2025). Analysis of Biobased Composites with Microcrystalline Cellulose Developed by Additive Manufacturing: Hossain, Li, Chowdhury, and Nilufar. JOM.

[341] Zhou J., Gui Y., Xu Q., He L., Long Y. (2024). Investigation of permeability and biocompatibility of TPMS structures printed by laser powder bed fusion using Ti64-5Cu alloy for orthopedic implants. Mater. Lett..

[342] Wakjira Y., Cioni A., Lemu H.G. (2025). Current status of the application of additive-manufactured TPMS structure in bone tissue engineering. Progress Addit. Manuf..

[343] Kokare S., Oliveira J., Godina R. (2023). Life cycle assessment of additive manufacturing processes: A review. J. Manuf. Syst..

[344] Cai Y., Xiong J., Chen H., Zhang G. (2023). A review of in-situ monitoring and process control system in metal-based laser additive manufacturing. J. Manuf. Syst..

[345] Sani A.R., Zolfagharian A., Kouzani A.Z. (2024). Artificial intelligence-augmented additive manufacturing: Insights on closed-loop 3D printing. Adv. Intell. Syst..

[346] Djonyabe Habiba R., Malça C., Branco R. (2024). Exploring the potential of recycled polymers for 3D printing applications: A review. Materials.

